# HLA‐A*02:01 Presents Penicillin‐Modified Cysteinylated Peptides for T Cell Recognition

**DOI:** 10.1111/all.70025

**Published:** 2025-09-04

**Authors:** Shawn J. R. Goh, Hovey H. W. Lu, Katherine E. Scull, Kirti Pandey, Mohammadreza Dorvash, Kirsten Deckert, Robert Puy, Robyn E. O'Hehir, Anthony W. Purcell, Nicole A. Mifsud, Patricia T. Illing

**Affiliations:** ^1^ Immunity Program, Monash Biomedicine Discovery Institute and Department of Biochemistry and Molecular Biology Monash University Clayton Victoria Australia; ^2^ Allergy, Asthma and Clinical Immunology Service Alfred Health Melbourne Victoria Australia; ^3^ Department of Immunology, School of Translational Medicine Monash University Melbourne Victoria Australia

**Keywords:** allergy, HLA, penicillin, T cell‐mediated hypersensitivity, β‐lactam antibiotics

## Abstract

**Background:**

A subset of patients experience immune‐mediated hypersensitivity reactions towards β‐lactam antibiotics, with drug‐specific T cells implicated as one of the causative factors. The principal mechanism is thought to involve chemical haptenation of self‐peptides, resulting in novel peptide drug‐adducts that may trigger T cell recognition. Understanding the interactions between the β‐lactam drug, the T cell receptor (TCR) and the peptide/human leukocyte antigen (pHLA) complex is critical to gain further mechanistic insights into these hypersensitivity reactions. This study aimed to 1) explore the array of haptenated ligands presented by HLA‐A*02:01, 2) evaluate the repertoire of T cells involved in penicillin‐induced reactions in a hypersensitive patient and 3) determine if a dominant penicillin‐specific TCR clonotype recognises haptenated HLA peptides.

**Method:**

An immunopeptidomics approach was applied to identify benzylpenicillin (BP)‐modified peptide ligands within the HLA‐A*02:01 ligandome. The drug‐reactive TCR repertoire was analysed by single‐cell sequencing of CD8^+^ T cells expanded in the presence of BP, and the dominant TCR assayed for reactivity in a reporter cell line.

**Result:**

We report that BP modifies cysteine in preference to lysine residues within the HLA‐A*02:01 immunopeptidome. This modification occurs via cysteine‐drug conjugate formation, in conjunction with disulphide‐mediated peptide modification, which has not previously been considered in the context of drug hypersensitivities. Furthermore, we demonstrate that a BP‐specific TCR expanded from a patient reacts towards a reduction‐sensitive epitope, consistent with a BP‐cysteine adduct disulphide linked to a cysteine residue within the T cell epitope.

**Conclusion:**

Our study provides evidence that cysteine‐penicillin adducts can be accommodated by HLA ligands with the potential to induce T cell‐mediated allergic reactions.

## Graphical abstract

We describe, for the first time, HLA presentation of benzylpenicillin‐modified peptides incorporating a cysteine‐benzylpenicillin conjugate (CysBP), disulfide linked to an in‐chain cysteine. A dominant HLA‐A*02:01 restricted benzylpenicillin‐specific T cell clone, TRAV3/TRBV20‐1 (BP‐TCR), was identified within PBMCs of a penicillin‐hypersensitive patient. Mechanistic investigations demonstrated that the BP‐TCR epitope is sensitive to reduction, consistent with the incorporation of a Cys‐BP modification.

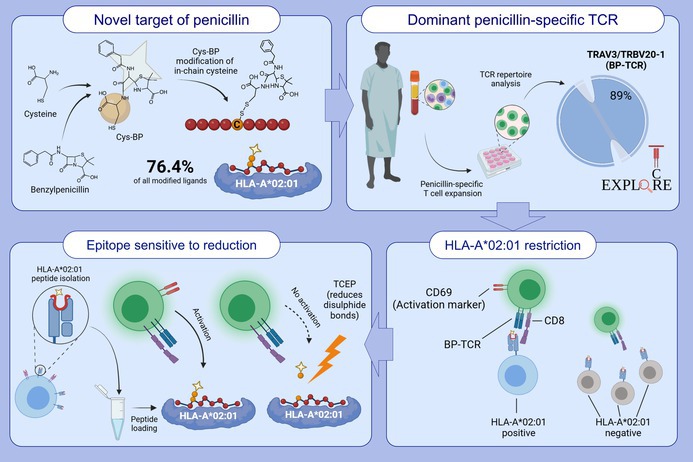

**Abbreviations**
BP
: benzylpenicillin
Cys: cysteine
HLA
: human leukocyte antigen
PBMCs
: peripheral blood mononuclear cells
TCEP
: tris(2‐carboxyethyl)phosphine
TCR
: T cell receptor
TRAV3
: T cell receptor alpha variable 3
TRBV20‐1: T cell receptor beta variable 20–1

## 1 Introduction

In Australia, 25% of hospitalised patients have documented reports of adverse drug reactions towards antimicrobial drugs, with 54% caused by β‐lactam antibiotics such as penicillin [1]. Adverse drug reactions towards penicillin can be mediated by IgE, inducing immediate drug hypersensitivity reactions (DHRs) such as anaphylaxis [2, 3]. Alternatively, delayed DHRs can be mediated by T cells, with symptoms occurring within hours following drug re‐administration, or spanning from a few days to a month after the initial drug exposure [3, 4]. Delayed DHRs caused by penicillin can present as a range of clinical pathologies, including cutaneous reactions ranging from maculopapular exanthema to more severe and life‐threatening skin manifestations such as Stevens–Johnson syndrome/toxic epidermal necrolysis, as well as organ damage such as drug‐induced liver injury [4]. Such hypersensitivity reactions to penicillin not only mandate clinicians to prescribe an alternate antibiotic that might be suboptimal for bacterial clearance, but also drive the evolution and adaptation of bacteria to antibiotics—contributing to global concerns of antimicrobial resistance [5]. Moreover, inappropriate use of alternative antibiotics can lead to additional treatments due to increased side effects, resulting in longer hospital stays and raising healthcare costs [6].

Penicillins contain a highly reactive β‐lactam ring that facilitates haptenation of macromolecules thought primarily to occur by the covalent attachment of this chemical warhead to free amine groups, such as in the side chain of lysine residues [7, 8]. Both extracellular [9, 10, 11, 12, 13] and intracellular proteins [14, 15, 16] can be targeted for haptenation; thus, haptenated peptides are potentially available for antigen processing and presentation by HLA molecules for T cell surveillance. To better understand T cell reactivity towards penicillin‐modified HLA ligands, others have used designer penicillin‐modified peptides which induced CD4^+^ and CD8^+^ T cell proliferation, and Th1 and Th2 cytokine responses by CD4^+^ T cells [12, 16, 17, 18, 19]. Naturally presented β‐lactam‐modified ligands identified using immunopeptidomics have recently been reported [15, 20], collectively identifying seven unique flucloxacillin‐modified ligands presented by HLA‐B*57:01. Moreover, using a HLA‐B*57:01 transgenic mouse model, Puig et al. further demonstrated CD8^+^ T cell reactivity towards an experimentally identified flucloxacillin‐modified peptide [20], thereby establishing capacity for immunogenicity of such modified epitopes. However, there is a lack of information on naturally presented, drug‐modified peptides across the breadth of penicillins and HLA allotypes.

Due to the prevalence of HLA‐A*02:01 [21] and its known association with penicillin‐induced DHRs [22], we investigated the BP‐induced immunopeptidome of HLA‐A*02:01 to determine the array of drug‐modified peptides presented for T cell surveillance. Here, we report for the first time, to the best of our knowledge, naturally presented HLA‐A*02:01‐restricted β‐lactam‐modified ligands, representing the most extensive database of β‐lactam‐modified peptides reported to date. Importantly, we demonstrated that most modifications occurred through a drug‐cysteine adduct forming a disulphide bond with free cysteine residues of bound peptides, representing over 76% of all BP‐modified ligands identified. We further demonstrated that a dominant HLA‐A*02:01 restricted TCR clonotype, identified within peripheral blood mononuclear cells (PBMCs) of a patient with resolved penicillin‐induced DHR, recognises an epitope that is sensitive to reduction, consistent with a peptide containing a BP‐modified cysteinylated cysteine residue. Together, these findings highlight the immunological relevance of drug‐modified post‐translational modifications (PTMs) as potential triggers for β‐lactam‐induced T cell responses.

## 2 Materials and Methods

A description of the methods to evaluate the array of peptides presented by HLA‐A*02:01 is available in Appendix S3. Patient information, methods used to evaluate the BP‐specific TCR repertoire within the patient sample and functional T cell assays are also available in Appendix S3. For T cell assays, the Student's t test was used to compare the significance between test and control samples and reported as mean ± standard error of mean (SEM). All mass spectrometry data have been deposited to the ProteomeXchange via the PRIDE partner repository [23] with the identifier PXD057177 and PXD065200.

## 3 Results

### 3.1 Penicillin Treatment Does Not Alter the Binding Characteristics of HLA‐A*02:01 Bound Peptide Ligands

Small molecule drugs such as abacavir and flucloxacillin have been reported to perturb the binding motif observed for peptide ligands of HLA‐B*57:01 [20, 24, 25, 26]. Therefore, using mass spectrometry, we examined the naturally presented peptides bound to HLA‐A*02:01 following cellular BP treatment to assess whether penicillin treatment has a similar impact [27, 28]. Membrane‐bound HLA‐A*02:01 complexes were immunopurified from 4x10^9^ C1R.A*02:01 cells (either untreated or BP‐treated) with an anti‐HLA‐A2 antibody (BB7.2) [29] and the extracted peptide ligands fractionated by RP‐HPLC. Half of this material was analysed by liquid chromatography with tandem mass spectrometry (LC–MS/MS) using higher energy collisional dissociation‐based fragmentation. The remainder was reserved for immunogenicity assays.

**FIGURE 1:**
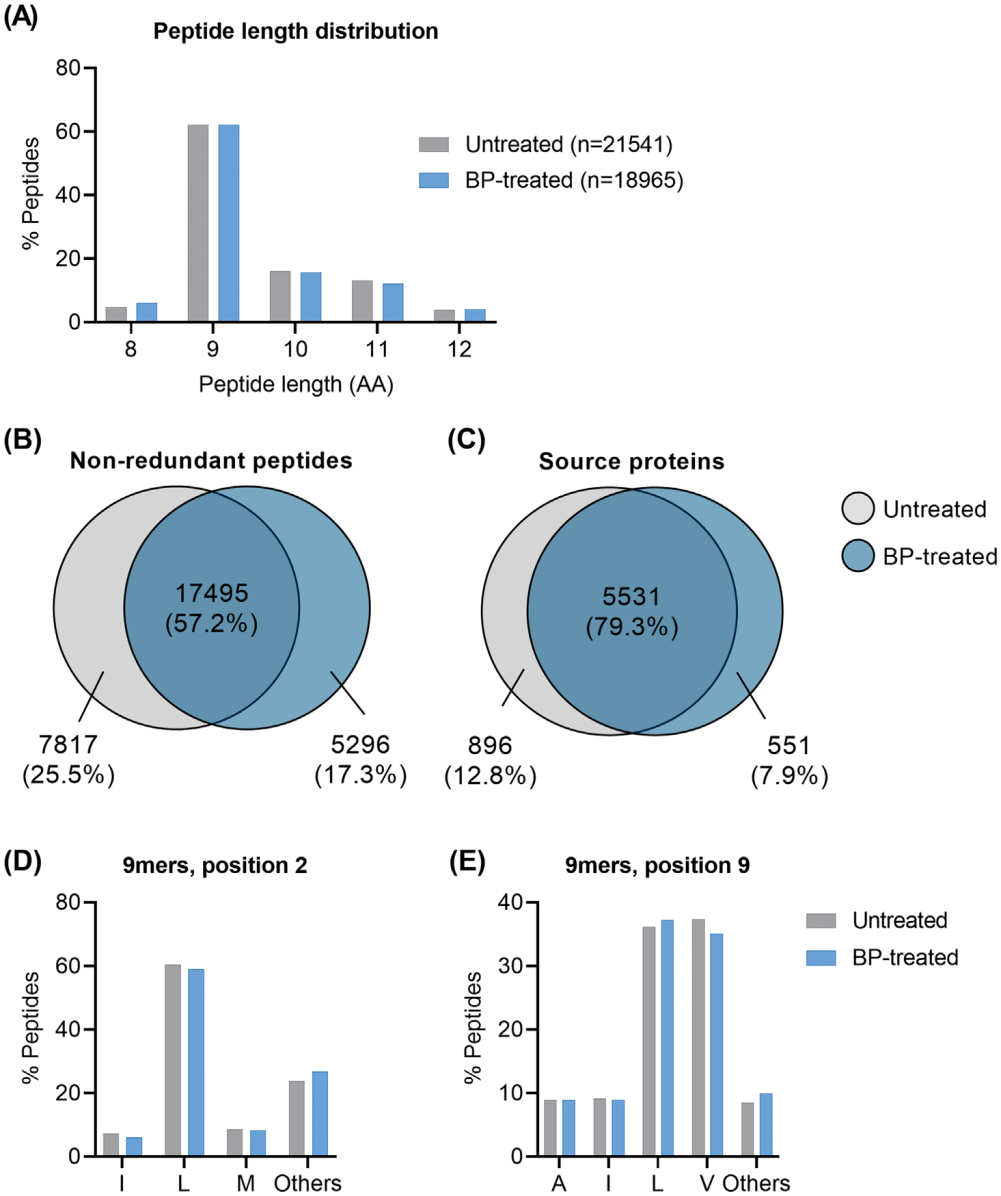
Characteristics of the HLA‐A*02:01 immunopeptidome. (A) Peptide length distribution of non‐redundant (unique by sequence) HLA‐A*02:01 ligands isolated from C1R.A*02:01 with the anti‐HLA‐A2 antibody, BB7.2. (B, C) The overlap in (B) HLA‐A*02:01 bound peptides identified from untreated and BP treated C1R.A*02:01 cells and (C) their source proteins. (D, E) The occupancy of amino acid residues at anchor positions P2 and P9 of 9mer peptides identified with a 5% peptide FDR cut‐off.

A total of 21,541 and 18,965 non‐redundant peptide sequences of 8–12 amino acids in length were identified from untreated and BP‐treated C1R.A*02:01 cells, respectively. A comparison of peptides isolated under these conditions suggests no change in peptide length distribution, with predominantly nine amino acid long peptides observed for both conditions (Figure 1A) and an overlap of 17,495 peptides (57.16%), with 7817 sequences (25.54%) uniquely identified in the untreated sample and 5296 sequences (17.3%) uniquely identified in the BP‐treated sample (Figure 1B). These peptides mapped to a total of 6978 source proteins, 5531 common between the conditions, 896 only identified in the untreated sample and 551 only identified in the BP‐treated sample (Figure 1C). Importantly, we did not observe an impact of drug treatment on the HLA‐A*02:01 preferred anchor residues at P2 or P9 of the 9mer peptides (Figure 1D,E) [30, 31]. The maintenance of leucine at P2, and leucine/isoleucine at PΩ was also observed for 10mer and 11mer peptides (Figure S1, Appendix S2) Collectively, our data suggest that BP treatment of antigen‐presenting cells (APCs) does not facilitate loading of peptides with an altered binding motif.

### 3.2 Identification of Novel BP‐Modified Peptides Presented by HLA‐A*02:01

**FIGURE 2:**
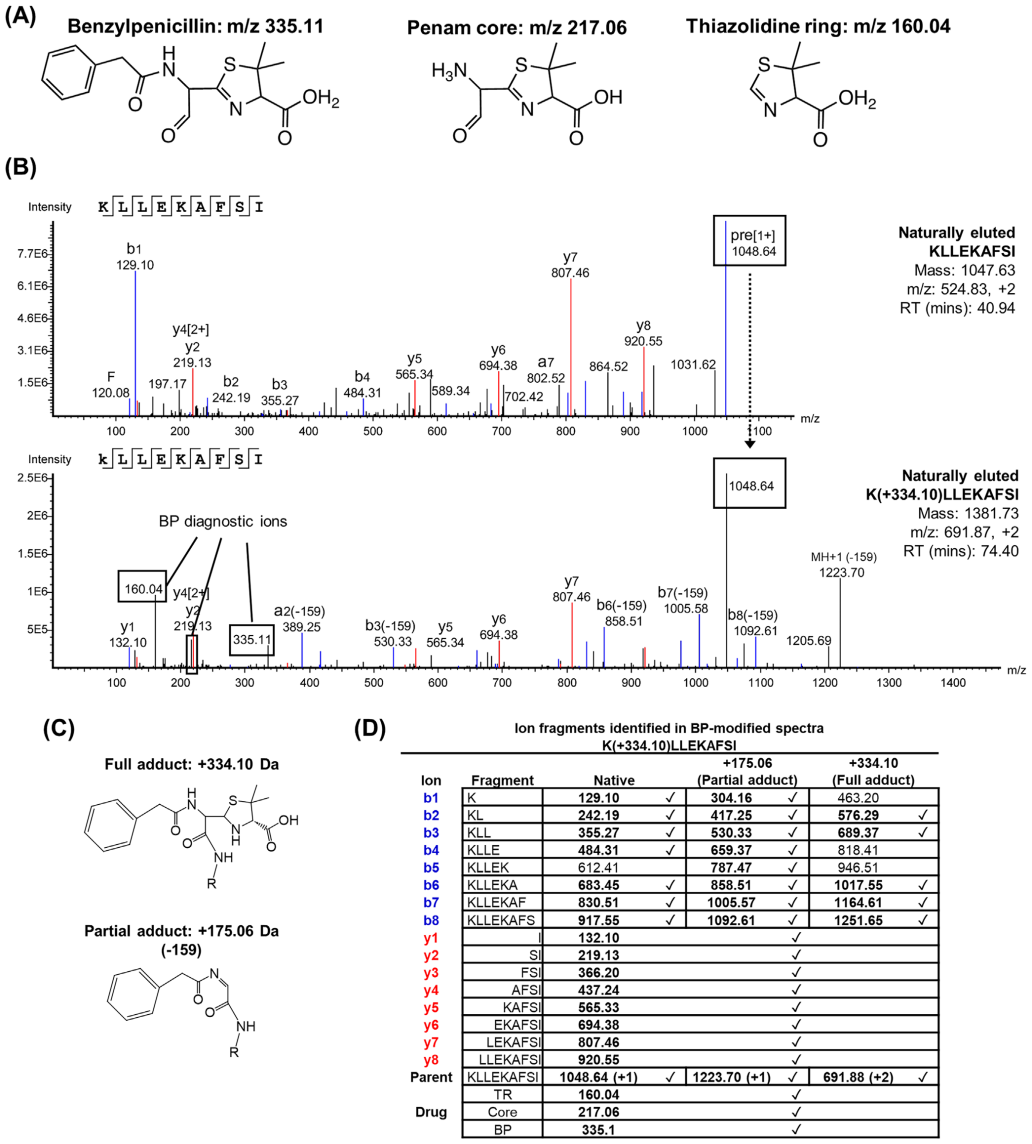
Key features of a fragmentation spectrum of a BP‐modified peptide. (A) *m/z* of diagnostic fragment ions and chemical structures of BP and its fragments generated by Higher energy Collisional Dissociation (HCD)‐based fragmentation. (B) Representative spectrum of the native peptide KLLEKAFSI (top) and its BP‐haptenated counterpart K(+334.1)LLEKAFSI (bottom). The BP‐haptenated peptide has a mass shift of +334.10 Da and contains diagnostic BP‐related fragment ions (*m/z* 160.04, 217.06, 335.11). (C) Chemical structures of full BP adduct (+334.10 Da) and partial BP adduct after cleavage of the thiazolidine ring (+175.06 Da), via a free amine represented by NH‐R. (D) Table listing the b and y ions from the native peptide, peptide containing the partial adduct (+175.06 Da) and the full BP adduct (+334.10 Da) within the spectrum of K(+334.10)LLEKAFSI.

BP‐modified peptides are characterised by a mass addition of 334.10 Da and MS/MS spectra containing BP‐specific diagnostic fragment ions (BP: *m/z* 335.11, Penam core: *m/z* 217.06, Thiazolidine ring: *m/z* 160.04) owing to drug cleavage and fragmentation (Figure 2A). An example of fragmentation of a BP‐modified HLA‐A*02:01 peptide ligand is depicted in Figure 2B, which shows the fragmentation spectrum of K(+334.10)LLEKAFSI (*m/z* = 691.87, z = 2) in comparison with the native peptide KLLEKAFSI (*m/z* = 524.83, z = 2). In addition to the low mass BP diagnostic fragment ions, there is a b ion series for the peptide backbone retaining the partial mass of BP (+175.06 Da, Partial adduct) and the full mass of BP (+334.10 Da, Full adduct) on the modified lysine at position 1 (Figure 2C,D). In contrast, C‐terminal y1‐8 ions, which lack the modified lysine, match those of the native peptide (Figure 2D). The spectrum of K(+334.10)LLEKAFSI also contains ions representing the full‐length peptide with a partial adduct (loss of thiazolidine ring: −159.04 Da, *m/z* 1223.70) and a complete loss of BP (−334.10 Da, *m/z* 1048.64) (Figure 2B).

To identify BP‐modified peptides within our immunopeptidome data, we initially considered mass additions of 334.10 Da on lysine, arginine, cysteine and histidine residues based on previous reports [15, 32]. However, we noted that a number of spectra containing BP‐specific diagnostic fragment ions were unable to be annotated using these criteria. Manual inspection of one of these spectra using PEAKS de novo software indicated fragmentation patterns consistent with a peptide beginning with LLPPP. A search of the Immune Epitope Database for consistent HLA‐A*02:01 ligands identified LLPPPPCPA, with and without a cysteinylated cysteine at position 7, as a potential match [33]. Notably, the mass of the unassigned spectra was consistent with the cysteinylated peptide LLPPPPC(+119.00)PA with an additional mass addition of 334.10 Da, that is LLPPPPC(+453.10)PA. Hence, we speculated that the free amine of the cysteinylated cysteine was a target of BP haptenation, a reaction described for free cysteine and BP [34].

**FIGURE 3:**
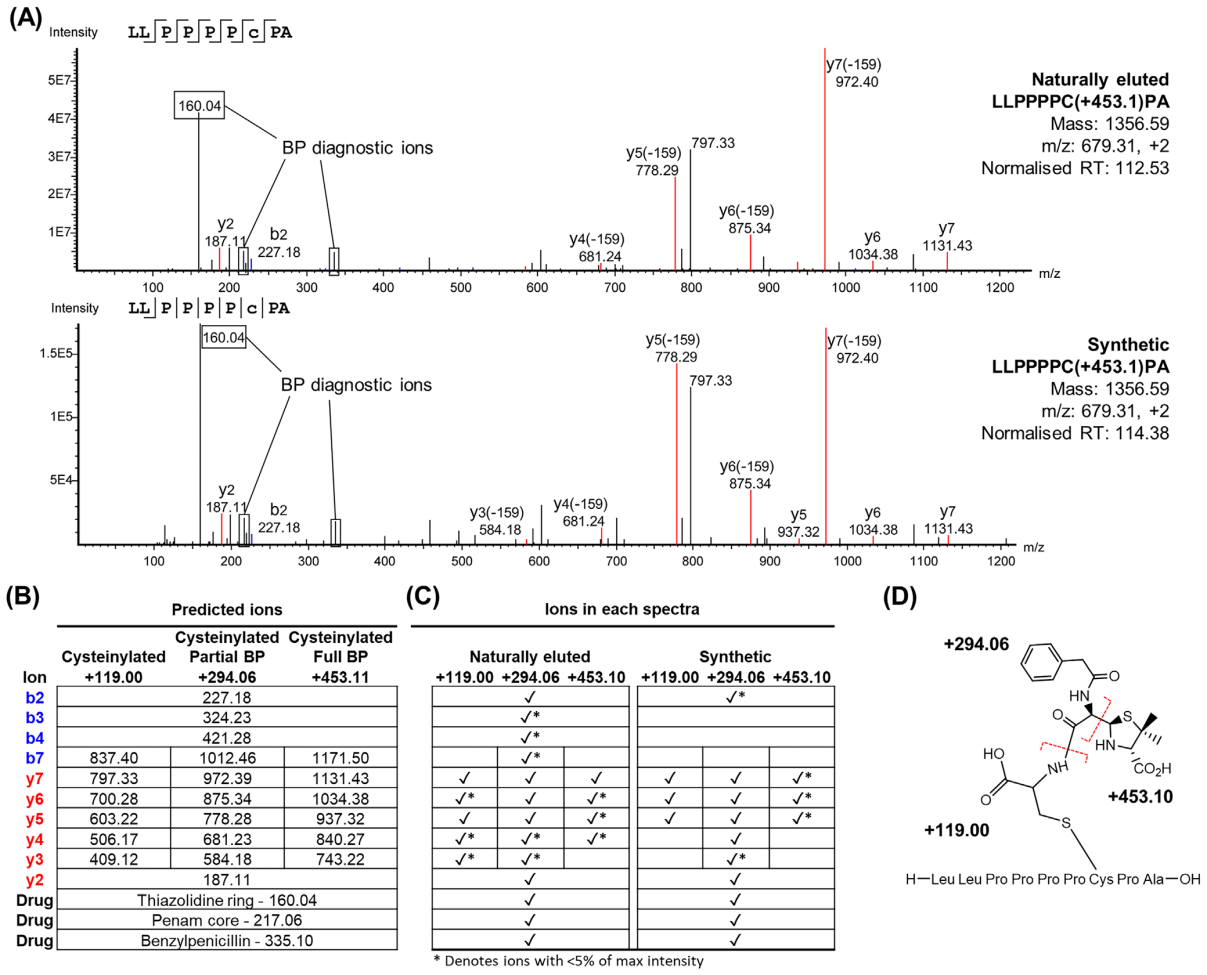
Identification of haptenated cysteinylated peptide LLPPPPC(+453.10)PA presented by HLA‐A*02:01. (A) Spectrum of naturally eluted peptide LLPPPPC(+453.10)PA (top), and the retrospectively synthesised LLPPPPC(+453.10)PA peptide (bottom). (B) Table listing the predicted *m/z* values of b and y ions from LLPPPPC(+453.10)PA containing cysteinylated cysteine (+119.00), cysteinylated cysteine with a thiol linked partial BP adduct (+294.06) or cysteinylated cysteine with a full BP adduct (+453.10). (C) Table indicating the presence of these ions within the spectra of either the naturally eluted or synthetic LLPPPPC(+453.10)PA. (D) CysBP fragmentation pattern and corresponding mass addition onto the native peptide.

To verify the assignment of LLPPPPC(+453.10)PA as a peptide incorporating BP via a disulphide linked Cysteine (CysBP), we generated a synthetic analogue by incubating native LLPPPPCPA with L‐cysteine, followed by BP. LC–MS/MS analysis showed highly correlated fragmentation patterns between the eluted and synthesised peptide with a Pearson's correlation coefficient score (PCC) of 0.96 (Figure 3A,B,C). Both naturally eluted and synthetic analogues had similar normalised retention times (Naturally eluted: 112.53, Synthetic: 114.36) (Figure 3A). The proposed fragmentation of CysBP is depicted in Figure 3D. Furthermore, additional fragmentation of the synthesised peptide using alternative fragmentation modalities incorporating electron activated dissociation (EAD) provided evidence of the intact CysBP adduct (Figure S2, Appendix S2), further verifying the structure of the modification.

Based on this evidence, we subsequently incorporated additional modifications of cysteinylation alone (+119.00) and CysBP (+453.10) at cysteine residues in our MS/MS data search parameters. To aid high‐throughput verification of possible BP‐modified peptides, we developed PenicillinFinder, a script to recognise BP‐specific diagnostic fragment ions (BP: *m/z* 335.11, Penam core: *m/z* 175.06, Thiazolidine ring: *m/z* 160.04) within MS/MS peptide spectra to substantiate the modification assignment made by PEAKS Xpro software (Figure 2A).

Of the 487 spectra assigned a BP modification by PEAKS Xpro (at a 5% peptide false discovery rate [FDR]), 253 spectra contained the *m/z* 160.04 diagnostic ion and at least one other penicillin‐related fragment ion at *m/z* 217.06 or 335.11 amu. These spectra were annotated as 55 unique high‐confidence BP‐modified peptides by sequence and modification site, whilst a further 7 BP‐modified peptide sequences were identified with insufficient spectral information to confidently determine the modified amino acid (i.e., ambiguous haptenation site) (Appendix S1).

Synthesis of a second peptide containing CysBP, SLHDALC(CysBP)VV, provided further validation of this modification. A comparison of all ions within the naturally eluted and synthetic peptide spectra results in a PCC score of 0.85. Importantly, we were able to identify b and y ions with the loss of the thiazolidine ring and BP itself within both experimentally identified and synthetic peptide spectra, and BP‐specific diagnostic ions were also present in both spectra (Figure S3, Appendix S2).

**FIGURE 4:**
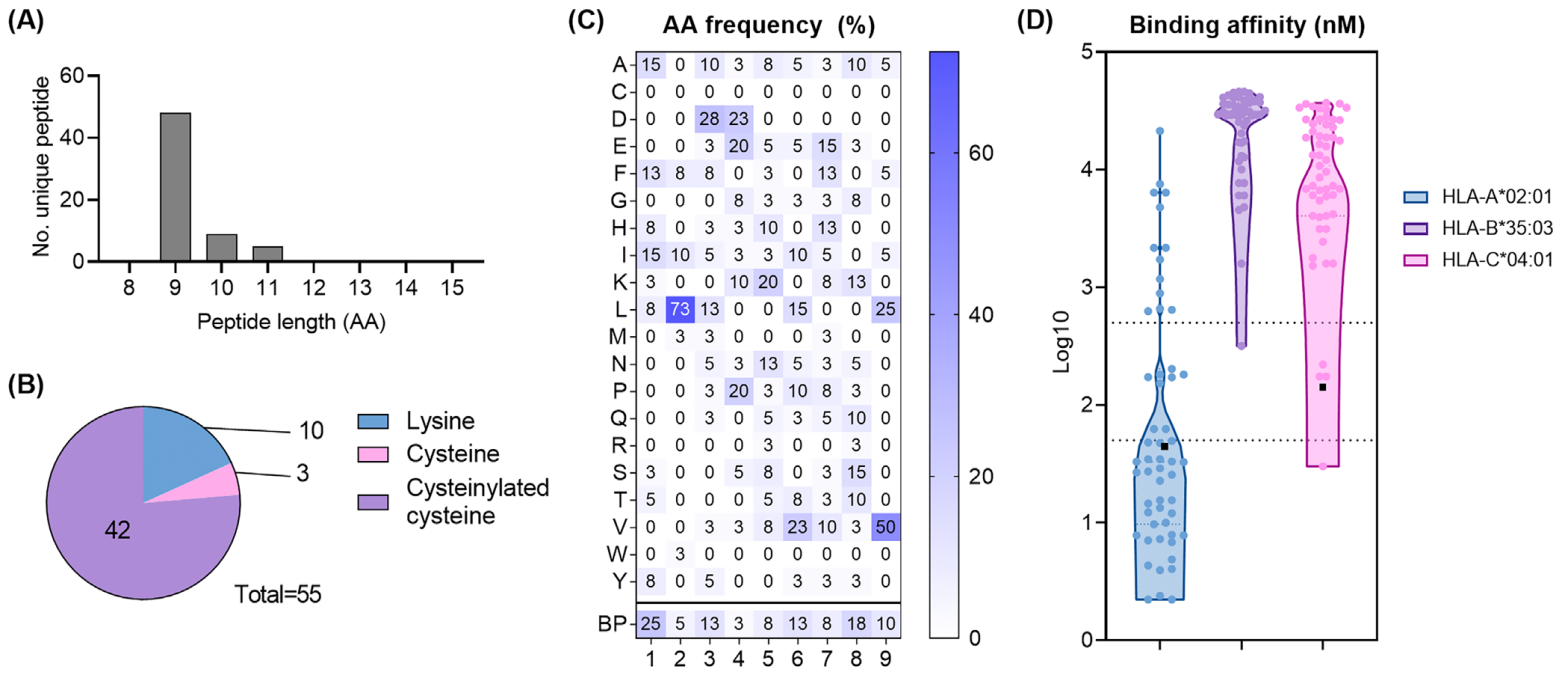
Identification and characterisation of novel BP‐modified peptides identified within the HLA‐A*02:01 immunopeptidome. (A) Peptide length distribution of benzylpenicillin‐modified peptides that are unique by sequence and modification. (B) Number of peptides with non‐ambiguous haptenation sites identified to carry a benzylpenicillin modification at either lysine, cysteine or cysteinylated cysteine. (C) Amino acid frequency of benzylpenicillin‐modified peptides with unambiguous haptenation sites. (D) Predicted binding affinity (nM) of benzylpenicillin‐modified peptides for HLA‐A*02:01, HLA‐B*35:03 and HLA‐C*04:01. Black squares denote FMDESTQCF that was predicted to bind both HLA‐A*02:01 and HLA‐C*04:01.

Most BP‐modified peptides (48/62) were nine amino acids in length (Figure 4A, Table S1, AppendixS4). Among the 55 peptides with unambiguous haptenation sites, 42 peptides contained CysBP, 10 directly modified lysine and 3 directly modified cysteine (Figure 4B). 9mer peptides with unambiguous haptenation sites retained enriched leucine at P2, as well as leucine and valine at P9 (Figure 4C). We also noted that most BP modifications on 9mers were either at position 1 or 8 of the bound peptide, which are usually solvent exposed and therefore potentially recognisable by T cells (Figure 4C) [35]. Using NetMHCpan‐4.1, the majority of peptides with unambiguous haptenation sites were predicted to bind HLA‐A*02:01 in their unmodified state with stronger than 500 nM affinity, while one and four were predicted to bind to the endogenous HLA allomorphs expressed by C1R HLA‐B*35:03 and HLA‐C*04:01, respectively (Figure 4D) [36]. Of note, peptide FM(Oxidised)DESTQC(CysBP)F was predicted to bind both HLA‐A*02:01 and HLA‐C*04:01 (Figure 4D, black squares).

### 3.3 Haptenation of Cysteinylated Cysteines Is Not Unique to BP Among β‐Lactam Antibiotics

Given the high frequency of complex CysBP‐modified peptides identified in this study, we speculated that other β‐lactam antibiotics also known to induce haptenation, such as flucloxacillin, may also induce such modifications. We reinterrogated our previously published HLA‐B*57:01 immunopeptidome data generated from flucloxacillin‐treated cells [15]. Consistent with Waddington et al. [15], peptides were considered flucloxacillin‐modified if spectra contained the diagnostic fragment ion at *m/z* 160.04 amu. Furthermore, by incorporating cysteine cysteinylation (+119) and flucloxacillin‐modified cysteinylated cysteine (CysFlux) (+572.06) as variable modifications, we identified 12 flucloxacillin‐modified peptides unique by sequence and modification. For 9/12 peptides, the haptenation site was unambiguous, of which seven accommodated CysFlux modifications, one a flucloxacillin modification directly on a backbone cysteine residue, and one a flucloxacillin modification directly on lysine (Table S2, Appendix S4).

We noted that certain peptide spectra originally assigned to sequences incorporating a C‐terminal alanine residue after an aromatic residue and a mid‐sequence tri‐oxidated (O3) cysteine were alternatively explained by CysFlux modification. For instance, the precursor *m/z* 533.86 (2+) assigned HTAHIAC(O3)K(flucloxacillin)FA in Waddington et al. [15] was assigned HTAHIAC(CysFlux)KF in this study. Although these two sequence assignments fit the molecular weight of the precursor ion, we postulate that the sequence assigned in this study is more likely due to the presence of a b7 ion from HTAHIAC(CysFlux)KF with the loss of the thiazolidine ring and the absence of the b8 ion from HTAHIAC(O3)K(flucloxacillin)FA (Figure S4, Appendix S2).

### 3.4 Clonal Expansion of a BP‐Specific CD8 ^+^ T Cell From a Penicillin‐Hypersensitive Patient

**FIGURE 5:**
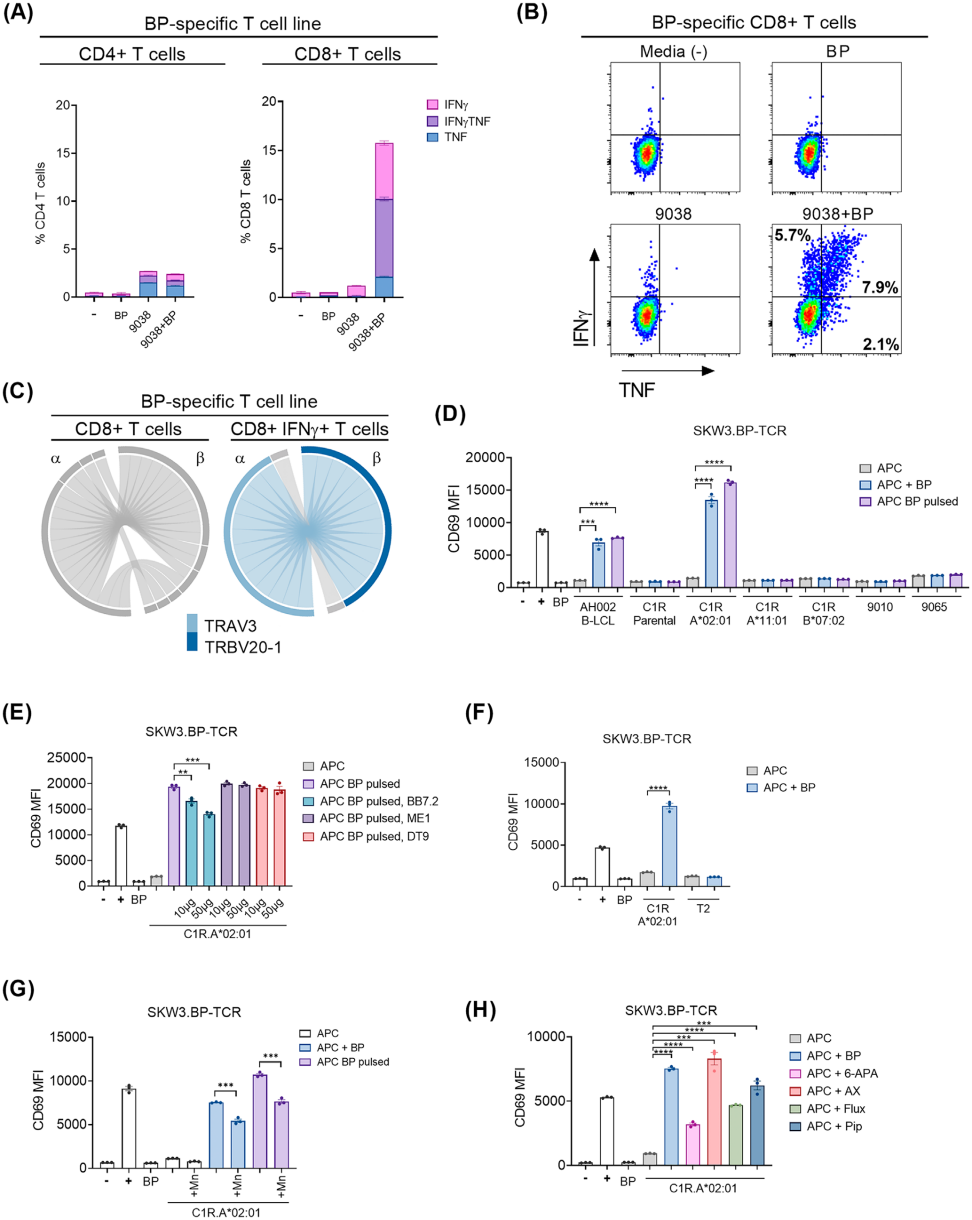
Identification of a dominant BP‐specific CD8^+^ TCR. (A, B) PBMCs derived from patient AH002 were cultured with 0.5 mM of BP for 28 days. T cell subsets (CD4^+^ and CD8^+^) and activation (IFNγ^+^ or TNF^+^) of the BP‐expanded cell line were measured after a 6‐h restimulation with 9038 B‐LCL in the presence or absence of 0.5 mM BP. (A) The data are summarised as stacked bar graphs expressed as mean ± SEM of duplicate measurements. (B) Representative flow cytometry plots of intracellular cytokine staining are shown for CD8^+^ T cell responses. (C) Circos plots for d28 CD8^+^ T cell TCR sequences from patient AH002, characterised after stimulation with 9038, showing a clonally expanded αβ TCR pairing for the CD8^+^ IFNγ^+^ T cell subset. Complete TCR sequencing can be found in Table S6, Appendix S4. (D–H) Activation assays measuring CD69 surface upregulation of SKW3.BP‐TCR in response to (D) a panel of HLA‐matched APCs (HLA typing Table S3, Appendix S4): APC‐only (APC), APC with 0.5 mM benzylpenicillin in the stimulation mix (APC + BP), benzylpenicillin‐pulsed APCs (APC BP pulsed), (E) C1R.A*02:01 blocked with HLA subtype‐specific antibodies, (F) a TAP‐negative HLA‐A*02:01^+^ cell line (T2), (G) C1R.A*02:01 cells treated with monensin prior to BP treatment and SKW3.BP‐TCR activation and (H) C1R.A*02:01 in the presence of 0.5 mM of either 6‐aminopenicillanic acid (6‐APA), amoxicillin (AX), flucloxacillin (Flux) or piperacillin (Pip). All CD69 upregulation assays were conducted with within assay technical triplicates, mean ± SEM (**p < 0.01, ***p < 0.001 and ****p < 0.0001). ‐: Media only, +: Anti‐CD3/CD28 beads. MFI: Mean fluorescence intensity.

To characterise reactivity towards BP‐modified peptides presented by HLA‐A*02:01, we explored BP‐specific T cell recognition using PBMCs from a penicillin‐hypersensitive patient who had experienced body swelling and angioedema (AH002). At the time of blood donation, the patient was not presenting clinical symptoms; therefore, we were likely measuring recall responses from drug‐exposed memory T cells. T cells from PBMCs expanded towards 0.5 mM BP were restimulated with the 9038 B‐lymphoblastoid cell line (B‐LCL) (HLA‐A*02:01 and HLA‐DQB1*03:01 positive, mismatched for all other HLA allotypes of the AH002 donor) and assayed for the production of proinflammatory Th1 cytokines, IFNγ and TNF. The 9038 APC with the addition of BP stimulated a significantly greater CD8^+^ T cell response than 9038 alone, while no BP‐specific CD4^+^ T cell response was observed (although this may be due to mismatch for HLA class II outside of HLA‐DQB1*03:01) (Figure 5A, Table S3, Appendix S4). The majority of drug‐specific CD8^+^ T cells were dual IFNγ^+^TNF^+^ (7.94% ± 0.20), followed by IFNγ^+^ (5.73% ± 0.23) and to a lesser extent TNF^+^ (2.11% ± 0.07) (Figure 5A,B).

The αβTCR repertoire of the drug‐expanded PBMC was evaluated by single cell sorting the CD8^+^ T cell population based on IFNγ production in response to stimulation with BP, followed by TCR sequencing [37, 38]. Expansion of a dominant BP‐specific clonotype was identified within the activated CD8^+^IFNγ^+^ T cell population (*n* = 14, 92.9%). The TCR expressed by this expanded population of T cells comprised of TRAV3‐CDR3α CAVRDNRNYGQNFVF‐TRAJ26‐TRBV20‐1‐CDR3β CSARTDREGQPQHF‐TRBJ1‐5, and is subsequently designated ‘BP‐TCR’ (Figure 5C). Notably, this αβTCR pairing was not present in the non‐responsive CD8^+^IFNγ^−^ T cell population (*n* = 19).

### 3.5 BP‐Specific TCR Recognises an HLA‐A*02:01‐Restricted BP‐Modified Peptide

We transduced cDNA encoding the full‐length BP‐specific TCR α and β chains into a TCR‐null reporter cell line, SKW3.hCD8αβ [39, 40], to generate the T cell reporter SKW3.BP‐TCR cells. A patient autologous B‐lymphoblastoid cell line (AH002 B‐LCL) in the continuous presence of BP, or after pulsing with BP, induced significant activation (CD69 upregulation) of SKW3.BP‐TCR when compared to AH002 B‐LCL alone (Figure 5D). Furthermore, using a panel of partially HLA‐matched APCs (Table S3, Appendix S4), T cell activation was only recapitulated by the HLA‐A*02:01^+^ line, C1R.A*02:01, confirming the HLA‐A*02:01 restriction of this TCR (Figure 5D). In agreement, anti‐HLA‐A2 (BB7.2) antibody blocking of HLA‐A*02:01 molecules of BP‐pulsed C1R.A*02:01 significantly inhibited SKW3.BP‐TCR activation. Antibodies specific for other HLA class I molecules (ME1 for HLA‐B*07:02, DT9 for HLA‐C allotypes) had no effect on T cell activation (Figure 5E).

The absence of SKW3.BP‐TCR reactivity towards the transporter associated with antigen processing (TAP)‐deficient HLA‐A*02:01^+^ APC T2 in the presence of BP suggested that the epitope is formed through modification of a TAP‐dependent peptide (Figure 5F). The involvement of the antigen processing pathway and peptide loading in the endoplasmic reticulum (ER) was further supported by the observation that monensin, which inhibits protein egress from the ER, significantly inhibited SKW3.BP‐TCR activation (Figure 5G).

Consistent with previous reports of T cell cross‐reactivity between penicillins, the BP‐TCR was also activated by C1R.A*02:01 in the presence of 6‐aminopenicillanic acid, amoxicillin, flucloxacillin and piperacillin (Figure 5H).

### 3.6 BP‐TCR Is Reactive to a Reduction‐Sensitive Modification

**FIGURE 6:**
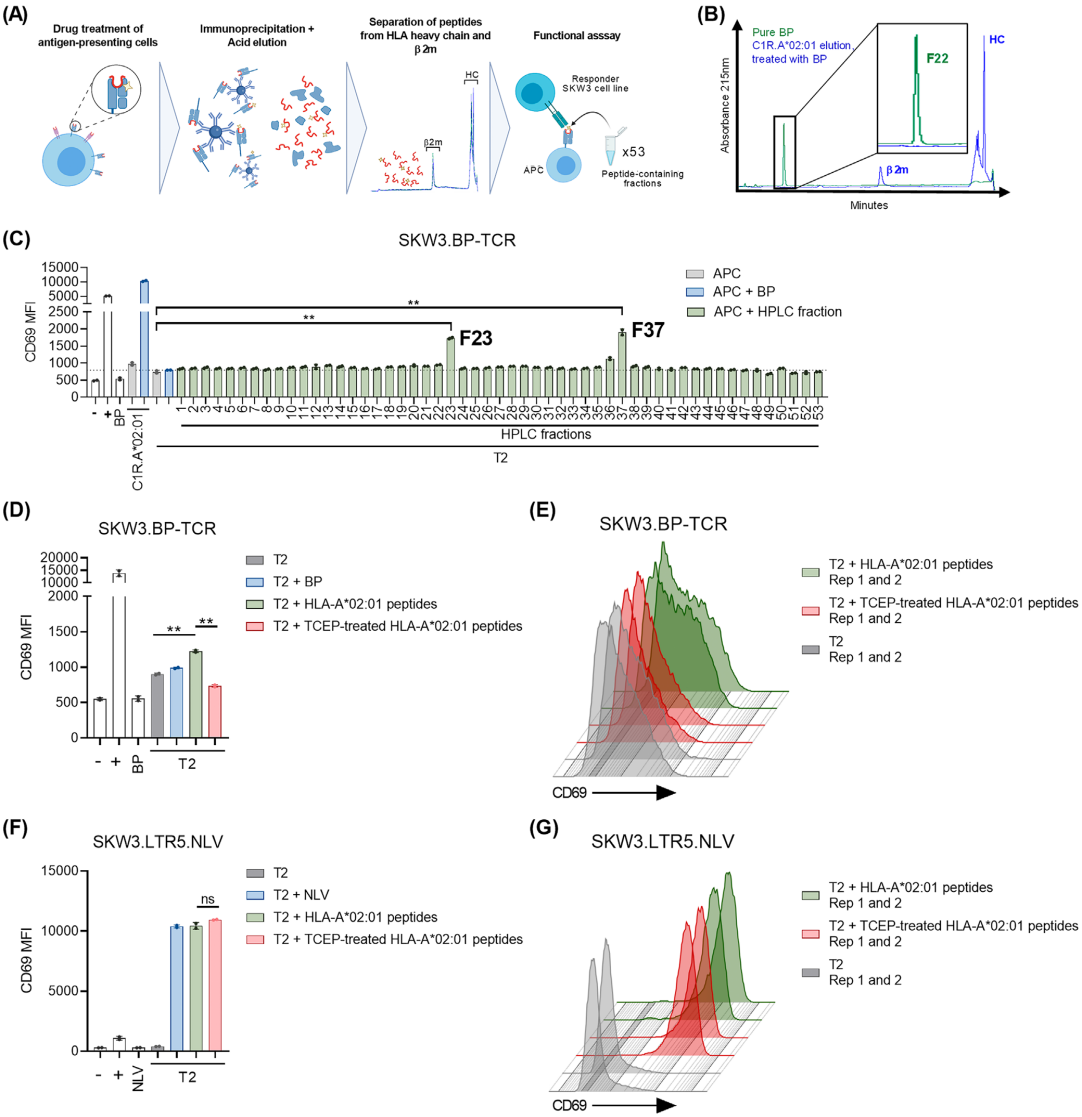
The BP‐TCR recognises a reduction‐sensitive peptide isolated from immunoaffinity captured HLA‐A*02:01 complexes. (A) Workflow to isolate HLA peptides from APCs used to stimulate SKW3.BP‐TCR (Created in BioRender: Mifsud, N. (2025) https://BioRender.com/kztp572)[Correction added on 11 September 2025, after first online publication: BioRender citation for figure 6 (a) has been included]. (B) Pure benzylpenicillin was loaded and separated via RP‐HPLC using the same protocol for peptide separation and visualised by UV absorption at 215 nm. (C–G) Activation assays measuring CD69 surface upregulation of SKW3.BP‐TCR and (C–E) SKW.LTR5.NLV reporter cells (F, G) in response to T2 loaded with fractionated HLA‐A*02:01‐bound peptides. (C) Fractions 23 and 37 obtained from HPLC separation of C1R.A*02:01 peptides could induce SKW3.BP‐TCR activation. (D, E) TCEP‐treatment of HLA‐A*02:01 peptides isolated from BP‐treated C1R.A*02:01 abrogated CD69 upregulation of SKW3.BP‐TCR (response to immunogenic pool shown, responses to other pools are found in Figure S6, Appendix S2). As a control, we demonstrated that SKW.LTR5.NLV activation by the same materials spiked with the cognate NLV epitope was not affected by TCEP treatment. These experiments were conducted with within assay duplicates, mean ± SEM (**p < 0.01, ns = not significant). ‐: Media only, +: anti‐CD3/CD28 bead stimulation. MFI: Mean fluorescence intensity. β2m—β‐2‐microglobulin, HC—heavy chain.

To further support the nature of the activating ligand as an HLA‐A*02:01 restricted peptide, SKW3.BP‐TCR were stimulated with T2 loaded with fractionated peptides isolated from HLA‐A*02:01 molecules purified from BP‐treated C1R.A*02:01 cells. A total of 53 peptide‐containing fractions before the β2m fraction were assayed (Figure 6A,B). The elution time of BP itself was also determined using the same RP‐HPLC method to distinguish potential activity of free BP eluted from HLA‐A*02:01 from that of modified peptides (Figure 6B). The BP‐TCR was significantly activated by fractions 23 and 37 (Figure 6C), while no above‐background response was observed to fraction 22 where free BP was shown to elute (Figure 6B). Lack of SKW3.BP‐TCR activation by T2 in the presence of free BP further supported the hypothesis that activity within the fractions was mediated by BP‐modified peptides, not free BP (Figure 5F). Of note, the BP‐TCR was not reactive towards fractions 23 and 37 isolated from untreated C1R.A*02:01 (Figure S5, Appendix S2).

To determine if the activating ligands were sensitive to reduction, naturally presented peptides isolated and fractionated from HLA‐A*02:01 molecules of BP‐treated and untreated C1R.A*02:01, and formed into nine pools per condition, were loaded onto T2 cells with or without pre‐treatment with TCEP to reduce cysteine residues and remove any CysBP modifications. A single pool from BP‐treated cells induced SKW3.BP‐TCR activation in the absence of TCEP, a response that was abrogated by TCEP treatment (*p* = 0.0014) (Figure 6D,E, Figure S6, Appendix S2). Importantly, HLA‐A*02:01 peptide samples were spiked with the control CMV phosphoprotein 65‐derived epitope (NLVPMVATV) for stimulation of SKW3.LTR5.NLV cells (control cell line, NLV‐specific [41]). Here, as anticipated, TCEP did not impact SKW3.LTR5.NLV activation, indicating that TCEP does not broadly interfere with peptide loading of T2 cells or TCR activation (Figure 6F,G). These results suggest that the BP‐TCR recognises a peptide containing a reduction‐sensitive modification consistent with the previously determined CysBP adduct.

Taking into consideration that SKW3.BP‐TCR is reactive to a reduction‐sensitive epitope in fractions 23 and 37, we then analysed the remainder of these two fractions by mass spectrometry. We identified four CysBP ligands in fraction 23 and one in fraction 37 (Table S4, Appendix S4). However, synthesis of these ligands in the required amounts and purity for immunogenicity assays was not feasible in this study.

### 3.7 Formation and Presentation of CysBP Adducts Might Not Require Antigen Processing

CysBP modification accounted for most of the identified BP‐modified ligands within the HLA‐A*02:01 immunopeptidome (Figure 4B). However, free cysteine is present in cell culture media and can potentially be modified by BP, forming CysBP extracellularly and preceding protein modification. Based on a report by Ono et al. demonstrating the potential of CysBP formation in cell culture media, we analysed and detected CysBP in the media used to culture C1R.A*02:01 when BP was added (Figure S7, Appendix S2). Hence, we hypothesised that free cysteine in the media contributed to the generation of peptides with CysBP adducts.

To determine the role of extracellular cysteine in CysBP formation and peptide haptenation, we compared the abundance of the native, cysteinylated and CysBP‐modified versions of LLPPPPCPA isolated from C1R.A*02:01 cultured for the final 4 h in normal cysteine‐containing media ± BP (187 μM cysteine) to those cultured with BP in cysteine‐low media (11 μM cysteine) with and without cysteine supplementation. The LLPPPPCPA peptide was chosen based on it being the most abundant CysBP‐modified peptide in our original dataset. The peak area of peptides was normalised to spiked‐in iRT standards to account for peptide loading variation during sample acquisition on the mass spectrometer.

We first noted that in all conditions, with regular and low cysteine in the cell culture medium for the final 4 h, both native (Figure S7B) and cysteinylated versions of LLPPPPCPA (Figure S7C) were detected. Peak area quantification of LLPPPPCPA bearing CysBP (5.80E‐04) in the untreated sample (Figure S7D—Untreated) served as a background noise reference for comparing the levels of LLPPPPC(CysBP)PA observed in BP‐treated samples. Treatment of C1R.A*02:01 cells with BP in regular media induced the generation of LLPPPPC(CysBP)PA (Figure S7D–BP). On the other hand, the level of LLPPPPC(CysBP)PA eluted from BP‐treated C1R.A*02:01 cells cultured in a cysteine‐low environment was markedly reduced but still present (Figure S7D–BP, Cys‐low). Of note, the total reduced cysteine level in human plasma is reported to range between 8.3 and 10.7 μM [42], comparable to the cysteine‐low environment which facilitated Cys‐BP formation. Supplementation of cysteine into the cysteine‐low media led to comparable levels of LLPPPPC(CysBP)PA to those observed for cells cultured in regular media (Figure S7D–BP, Cys‐low, +Cys).

Next, we analysed the proteome of C1R.A*02:01 cells that were treated with 2 mM of BP for 4 or 48 h, with and without the use of a cysteine‐reducing agent during sample preparation. Only one peptide (2 spectra) was assigned a penicillin modification (which was at a lysine residue) after a 48 h treatment (Table S5, Appendix S4), suggesting that the majority of CysBP modifications were occurring on pHLA complexes already presented on the cell surface.

## 4 Discussion

Penicillin‐specific T cells are activated in an HLA‐restricted manner by the recognition of drug‐modified ligands. Through the use of a designer peptide to accommodate solvent‐exposed penicillin, Padovan et al. first demonstrated that penicillin‐specific T cells can react towards a BP‐modified epitope [17]. This was further replicated with either naturally presented or designer peptides accommodating either flucloxacillin, amoxicillin or BP [12, 16, 18, 20]. Importantly, studies describing the presentation of haptenated serum proteins for CD4^+^ T cell recognition [12] and CD8^+^ T cell recognition of naturally presented flucloxacillin‐modified peptides bound to HLA‐B*57:01 [15, 20], confirm the presence of haptenated ligands within the immunopeptidome. Although these immunogenicity studies incorporated a range of peptide sources—designer peptides capable of binding to HLA allotypes of interest [17, 18], peptides containing haptenation sites of proteins [12, 16, 19] or peptides directly isolated from HLA molecules [20], a common theme was the focus on direct penicillin modifications of lysine residues. This likely arises from the preponderance of lysine modifications identified in protein analyses, where the widespread use of reduction during sample preparation would abolish reduction‐sensitive cysteine‐drug adducts [9, 10, 11, 15, 32, 43, 44, 45].

In contrast, the lack of reducing agents in our immunopeptidome isolation workflow facilitated the identification of CysBP adducts. Of the BP modifications interrogated (BP at lysine, histidine, cysteine, arginine and CysBP at cysteine), CysBP modification of cysteine was the most common in our dataset, representing 42/55 peptides identified with unambiguous modification sites. As this discovery was in contrast to studies that focus on the simpler model of direct lysine haptenation [9, 10, 11, 12, 14, 18, 19, 20], we further verified CysBP through the synthesis and spectrum comparison of deliberately CysBP‐modified peptides. Importantly, the mechanism we describe does not appear to be restricted to BP and HLA‐A*02:01. Reanalysis of an immunopeptidome dataset for flucloxacillin modified HLA‐B*57:01 ligands [15] revealed a similar dominant contribution of CysFlux‐modified peptides.

Protein cysteinylation can occur to protect free thiols during the induction of oxidative stress in diseases such as sclerosis [46, 47]. Intracellular and extracellular proteins can also go through this thiol protection mechanism, and cysteinylation has been used as a marker for oxidative stress [47, 48, 49]. With regard to the immunopeptidome, cysteinylation is also a fairly common PTM that accounts for around 20% of modifications [50], is upregulated by flucloxacillin treatment [20] and can alter the immunogenicity of viral HLA ligands [51, 52]. Although cell culture media can contribute to the formation of CysBP in vitro (previously reported [34] and also detected in our cell culture supernatant, Figure S7, Appendix S2), it has also been postulated that free cysteine within serum might contribute to the formation of cysteinylated pHLA in vivo, akin to serum CysBP forming CysBP‐modified pHLA complexes [52]. This leads us to suggest two possible mechanisms for the formation of the CysBP thiol‐linked adducts at the cell surface: the direct interaction of BP with pre‐existing cysteinylated peptide ligands or the direct thiol attack of solvent‐accessible cysteine residues in HLA‐bound peptides by a preformed CysBP adduct generated either in the cell culture medium or in serum. Direct modification of surface peptide‐HLA molecules is further supported by the fact that the majority of these ligands were accommodated by peptides already naturally presented by HLA‐A*02:01 (Table S1, Appendix S4), and the lack of detection of CysBP adducts in the cellular proteome of BP‐treated cells (Table S5, Appendix S4).

A key finding of this study was the demonstration that the epitope of BP‐TCR is sensitive to reduction consistent with incorporation of CysBP modification. After showing that SKW3.BP‐TCR could be activated by peptides isolated from HLA‐A*02:01, we abrogated this reaction with the use of TCEP. This is the first report suggesting that penicillin‐specific T cells can be directed to a complex drug‐modified cysteine residue, implicating the relevance of CysBP in the onset of penicillin hypersensitivity reactions. The BP‐TCR also recognised epitopes formed from other β‐lactam molecules with adducts ranging in size from 216.25 Da (6‐aminopenicillanic acid) to 516.55 Da (piperacillin). Given that larger antibiotics like flucloxacillin (453.06 Da) are thought to be too large to be accommodated within the peptide‐binding cleft [20], it is likely that BP‐TCR recognises a solvent‐exposed, reduction‐sensitive motif that is shared between penicillin family members. However, it is important to note that this study reports on a single BP‐specific TCR that is cross‐reactive to other penicillins, which might not represent the reactivity of the entire repertoire of penicillin‐specific T cells. For instance, it has been reported that piperacillin‐specific T cells do not cross‐react to BP and amoxicillin [53].

Although this study was focused on HLA‐A*02:01, it should be recognised that there are other HLA allotypes implicated in adverse reactions to penicillins [4]. For instance, HLA‐B*57:01 has been associated with flucloxacillin‐induced liver injury [54, 55], while HLA‐B*55:01 was recently reported as associated with self‐reported penicillin allergy [56]. Furthermore, not all individuals carrying predisposing HLA allotypes develop an allergic reaction, and other factors such as an individual's state of health (e.g., cystic fibrosis [57]) and T_reg_ dysregulation [58] have also been suggested to play a role in the pathogenesis of penicillin‐induced DHRs.

In conclusion, we identified a high frequency of CysBP‐modified HLA ligands induced by BP treatment that provide an explanation for the reduction‐sensitive epitope of a dominant BP‐TCR isolated from a penicillin‐hypersensitive patient. Furthermore, we demonstrated that extracellular cysteine can contribute to the formation of CysBP‐modified peptides. Our findings generate impetus for future investigation of the influence of free cysteine levels on the formation of drug‐modified ligands within the immunopeptidome, to in turn provide insight into the conditions that may predispose individuals towards activation of drug‐specific T cells.

## Author Contributions

S.J.R.G. conducted peptide elution, immunopeptidome analyses, penicillin modification of peptides and proteomic experiments. S.J.R.G., H.H.W.L., K.P., N.A.M. and P.T.I. conducted cellular and molecular experiments. K.E.S. wrote the PenicillinFinder script. S.J.R.G. and M.D. established quantitative proteomics parameters and acquisition. K.D., R.P. and R.E.O. recruited and collected the patient sample used. S.J.R.G., A.W.P., N.A.M. and P.T.I. designed the study and experiments and drafted the manuscript. All authors critically reviewed the manuscript.

## Conflicts of Interest

A.W.P. is a scientific advisor for Bioinformatics Solutions Inc. (Canada) and Grey Wolf Therapeutics (United Kingdom), a shareholder and scientific advisor for Evaxion Biotech (Denmark), and a co‐founder of Resseptor Therapeutics (Australia). S.J.R.G. is currently an employee of Resseptor Therapeutics. These organisations had no role in the design of the study; in the collection, analyses or interpretation of data; in the writing of the manuscript; or in the decision to publish the results.

## Supporting information


**Appendix S1:** Supporting Information.

**Appendix S2:** Supporting Information.

**Appendix S3:** Supporting Information.

**Appendix S4:** Supporting Information.

## Data Availability

All mass spectrometry data have been deposited to the ProteomeXchange via the PRIDE partner repository with the identifier PXD057177 and PXD065200.
