# Supplementary material for: HLA‐A*02:01 Presents Penicillin‐Modified Cysteinylated Peptides for T Cell Recognition
Source: Allergy. 2025 Sep 4;80(11):3165–77. doi: 10.1111/all.70025 (PMC12590334; doi:10.1111/all.70025)
Supplement: Supplementary file 1 — Appendix S1: Supporting Information. [file ALL-80-3165-s004.pdf]

Penicillin-modified peptide spectra analysis

- Green boxes within mass spectra mark penicillin-specific diagnostic ions.
  - m/z 160.04: Thiazolidine ring
  - m/z 217.06: Penam core
  - m/z 335.11: Benzylpenicillin (BP)

Peptide	Length	Modification(s)	-10lgP	Scan	m/z	charge	RT(min)
AINDECYQV	9	BenzylpenicillinCC(C)@6;	12.96	25364	754.282	2	75.7618

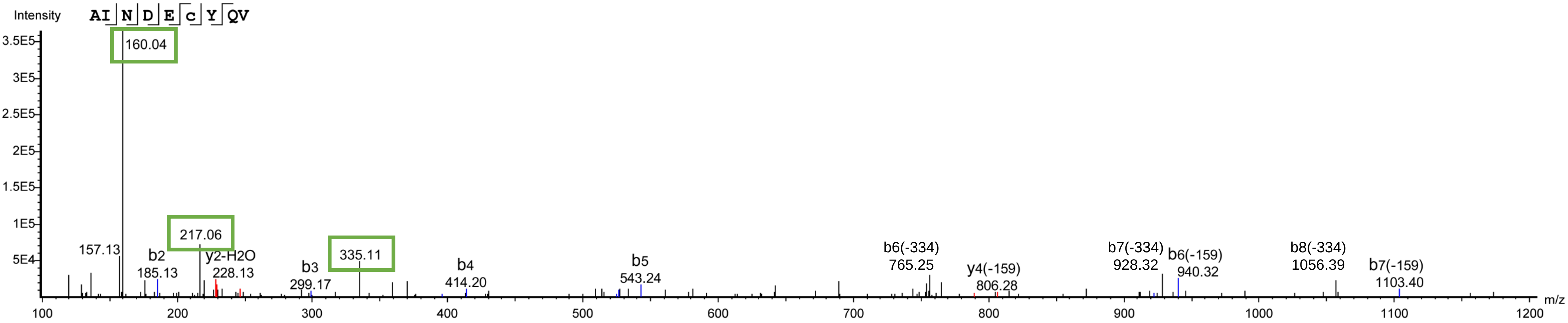

Peptide	Length	Modification(s)	-10lgP	Scan	m/z	charge	RT(min)
ALCDFGHAV	9	BenzylpenicillinCC(C)@3;	22.35	25757	693.269	2	72.4996

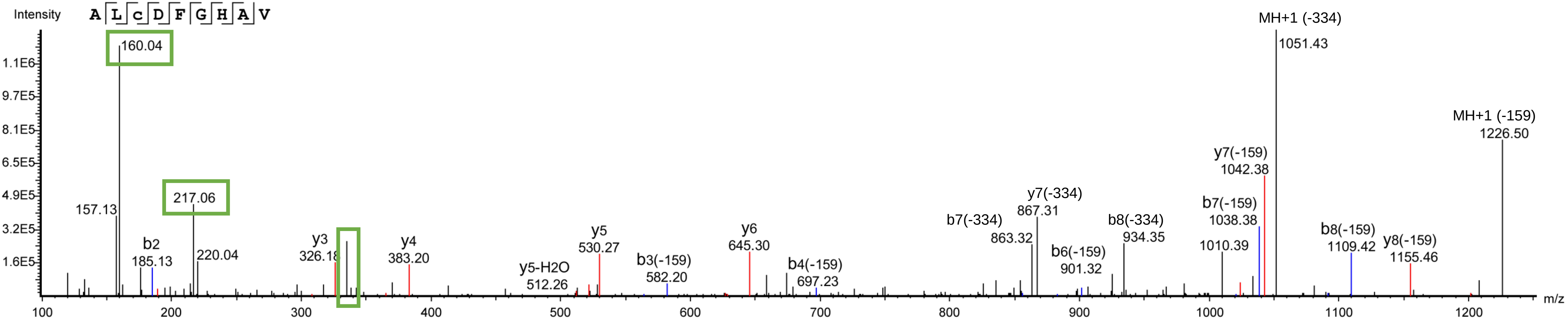

Support for assignment: Presence of b3 with partial adduct.
Lack of y ions with partial or full adduct before C3.

Peptide	Length	Modification(s)	-10lgP	Scan	m/z	charge	RT(min)
ALCEENMRGV	10	BenzylpenicillinCC(C)@3;Oxidation(M)@7;	11.64	26373	795.81	2	54.5384

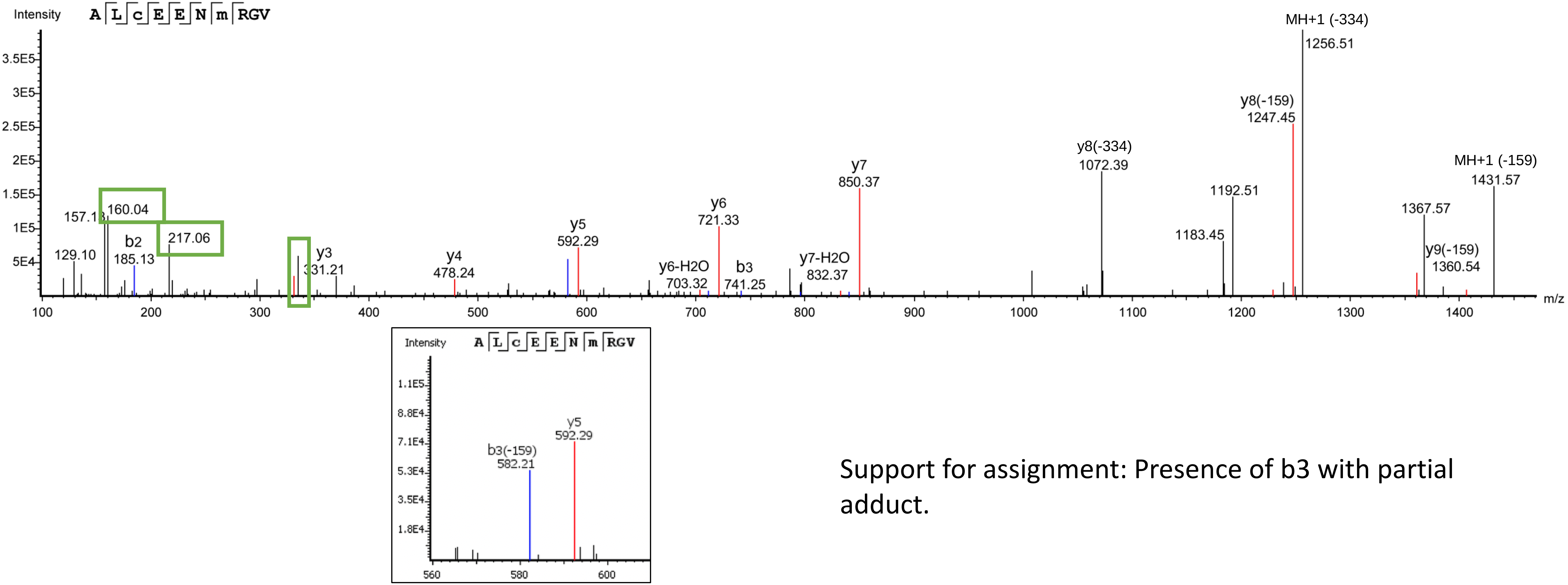

Support for assignment: Presence of b3 with partial adduct.

Peptide	Length	Modification(s)	-10lgP	Scan	m/z	charge	RT(min)
ALCPHLKTL	9	BenzylpenicillinCC(C)@3;	19.98	18989	483.564	3	59.0129

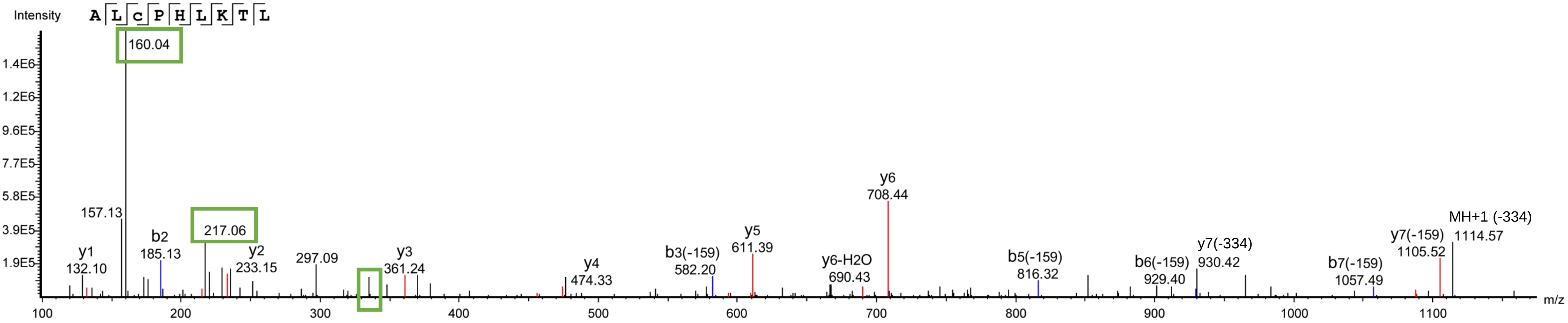

Support for assignment: Presence of b3 with partial adduct.
Lack of y3 and y5 with partial or full adduct.

Peptide	Length	Modification(s)	-10lgP	Scan	m/z	charge	RT(min)
ALCPQVINA	9	BenzylpenicillinCC(C)@3;	13.22	26163	691.303	2	76.6661

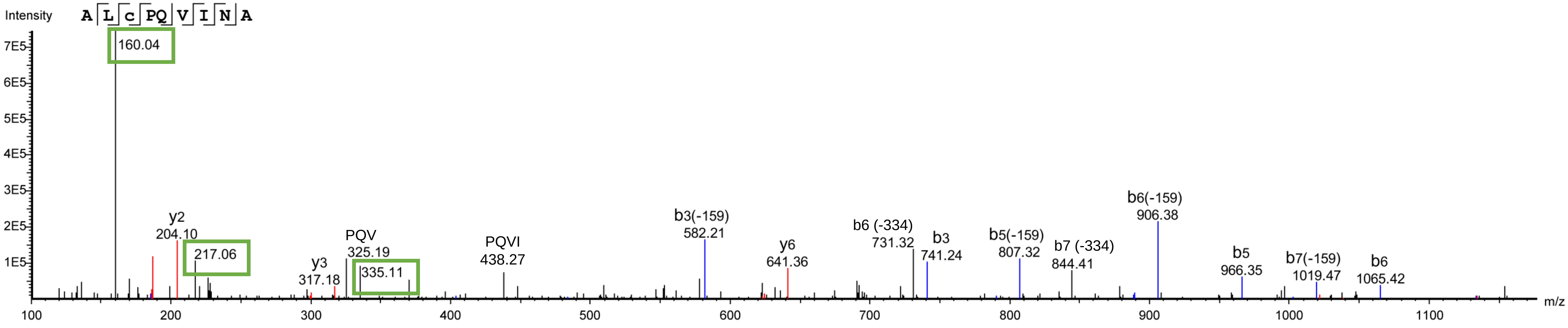

Ambiguous

Peptide	Length	Modification(s)	-10lgP	Scan	m/z	charge	RT(min)
ALFKCKFEEA	10	Cysteinyl(C)@5;BenzylpenicillinK(K)@6;	19.22	19418	546.907	3	60.1125

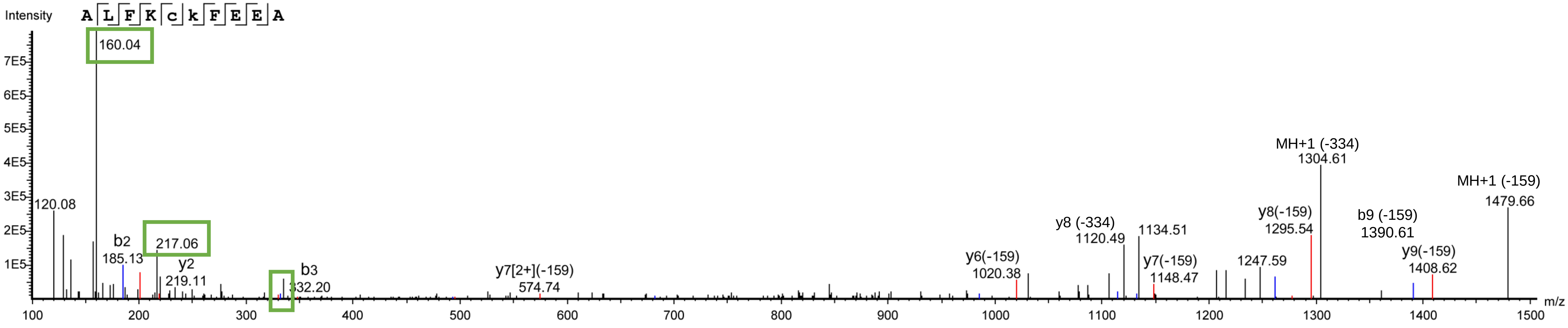

Ambiguous. Lack of y5 with partial or full adduct. Lack of b4 and b5 with partial or full adduct.

Peptide	Length	Modification(s)	-10lgP	Scan	m/z	charge	RT(min)
ALIGNLVKC	9	BenzylpenicillinCC(C)@9;	21.6	24477	692.328	2	73.5641

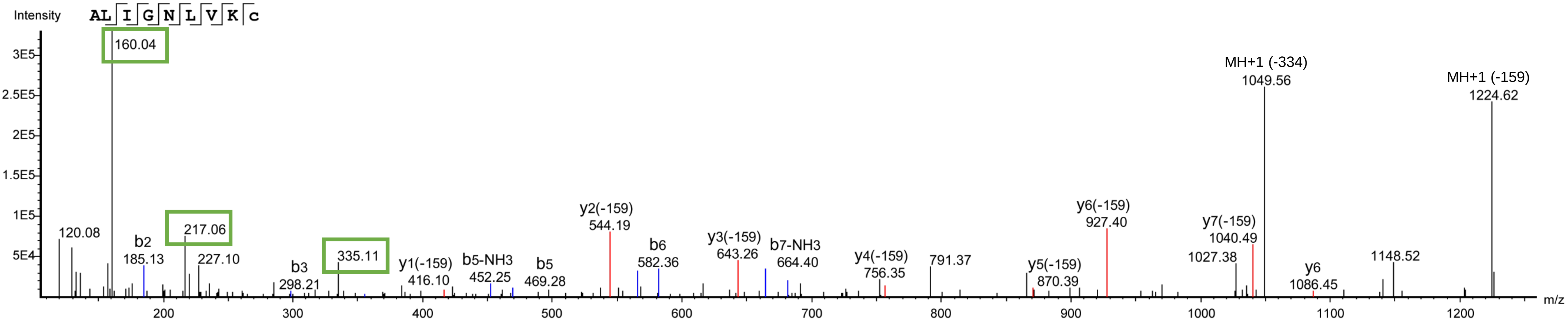

Support for assignment: Presence of y1 with partial adduct.
Lack of b8 with partial or full adduct.

Peptide	Length	Modification(s)	-10lgP	Scan	m/z	charge	RT(min)
ALLSSLARC	9	BenzylpenicillinCC(C)@9;	13.95	23913	462.88	3	70.1048

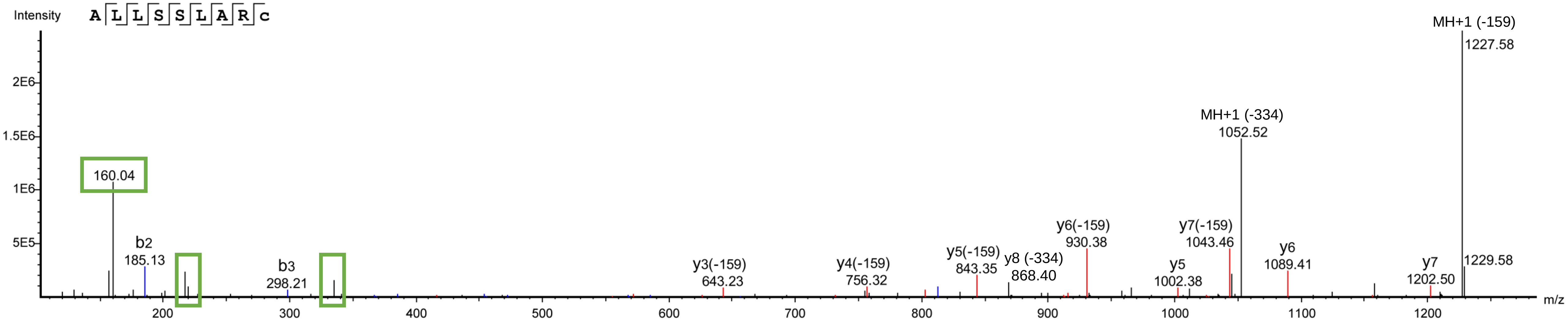

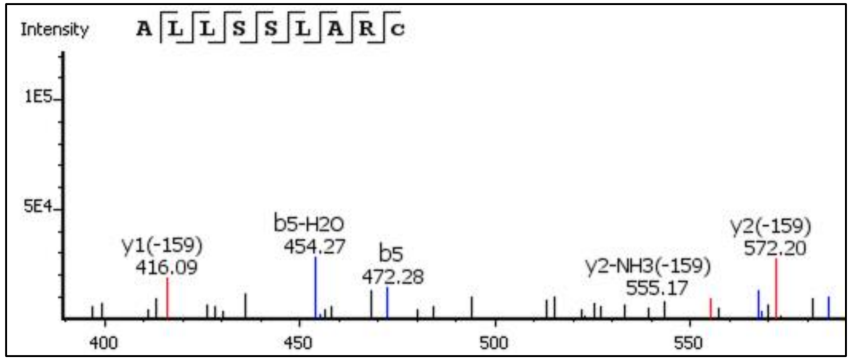

Support for assignment: Presence of y1 with partial adduct.

Ambiguous

Peptide	Length	Modification(s)	-10lgP	Scan	m/z	charge	RT(min)
ATADYICKV	9	BenzylpenicillinCC(C)@7;	20.8	22167	718.799	2	66.7622

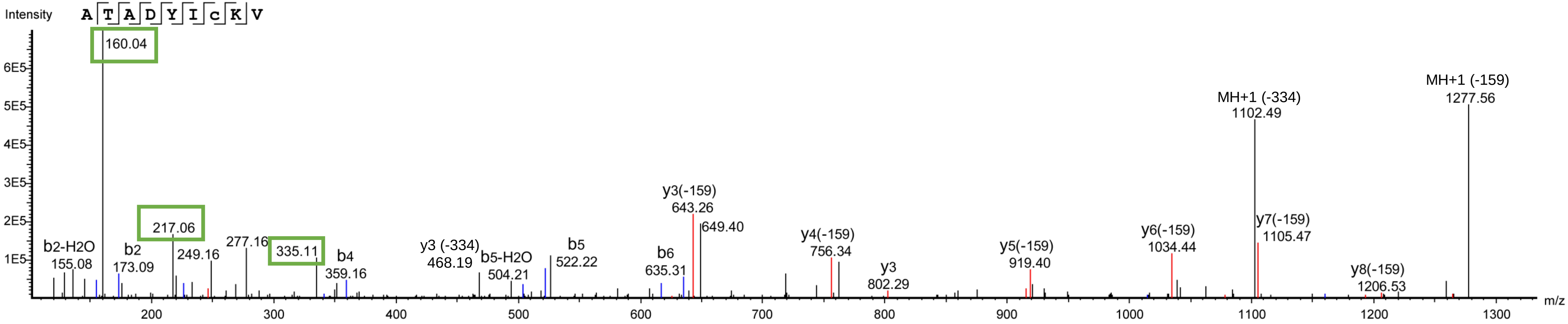

Ambiguous. Lack of y2 ions with partial or full adduct. Lack of b7 ion with partial or full adduct

Ambiguous

Peptide	Length	Modification(s)	-10lgP	Scan	m/z	charge	RT(min)
ATADYICKV	9	Cysteinyl(C)@7;BenzylpenicillinK(K)@8;	20.66	20490	718.799	2	63.6826

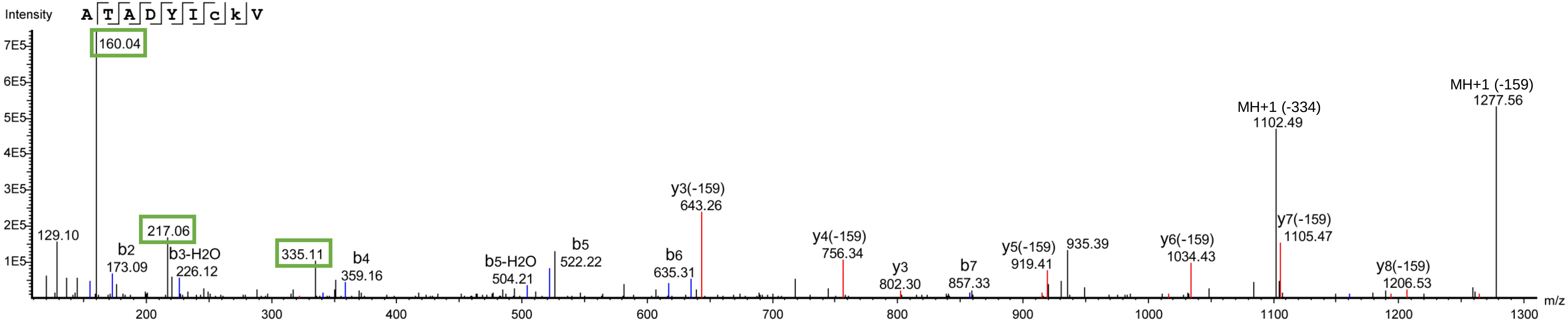

Ambiguous. Lack of y2 ions with partial or full adduct. Lack of b7 ion with partial or full adduct

Peptide	Length	Modification(s)	-10lgP	Scan	m/z	charge	RT(min)
CFFASLFSV	9	BenzylpenicillinCC(C)@1;	14.22	25951	737.299	2	77.206

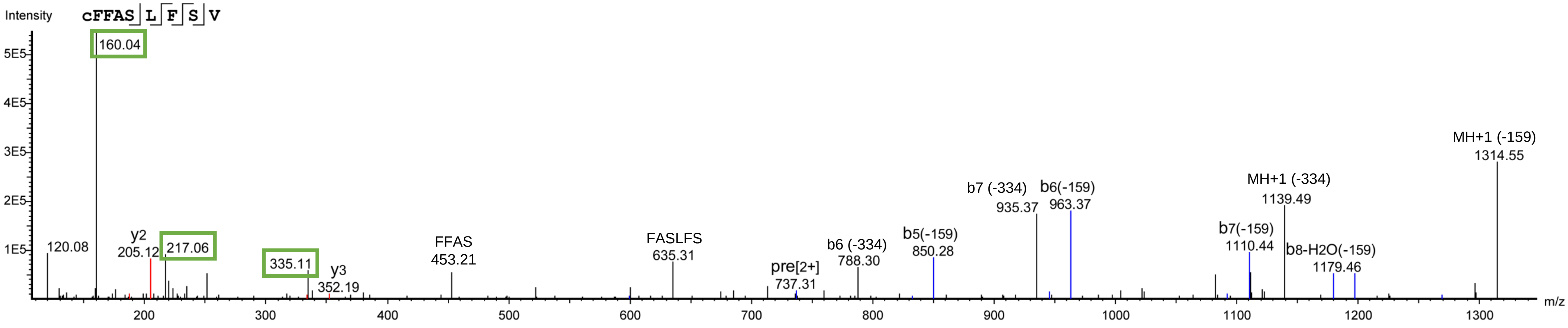

Peptide	Length	Modification(s)	-10lgP	Scan	m/z	charge	RT(min)
CIFEGEIAKA	10	BenzylpenicillinCC(C)@1;	22.76	24754	767.324	2	73.9308

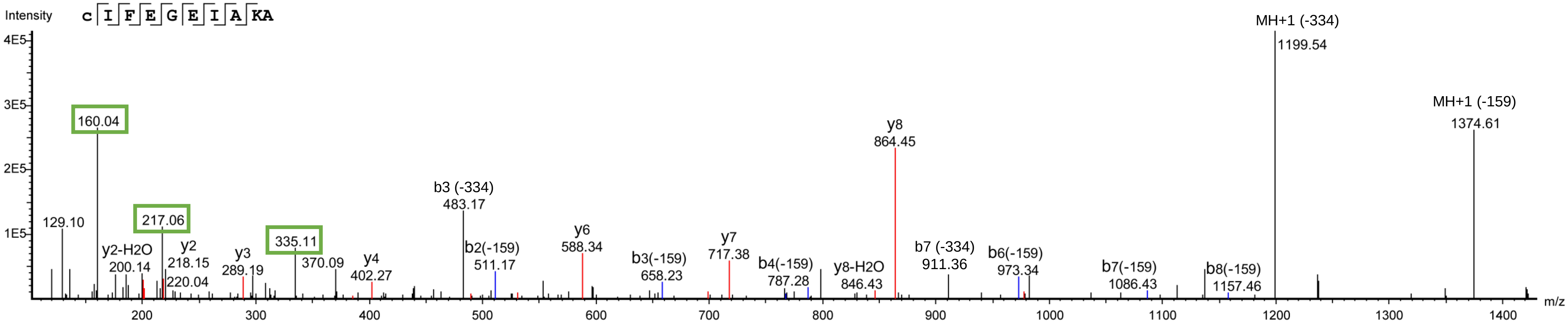

Support for assignment: Presence of b2, b3, b4, b6, b7, and b8 ions containing either partial or full adduct before K9. Lack of y ions containing partial or full adduct.

Peptide	Length	Modification(s)	-10lgP	Scan	m/z	charge	RT(min)
CIIDKEVSL	9	BenzylpenicillinCC(C)@1;	16.02	25651	736.826	2	74.9089

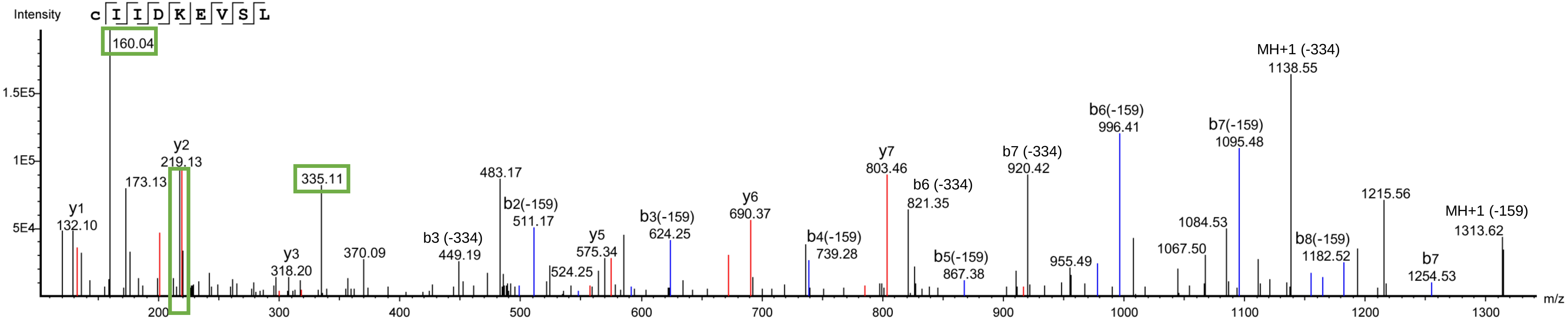

Support for assignment: Lack of y5 with partial or full adduct.
Presence of b ion series with partial adduct before K.

Peptide	Length	Modification(s)	-10lgP	Scan	m/z	charge	RT(min)
CLDEKEFQV	9	BenzylpenicillinCC(C)@1;	23.54	24621	782.312	2	73.6039

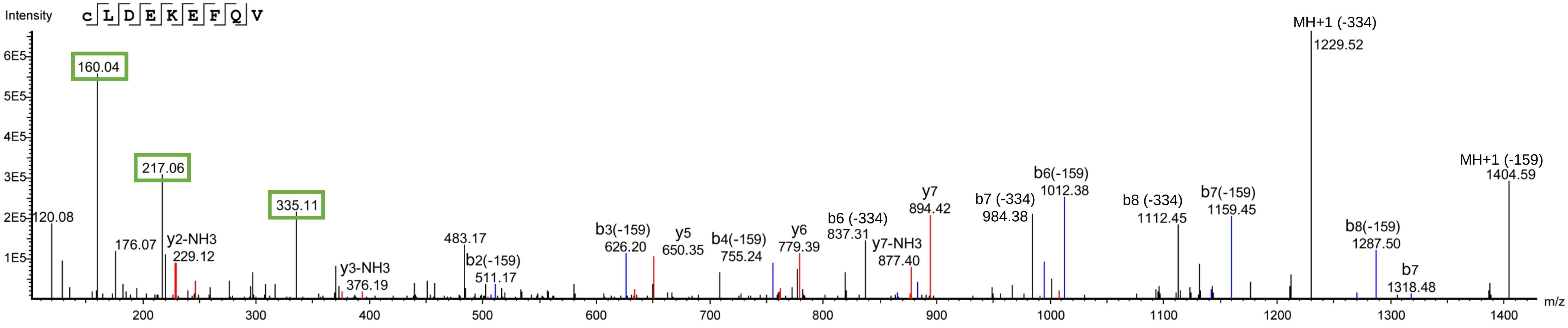

Support for assignment: Presence of b2, b3, and b4 ions containing partial adduct before K5. Lack of y ions containing partial or full adduct.

Peptide	Length	Modification(s)	-10lgP	Scan	m/z	charge	RT(min)
CLDIHNMSV	9	BenzylpenicillinCC(C)@1;	14.23	25822	495.529	3	75.0359

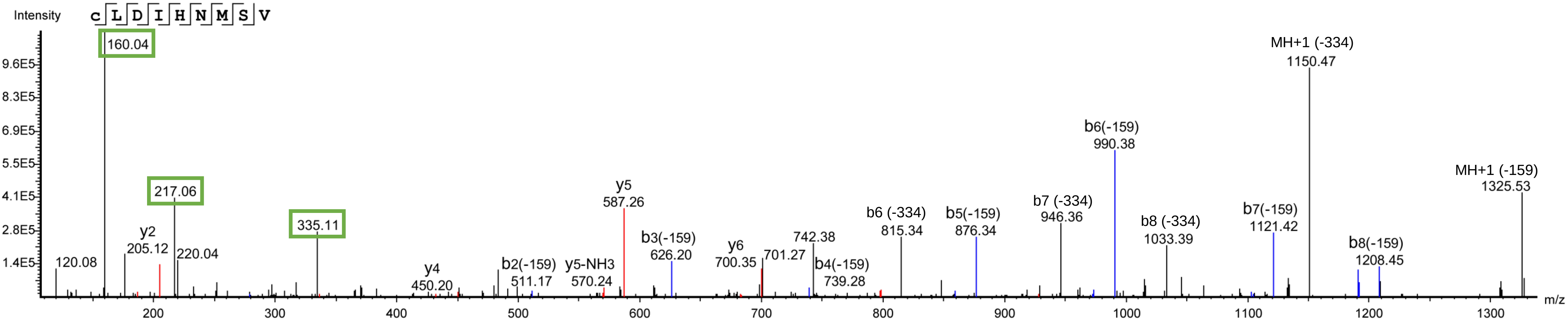

Support for assignment: Presence of b2, b3, and b4 ions containing partial adduct before H5. Lack of y ions containing partial or full adduct.

Peptide	Length	Modification(s)	-10lgP	Scan	m/z	charge	RT(min)
CLDIHNMSV	9	BenzylpenicillinCC(C)@1;Oxidation(M)@7;	14.42	21605	750.787	2	65.7946

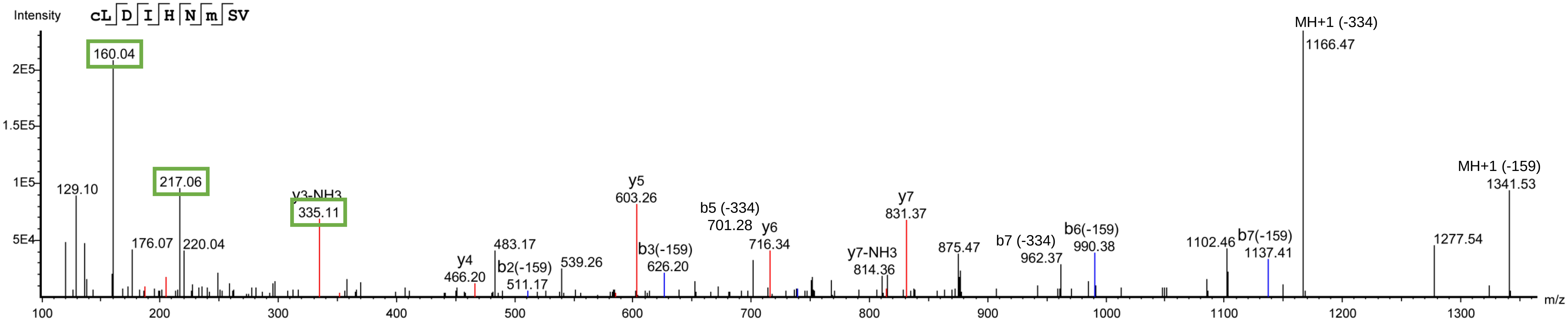

Support for assignment: Presence of b2 and b3 ions containing partial adduct before H5. Lack of y ions containing partial or full adduct.

Peptide	Length	Modification(s)	-10lgP	Scan	m/z	charge	RT(min)
CLVKNLEAV	9	BenzylpenicillinCC(C)@1;	18.48	24919	721.33	2	74.3361

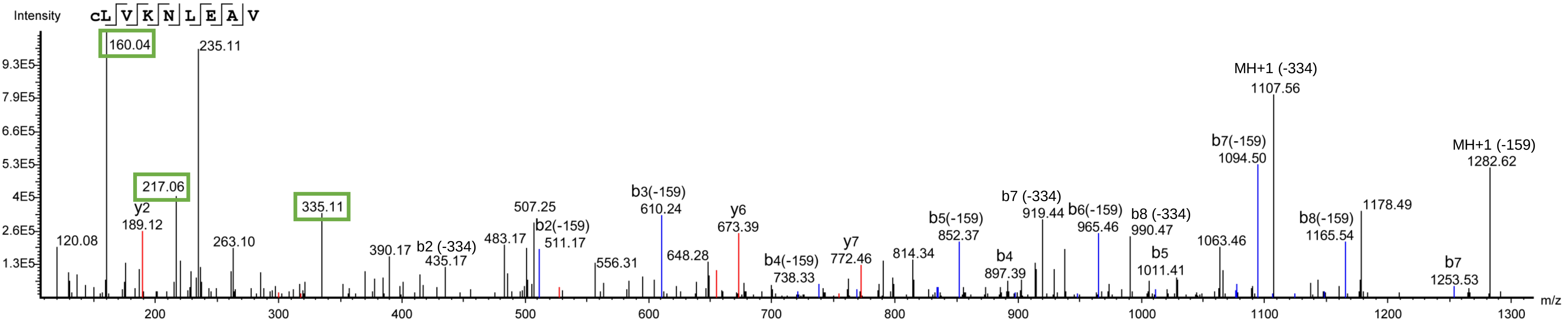

Support for assignment: Presence of b2 and b3 ions containing partial adduct before K4. Lack of y6 and y7 containing partial or full adduct.

Peptide	Length	Modification(s)	-10lgP	Scan	m/z	charge	RT(min)
CLYGNVEKV	9	BenzylpenicillinCC(C)@1;	28.64	22200	739.311	2	67.9293

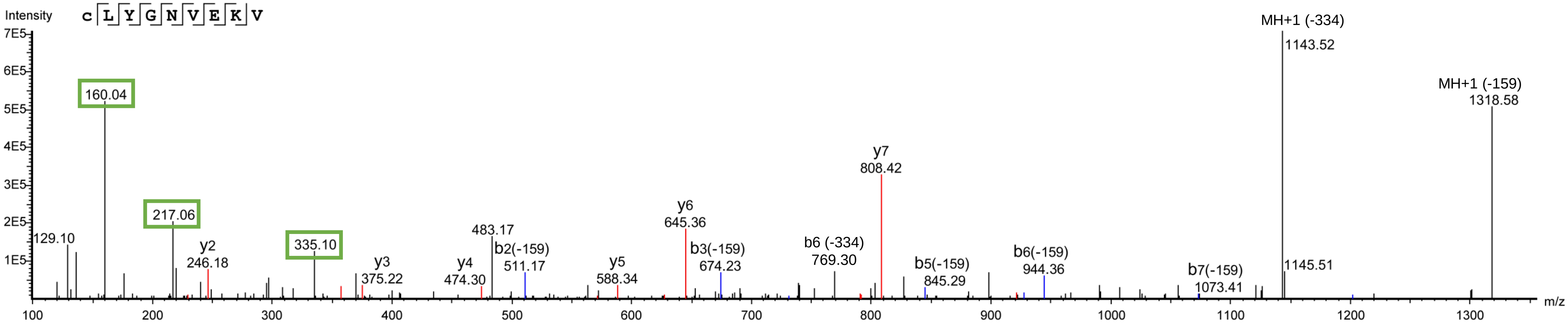

Support for assignment: Presence of b2, b3, b5, b6, and b7 ion series with partial adduct before K8. Lack of y2 ion with partial or full adduct.

Peptide	Length	Modification(s)	-10lgP	Scan	m/z	charge	RT(min)
CLYPEVHYL	9	BenzylpenicillinCC(C)@1;	31.39	25655	795.33	2	76.4799

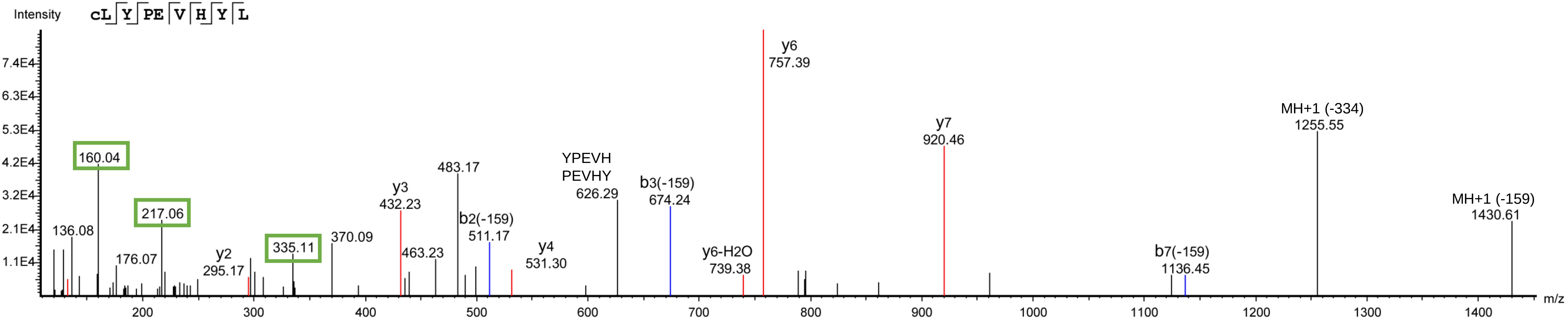

Support for assignment: Presence of b2 and b3 ions with partial adduct before H7.

Peptide	Length	Modification(s)	-10lgP	Scan	m/z	charge	RT(min)
CWDHRPVQI	9	BenzylpenicillinCC(C)@1;	12.79	20444	803.836	2	62.2799

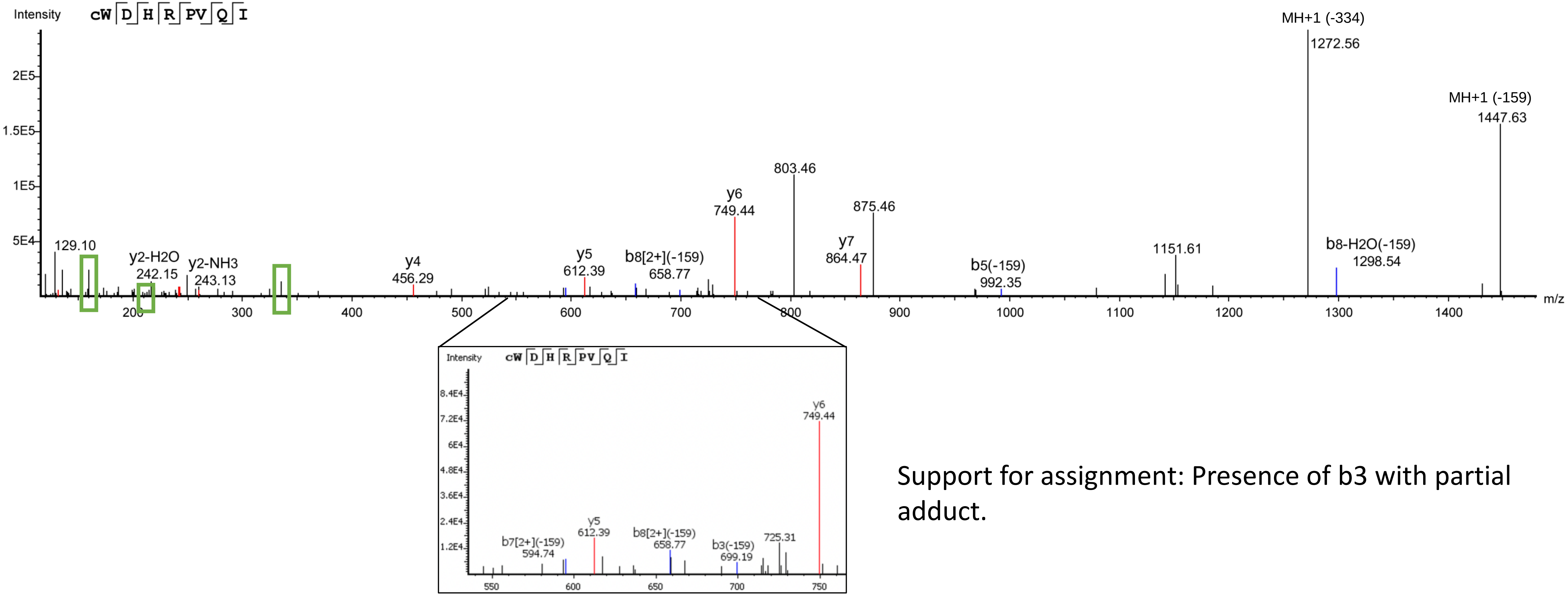

Support for assignment: Presence of b3 with partial adduct.

Incorrect

Peptide	Length	Modification(s)	-10lgP	Scan	m/z	charge	RT(min)
ESCFAVGPII	10	BenzylpenicillinCC(C)@3;	13.98	24308	744.818	2	73.119

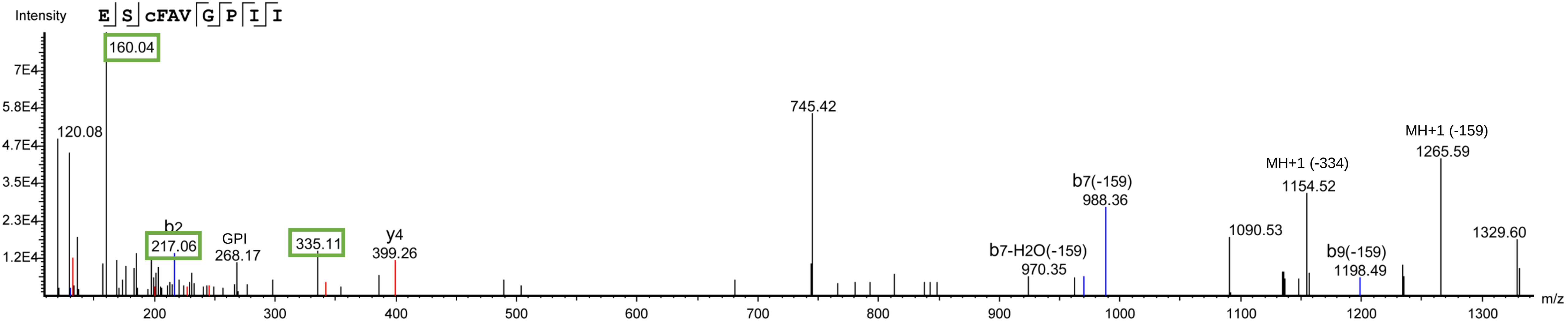

Incorrect. Diagnostic ion used assigned b2 ion.

Peptide	Length	Modification(s)	-10lgP	Scan	m/z	charge	RT(min)
FCFDGTKAV	9	BenzylpenicillinCC(C)@2;	16.76	25467	720.786	2	75.6693

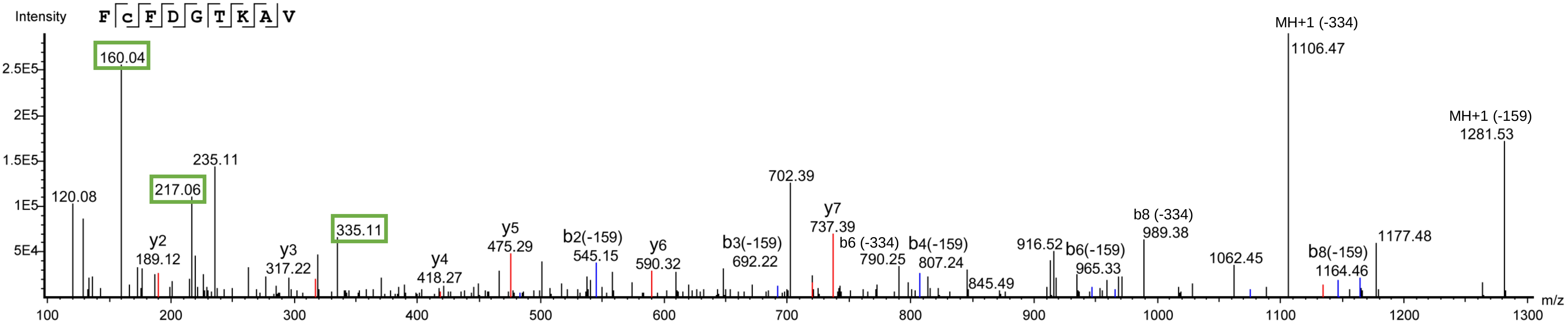

Support for assignment: Presence of b2, b3, b4, and b6 ion series with partial adduct before K7.

Peptide	Length	Modification(s)	-10lgP	Scan	m/z	charge	RT(min)
FIADHCPTL	9	BenzylpenicillinCC(C)@6;	19.97	23976	735.304	2	71.4551

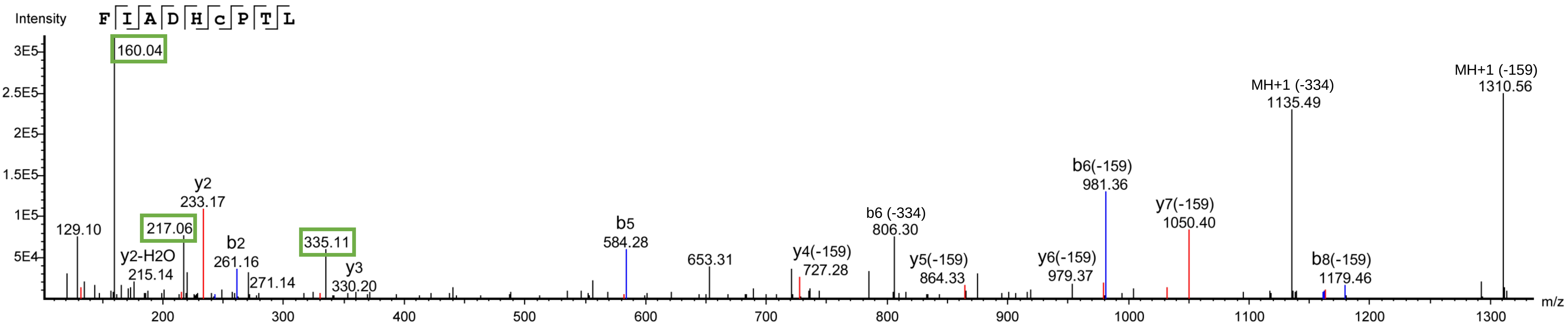

Support for assignment: Presence of y4 with partial adduct.
Lack of b5 with partial or full adduct.

Peptide	Length	Modification(s)	-10lgP	Scan	m/z	charge	RT(min)
FLLDKKIGV	9	BenzylpenicillinK(K)@6;	12.63	25140	683.874	2	75.1677

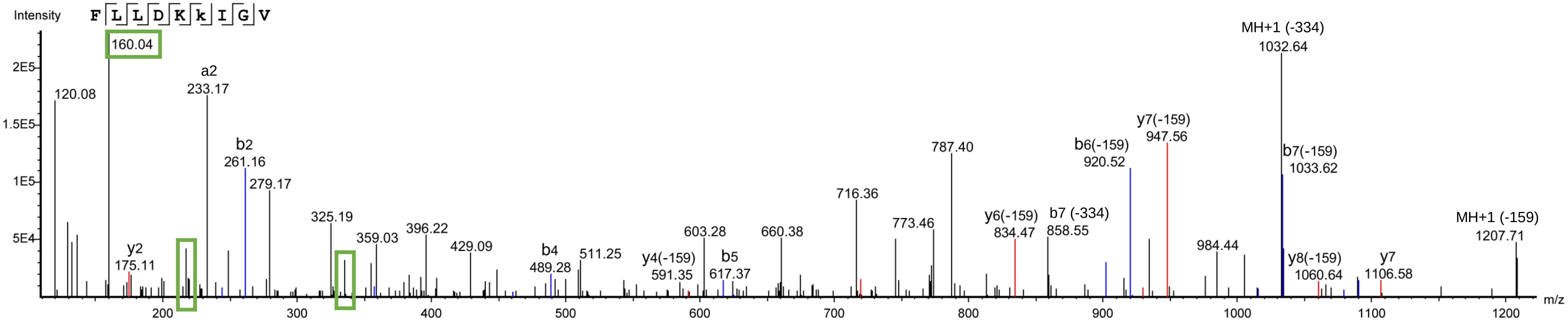

Support for assignment: Lack of b5 with partial and full adduct.
Presence of y4 with partial adduct.

Peptide	Length	Modification(s)	-10lgP	Scan	m/z	charge	RT(min)
FLQENPSCL	9	BenzylpenicillinCC(C)@8;	12.23	26026	752.301	2	76.9892

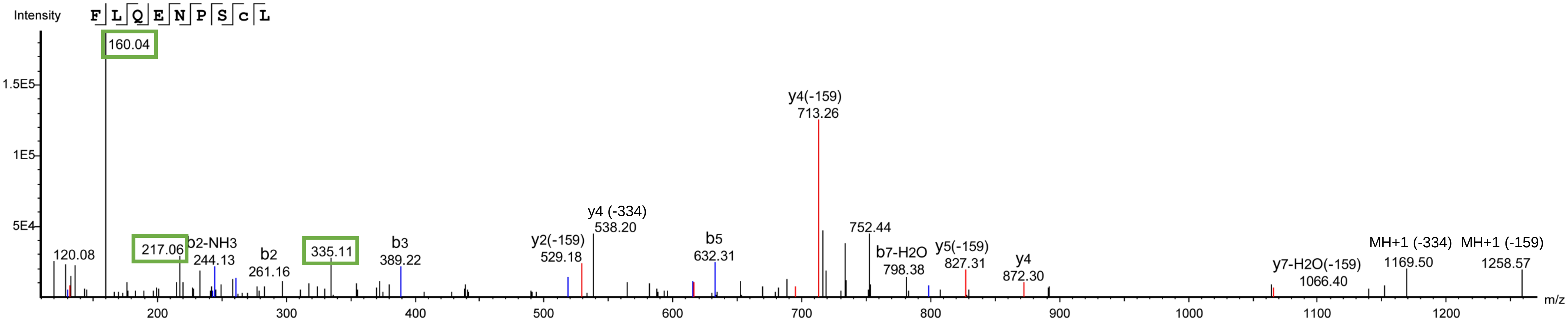

Peptide	Length	Modification(s)	-10lgP	Scan	m/z	charge	RT(min)
FMDESTQCF	9	Oxidation(M)@2;BenzylpenicillinCC(C)@8;	21.39	25648	788.76	2	76.1243

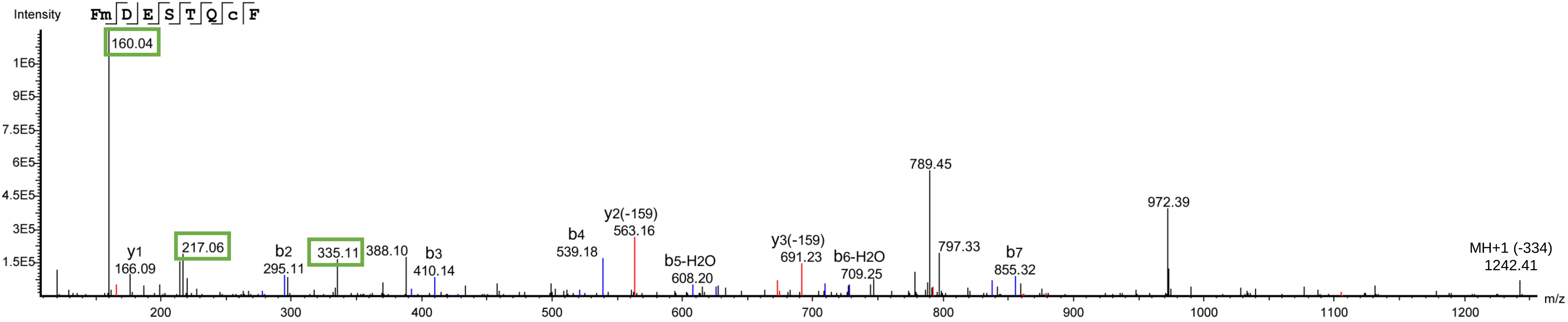

Peptide	Length	Modification(s)	-10lgP	Scan	m/z	charge	RT(min)
HFDDTVVCL	9	BenzylpenicillinC(C)@8;	17.52	25715	691.793	2	75.7733

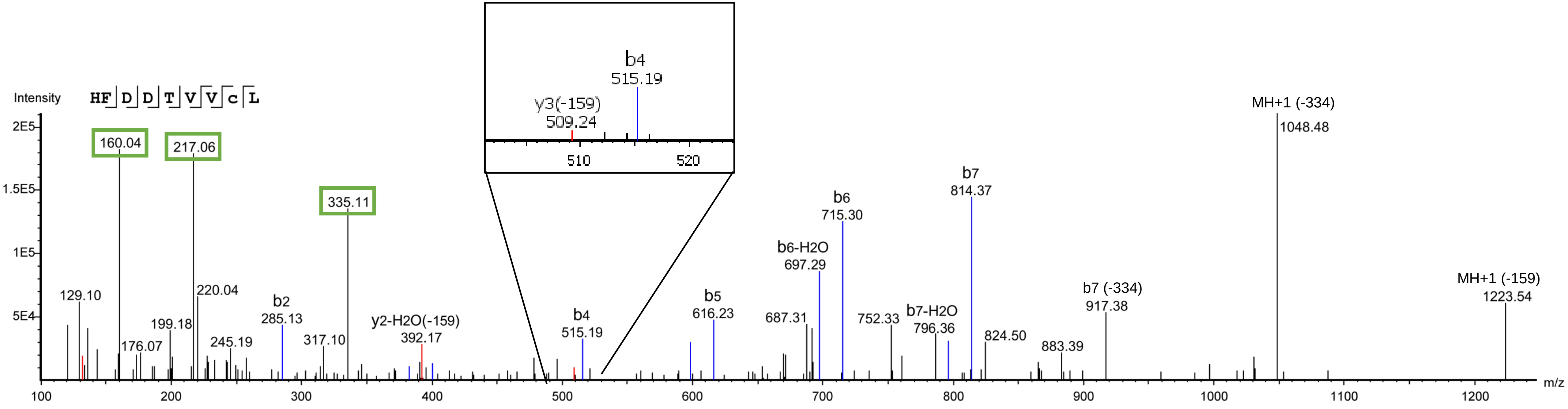

Support for assignment: Presence of y2-H2O and y3 with partial adduct. Lack of b ions with partial or full adduct before C8.

Peptide	Length	Modification(s)	-10lgP	Scan	m/z	charge	RT(min)
HFDDTVVCL	9	BenzylpenicillinCC(C)@8;	22.69	25923	751.296	2	77.1342

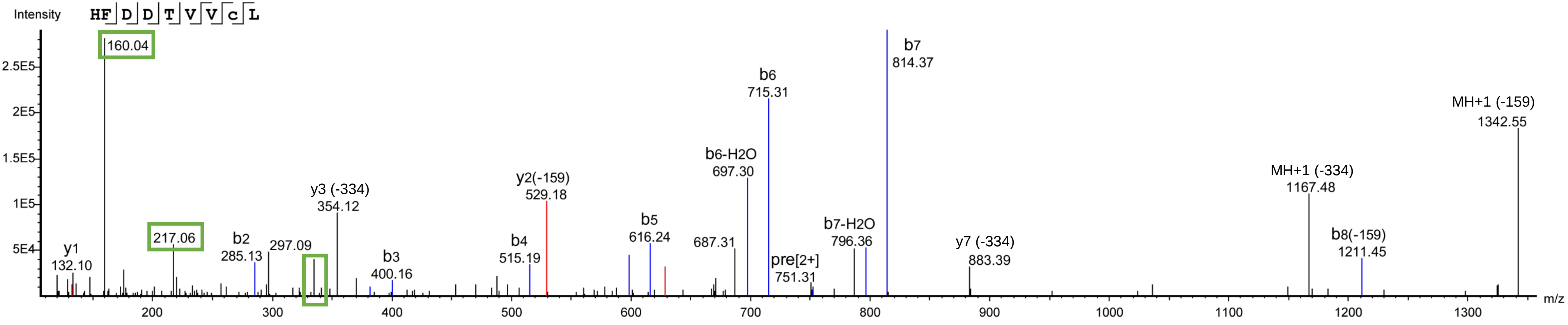

Support for assignment: Presence of y2 with partial adduct. Lack of b ions with partial or full adduct before C8.

Peptide	Length	Modification(s)	-10lgP	Scan	m/z	charge	RT(min)
HLASVYCTV	9	BenzylpenicillinCC(C)@7;	14.6	22291	723.301	2	67.0804

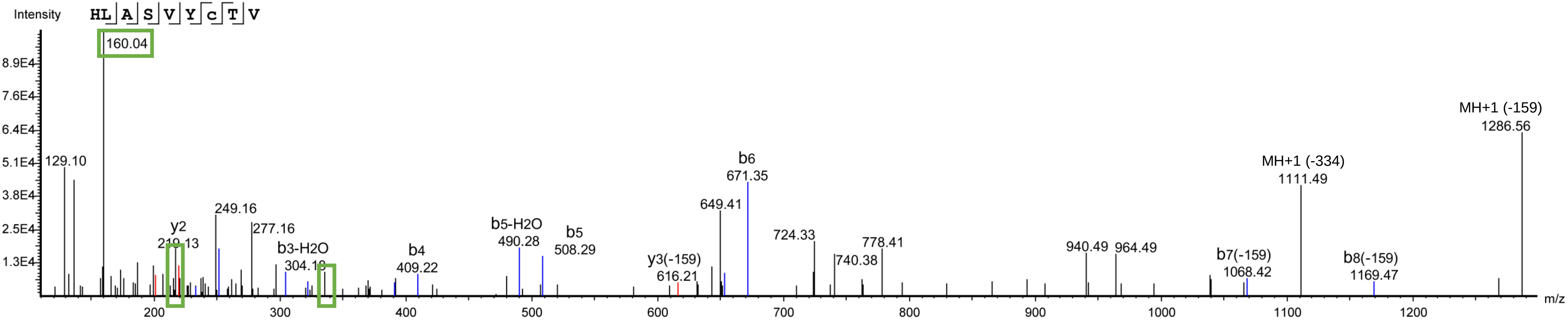

Support for assignment: Presence of y3 with partial adduct.
Lack of b ions with partial or full adduct before C7.

Peptide	Length	Modification(s)	-10lgP	Scan	m/z	charge	RT(min)
HLLEVVNQC	9	BenzylpenicillinCC(C)@9;	22.12	22792	754.322	2	68.3807

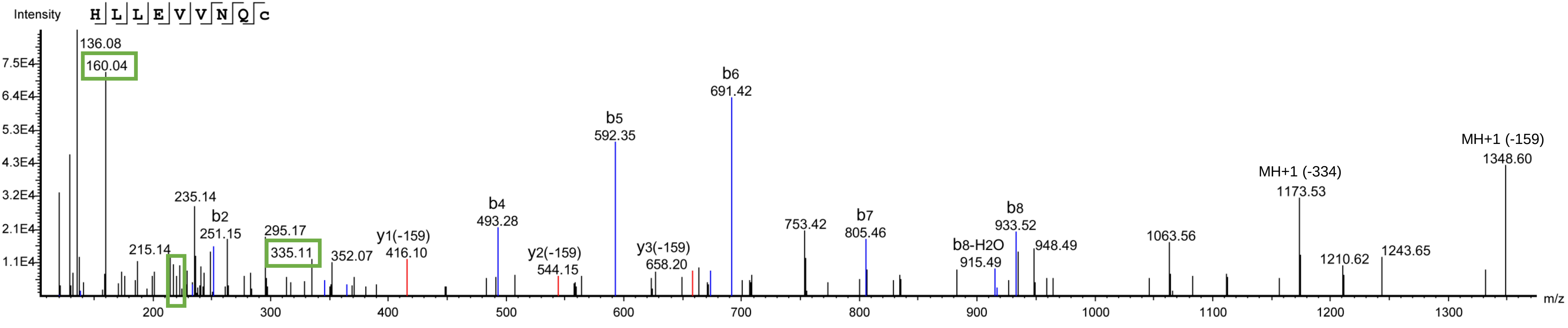

Support for assignment: Presence of y1 with partial adduct.
Lack of b ions containing partial or full adduct before C9.

Peptide	Length	Modification(s)	-10lgP	Scan	m/z	charge	RT(min)
IIDGKIFCV	9	BenzylpenicillinC(C)@8;	16.19	25490	671.336	2	75.3814

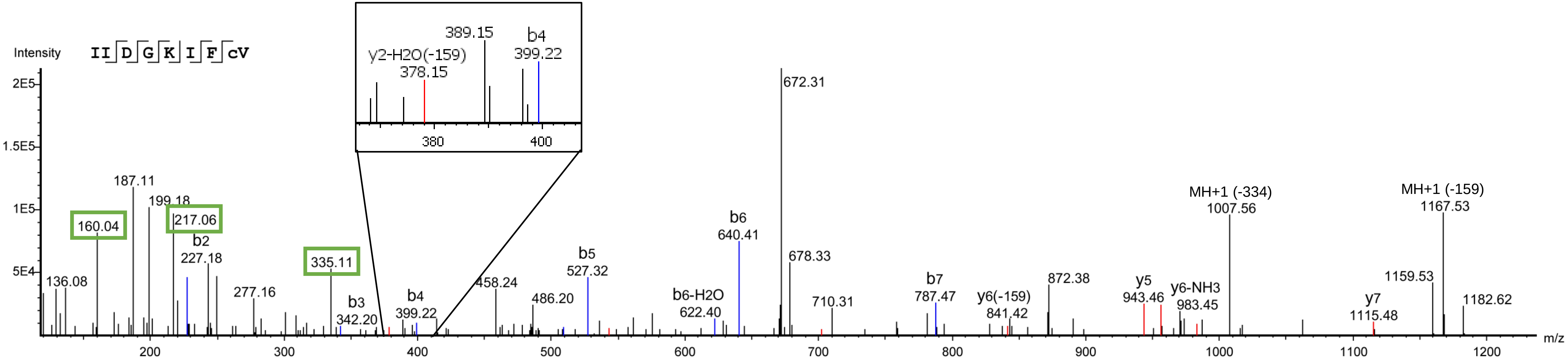

Support for assignment: Presence of y2-H2O with partial adduct. Lack of b ions with partial or full adduct before C8.

Peptide	Length	Modification(s)	-10lgP	Scan	m/z	charge	RT(min)
IIDGKIFCV	9	BenzylpenicillinCC(C)@8;	25.33	25739	730.838	2	76.6797

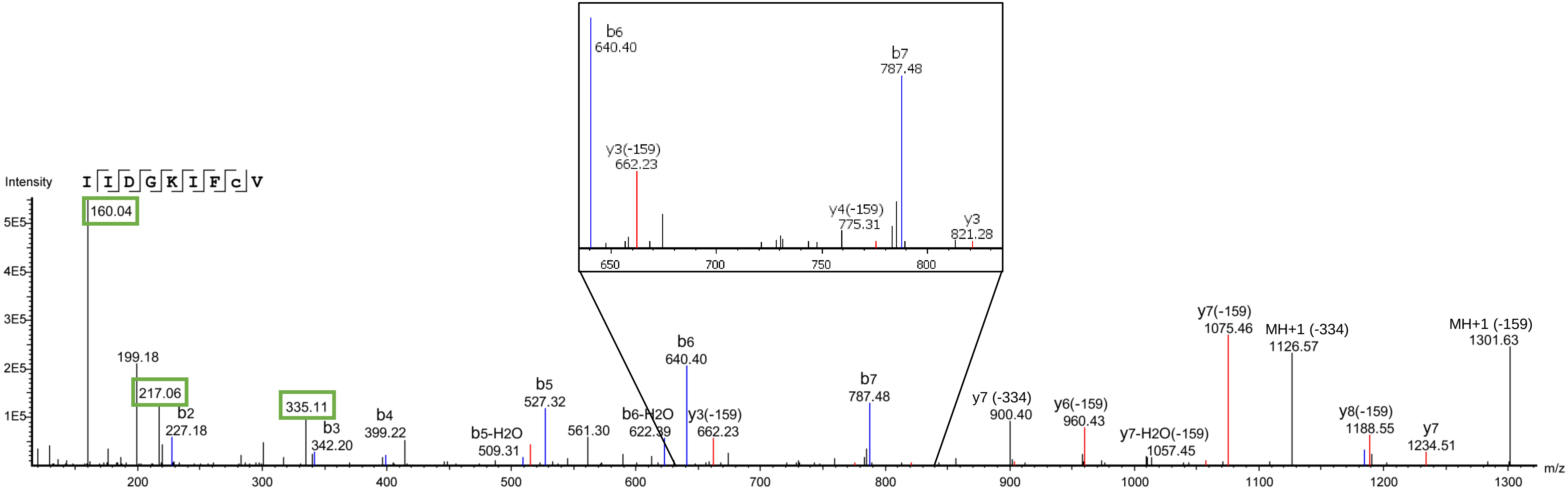

Support for assignment: Lack of b5 with partial or full adduct.
Presence of y3 with partial and full adduct.

Peptide	Length	Modification(s)	-10lgP	Scan	m/z	charge	RT(min)
ILANNCPAL	9	BenzylpenicillinCC(C)@6;	21.5	26073	691.302	2	77.0988

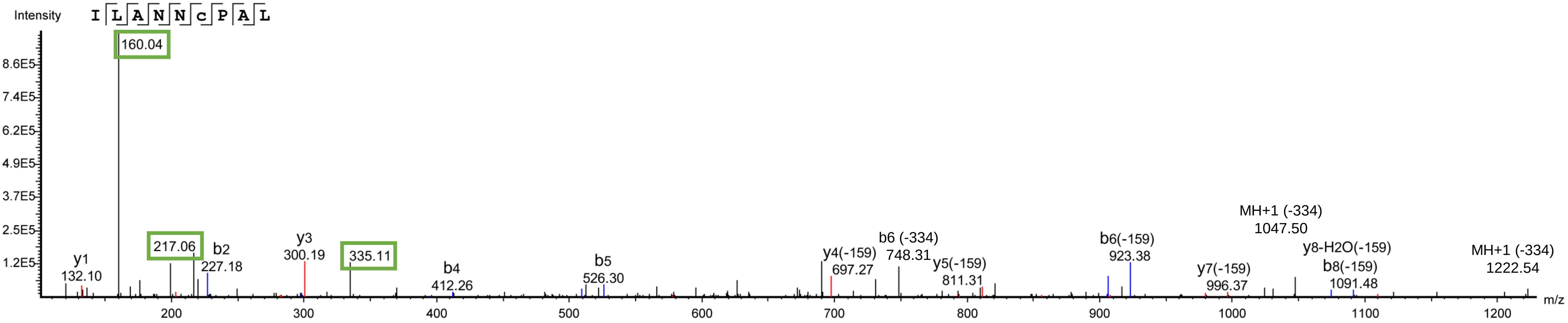

Peptide	Length	Modification(s)	-10lgP	Scan	m/z	charge	RT(min)
ILDKKVEKV	9	BenzylpenicillinK(K)@4;	23.73	13972	703.392	2	46.4486

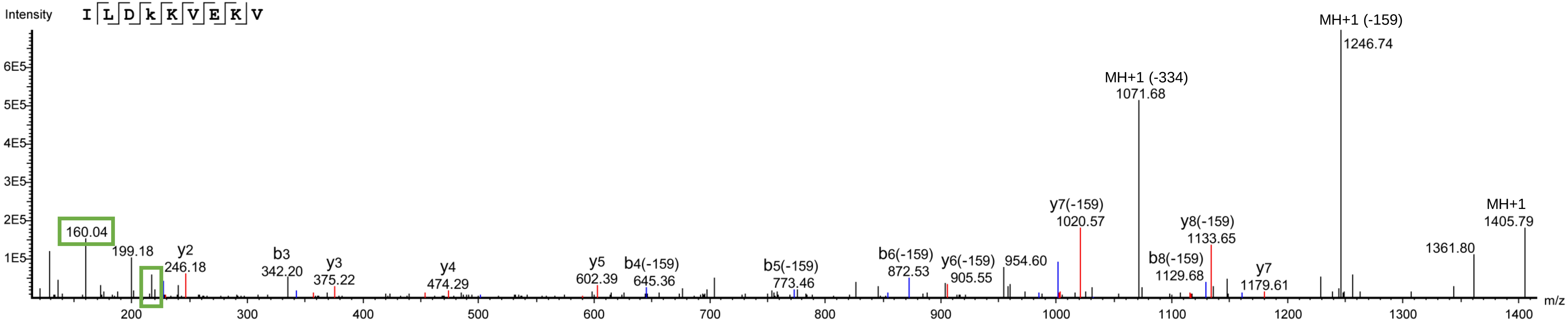

Support for assignment: Presence of b4 with partial adduct.

Peptide	Length	Modification(s)	-10lgP	Scan	m/z	charge	RT(min)
ILDKKVEKV	9	BenzylpenicillinK(K)@5;	12.52	14977	469.264	3	48.9969

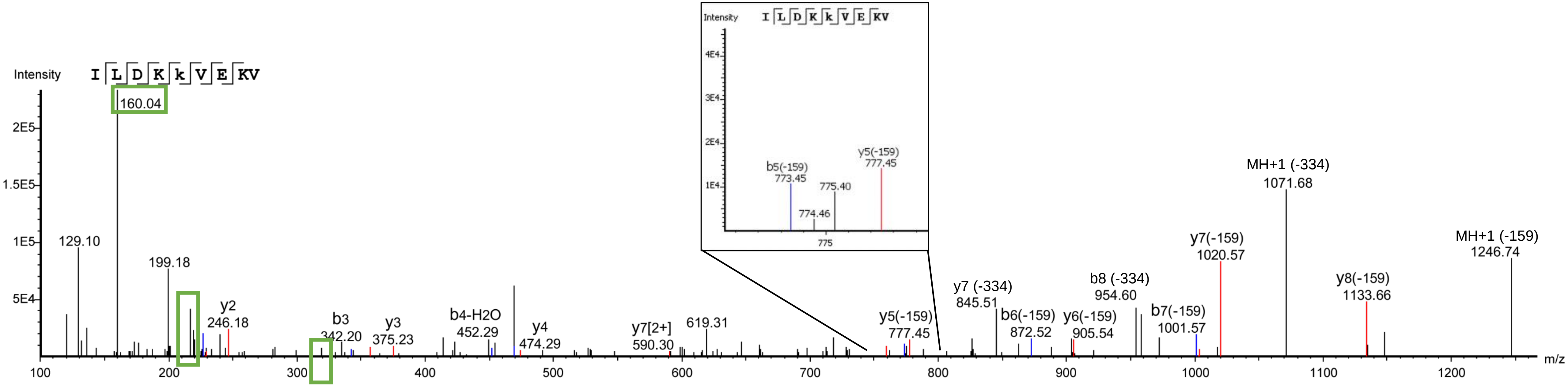

Support for assignment: Presence of b5 and y5 ion with partial adduct. Lack of b4 ions containing full or partial adduct.

Peptide	Length	Modification(s)	-10lgP	Scan	m/z	charge	RT(min)
ILDKKVEKV	9	BenzylpenicillinK(K)@8;	18.7	13307	469.265	3	44.7898

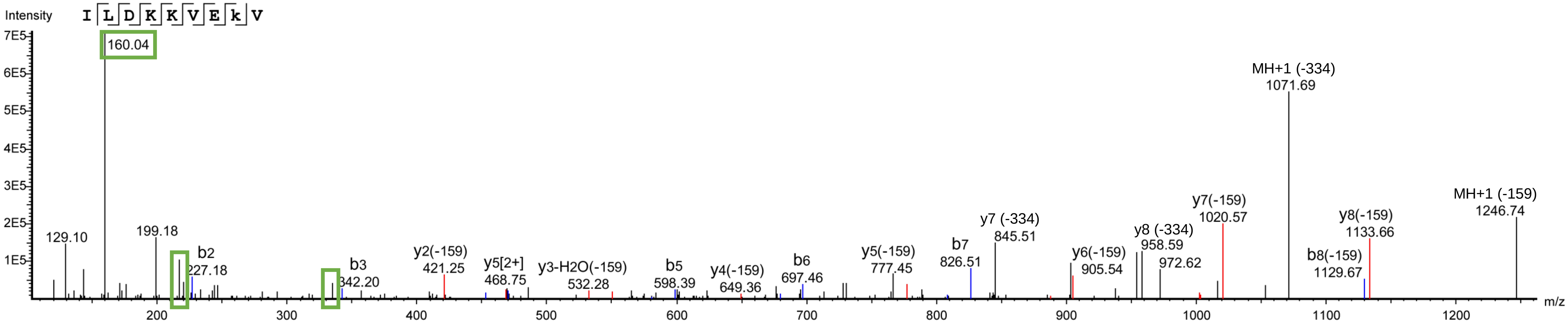

Support for assignment: Presence of y ion series before K4 and K5. Lack of b3 and b4 containing full or partial adduct.

ILDK(BP)KVEKV, Pool 9

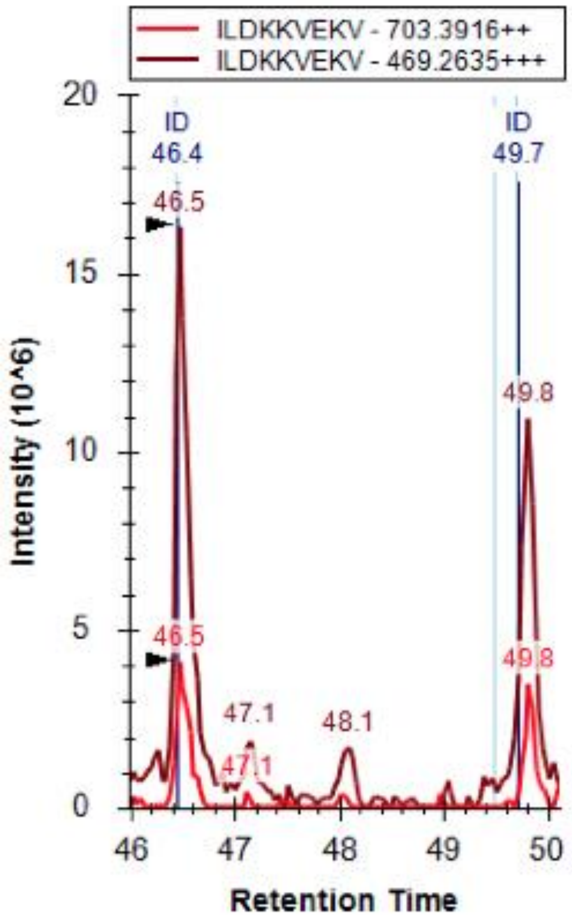

ILDKK(BP)VEKV, Pool 8

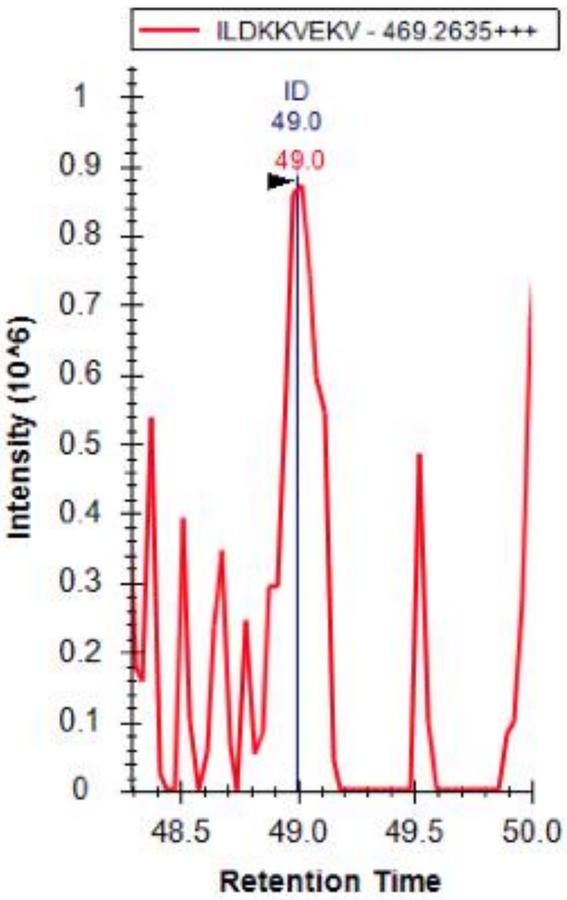

ILDKKVEK(BP)V, Pool 8

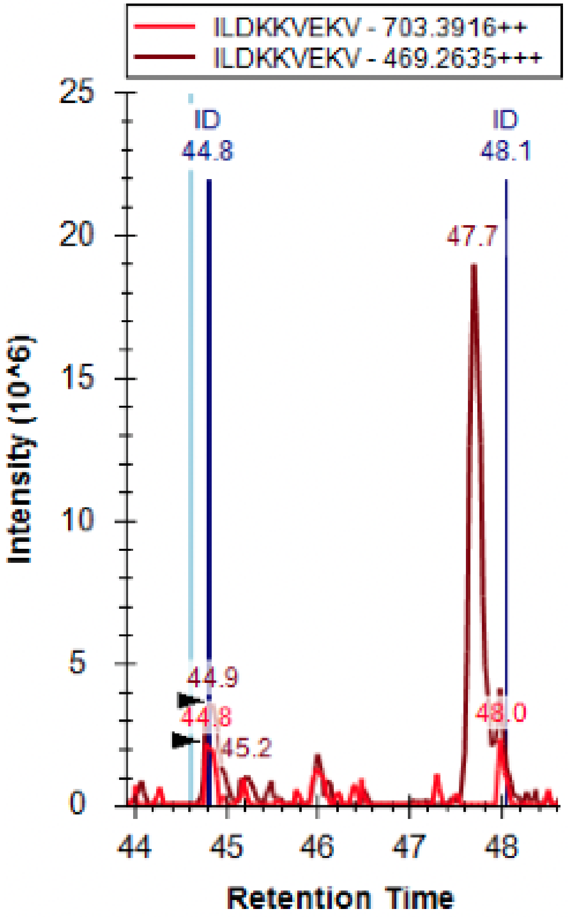

- Benzylpenicillin haptens form diastereoisomers, causing peptides with identical backbones and haptenation sites to elute at 2 different times[1].
- If ILDKKVEKV contained a single modification site, only 2 peaks would be observed.
- 5 distinct peaks were observed, suggesting that there were more than 2 modification sites on ILDKKVEKV.
- This supports distinct modification sites assigned to spectra on slides 35, 36, and 37

[1] Meng, X., Jenkins, R. E., Berry, N. G., Maggs, J. L., Farrell, J., Lane, C. S., ... & Park, B. K. (2011). Direct evidence for the formation of diastereoisomeric benzylpenicilloyl haptens from benzylpenicillin and benzylpenicillenic acid in patients. Journal of Pharmacology and Experimental Therapeutics, 338(3), 841-849.

Peptide	Length	Modification(s)	-10lgP	Scan	m/z	charge	RT(min)
ILMEHIHKL	9	BenzylpenicillinK(K)@8;	28.6	18161	734.381	2	57.3693

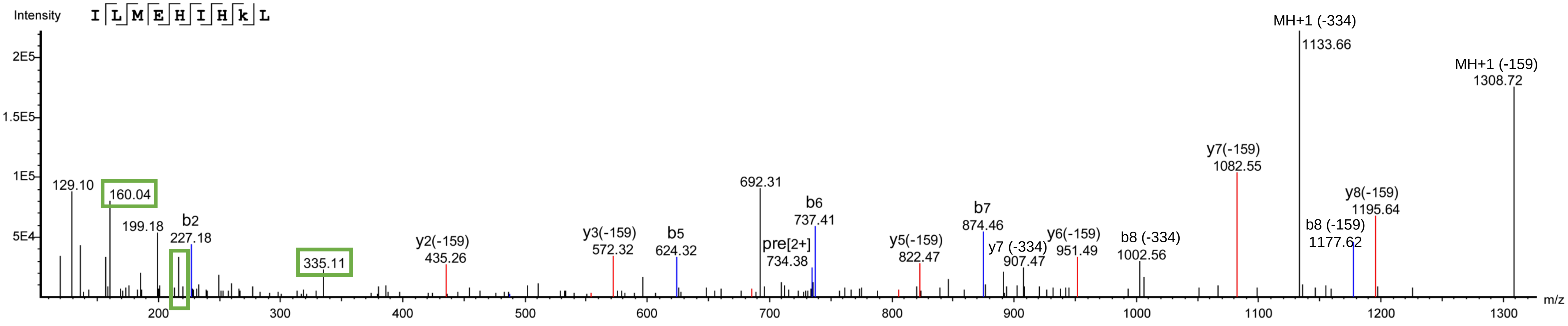

Support for assignment: Presence of y2 with partial adduct.
Lack of b ions with partial or full adduct before K8.

Peptide	Length	Modification(s)	-10lgP	Scan	m/z	charge	RT(min)
ILMEHIHKL	9	Oxidation(M)@3;BenzylpenicillinK(K)@8;	25.14	15418	495.254	3	50.7902

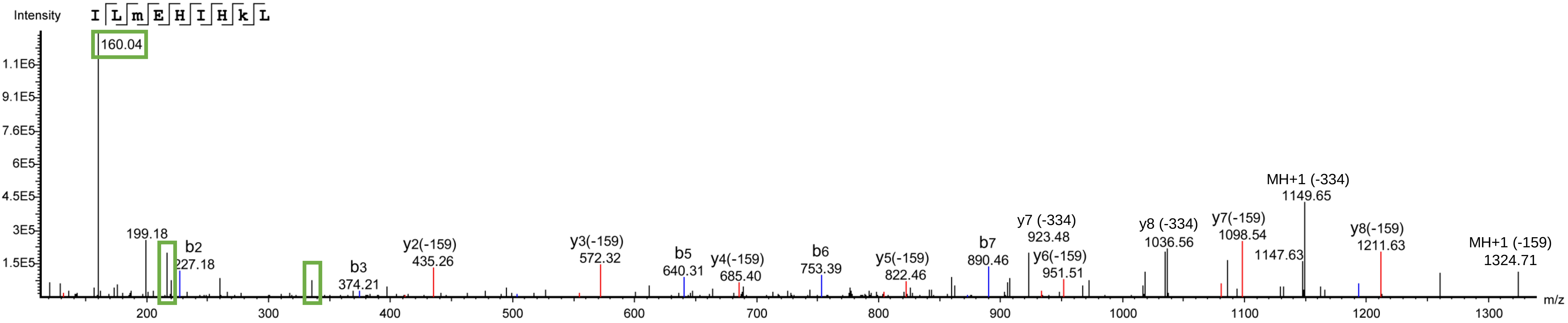

Support for assignment: Presence of y2 with partial adduct.
Lack of b ions with partial or full adduct before K8.

Peptide	Length	Modification(s)	-10lgP	Scan	m/z	charge	RT(min)
ILMEHIHKLKA	11	Oxidation(M)@3;BenzylpenicillinK(K)@8;	31.59	12223	421.476	4	41.7707

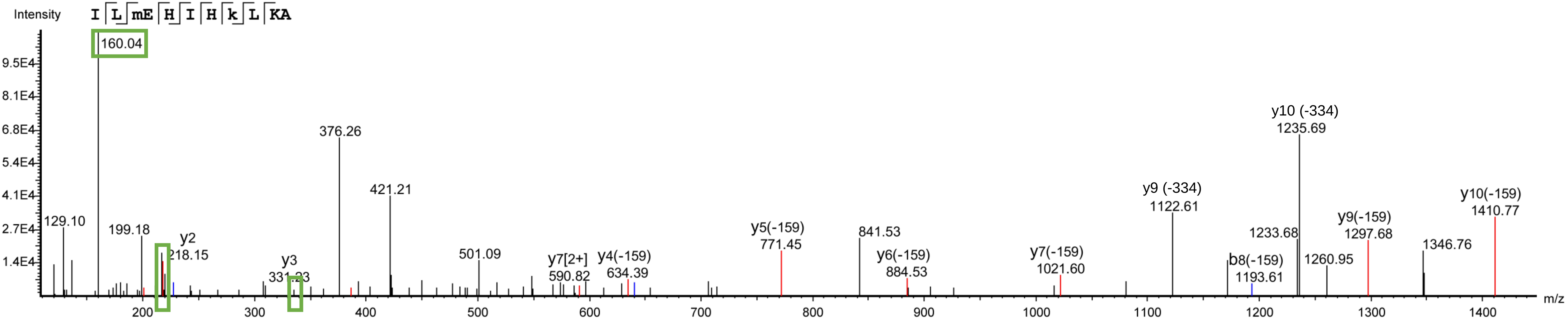

Support for assignment: Lack of b5, b7, and b8 with partial or full adduct. Lack of y2 with partial or full adduct. Presence of y4 and b8 with partial adduct.

Peptide	Length	Modification(s)	-10lgP	Scan	m/z	charge	RT(min)
KLCDVTTGL	9	BenzylpenicillinCC(C)@3;	11.9	26373	701.807	2	77.1436

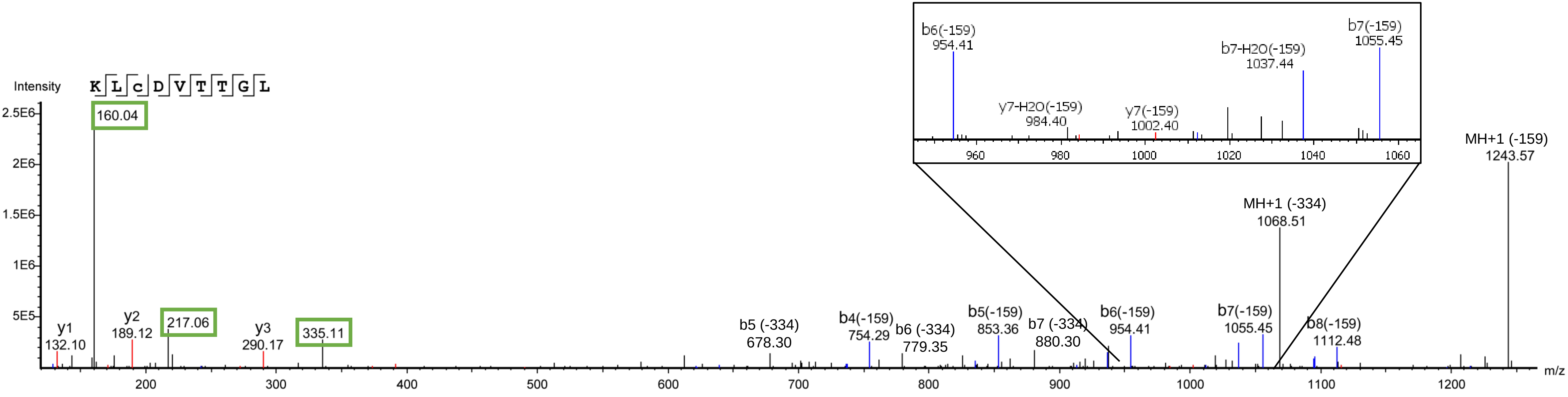

Support for assignment: Presence of y7 ion with partial adduct.

Peptide	Length	Modification(s)	-10lgP	Scan	m/z	charge	RT(min)
KLLEKAFSI	9	BenzylpenicillinK(K)@1;	33.08	25169	691.873	2	74.4012

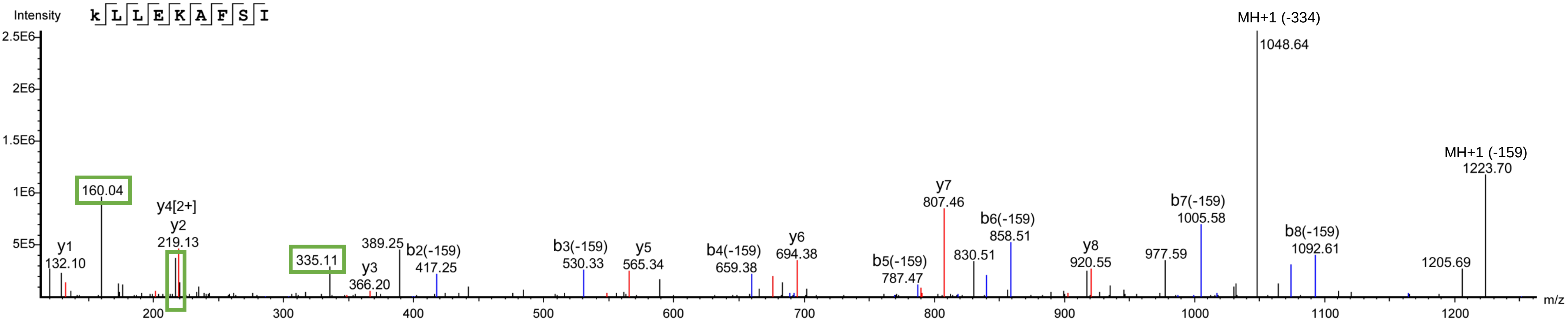

Support for assignment: Presence of b2, b3, and b4 ions with partial before K5. Lack of y ions containing partial or full adduct.

Peptide	Length	Modification(s)	-10lgP	Scan	m/z	charge	RT(min)
KLNPQQFEV	9	BenzylpenicillinK(K)@1;	18.76	25703	718.851	2	76.5485

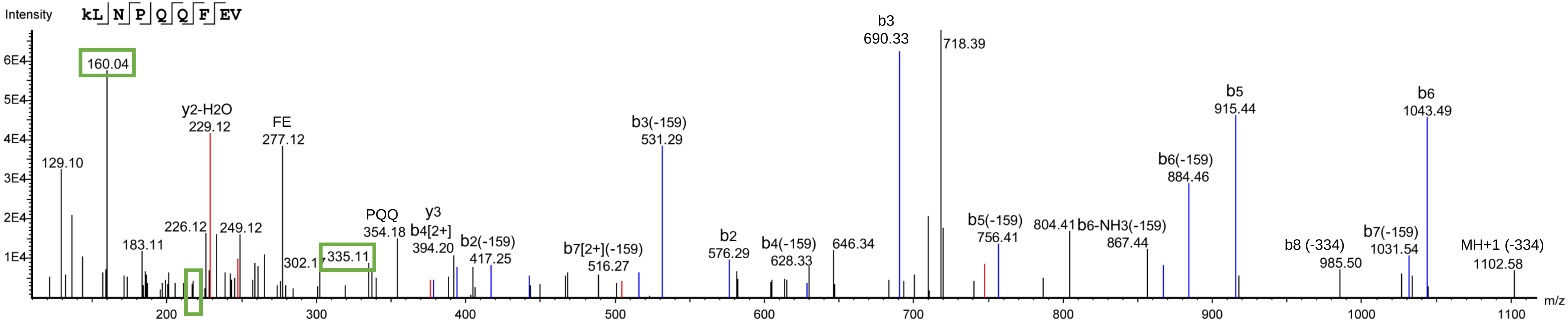

Peptide	Length	Modification(s)	-10lgP	Scan	m/z	charge	RT(min)
KMVDGVGVCTV	11	BenzylpenicillinCC(C)@9;	17.39	22589	780.833	2	67.8471

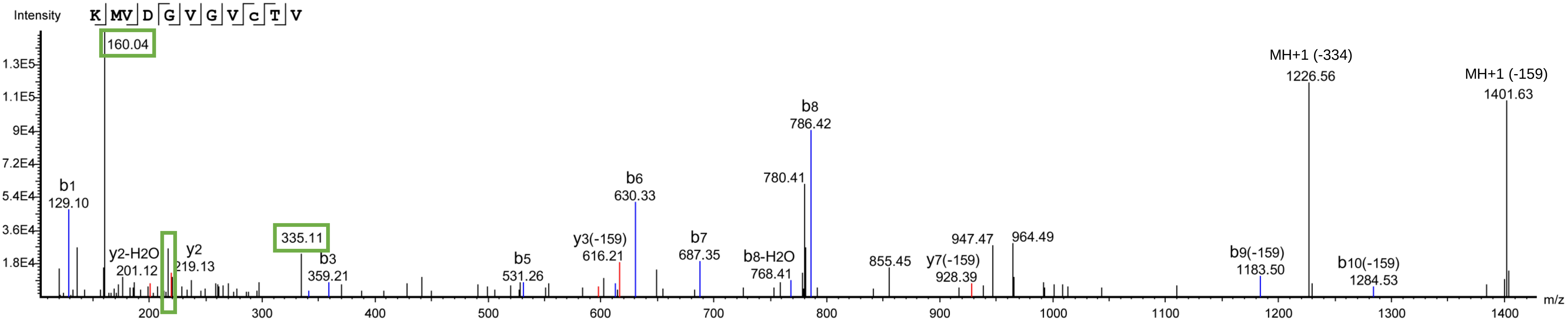

Support for assignment: Presence of y3 and y7 ions with partial adduct before K1. Lack of b ions with partial or full adduct before C9.

Peptide	Length	Modification(s)	-10lgP	Scan	m/z	charge	RT(min)
KMVDGVGVCTV	11	Oxidation(M)@2;BenzylpenicillinCC(C)@9;	17.9	20639	526.224	3	62.7896

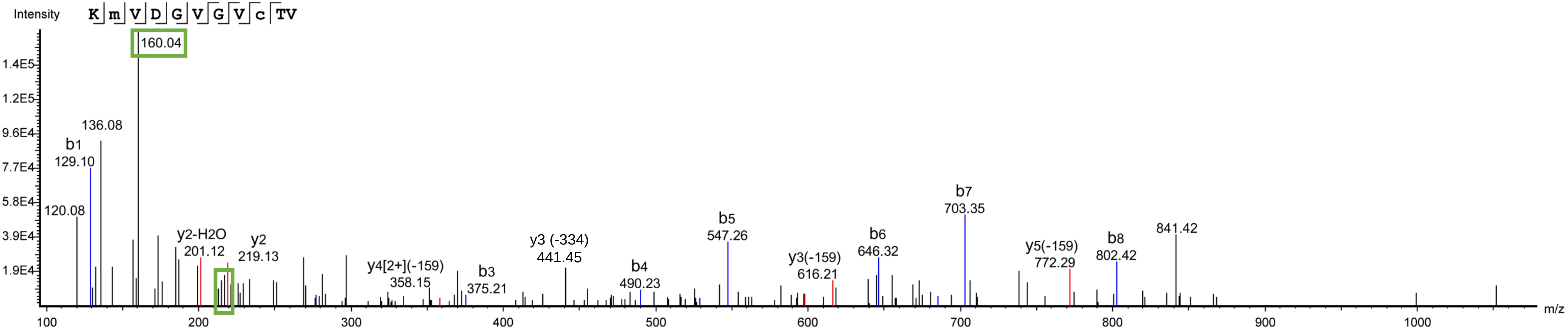

Support for assignment: Presence of y3 with partial adduct or no adduct, and y5 with partial adduct before K1. Lack of b ion series with partial or full adduct before C9.

Peptide	Length	Modification(s)	-10lgP	Scan	m/z	charge	RT(min)
LLDVTCKTV	9	BenzylpenicillinCC(C)@6;	18.06	23994	722.832	2	71.3917

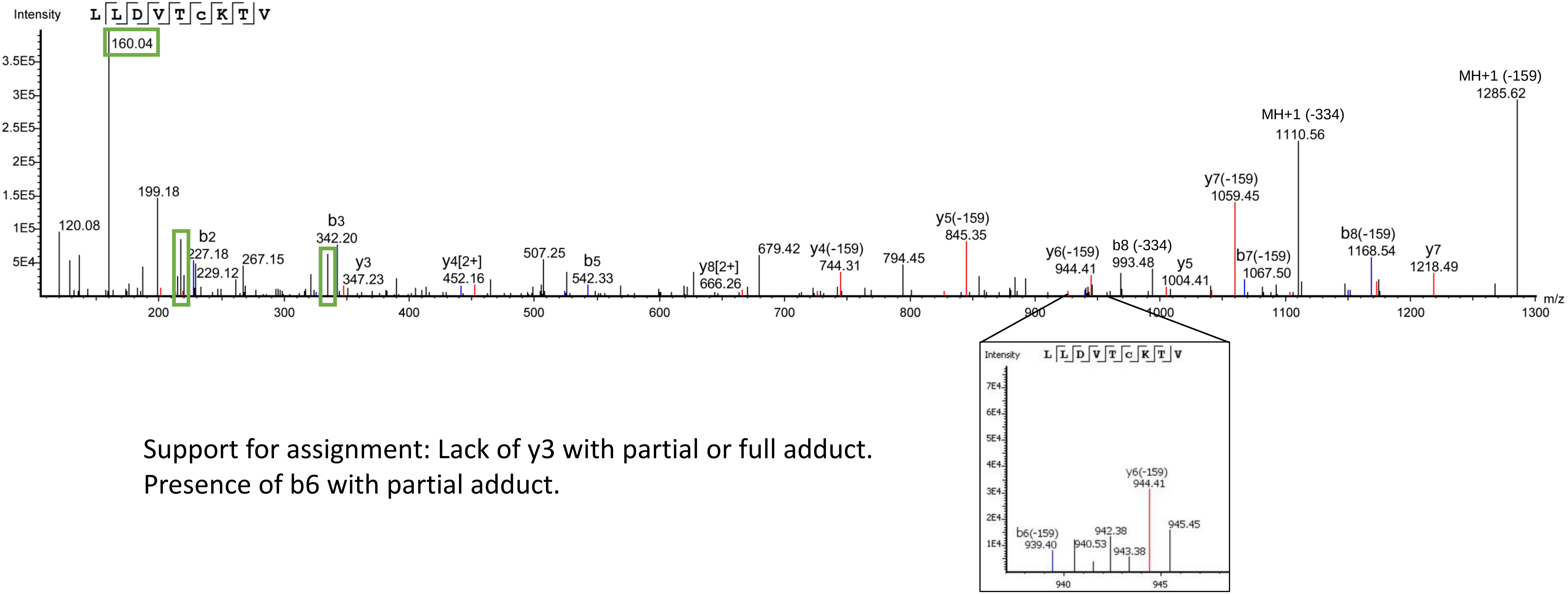

Support for assignment: Lack of y3 with partial or full adduct.
Presence of b6 with partial adduct.

Peptide	Length	Modification(s)	-10lgP	Scan	m/z	charge	RT(min)
LLEPCIPSV	9	BenzylpenicillinCC(C)@5;	19.66	26462	712.32	2	77.39

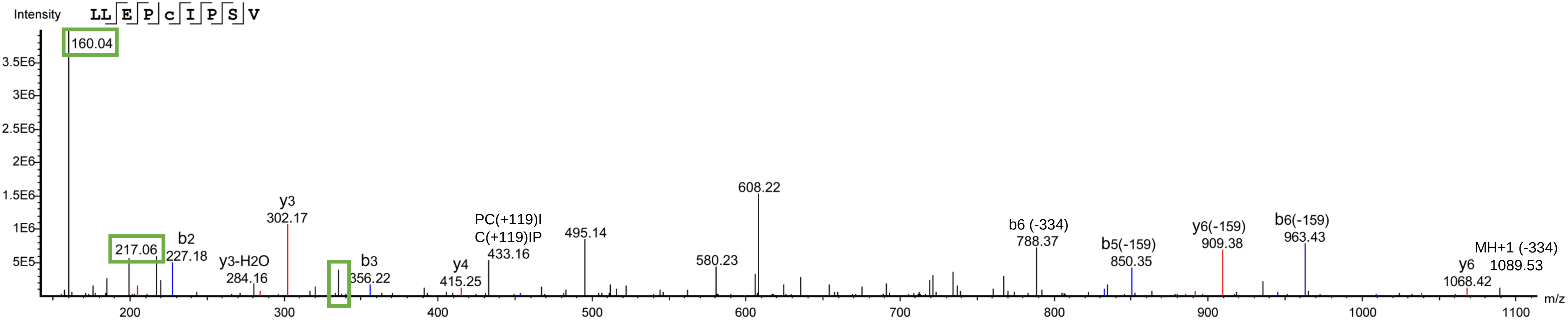

Peptide	Length	Modification(s)	-10lgP	Scan	m/z	charge	RT(min)
LLLPPPPCPA	10	BenzylpenicillinCC(C)@8;	32.92	26002	735.847	2	77.3089

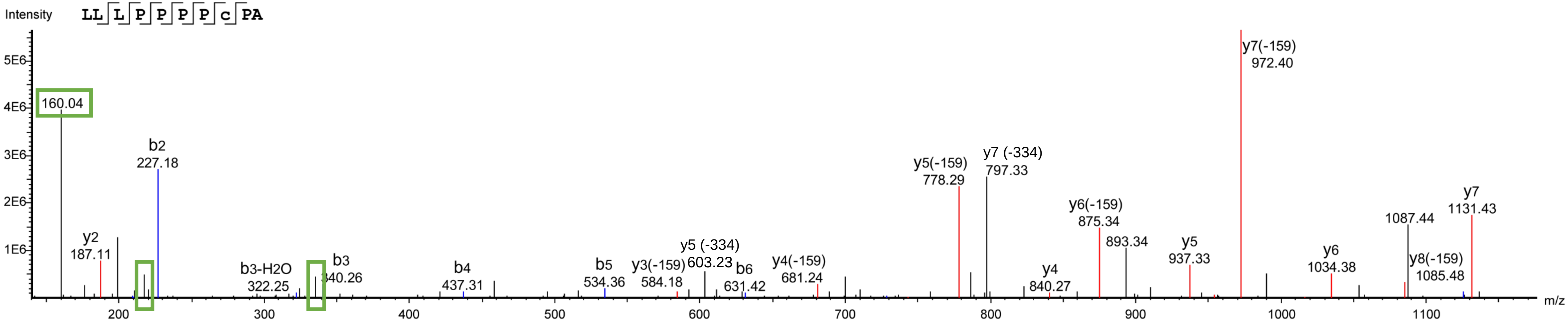

Peptide	Length	Modification(s)	-10lgP	Scan	m/z	charge	RT(min)
LLPPPPCPA	9	BenzylpenicillinCC(C)@7;	30.37	25008	679.306	2	75.2601

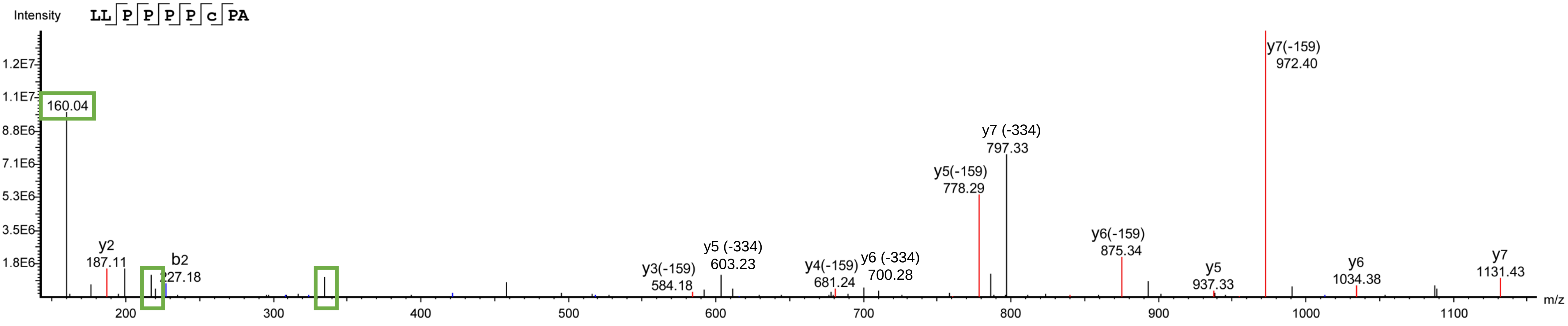

Peptide	Length	Modification(s)	-10lgP	Scan	m/z	charge	RT(min)
LLPPPPCPA	9	BenzylpenicillinC(C)@7;	20.71	25212	619.801	2	75.0591

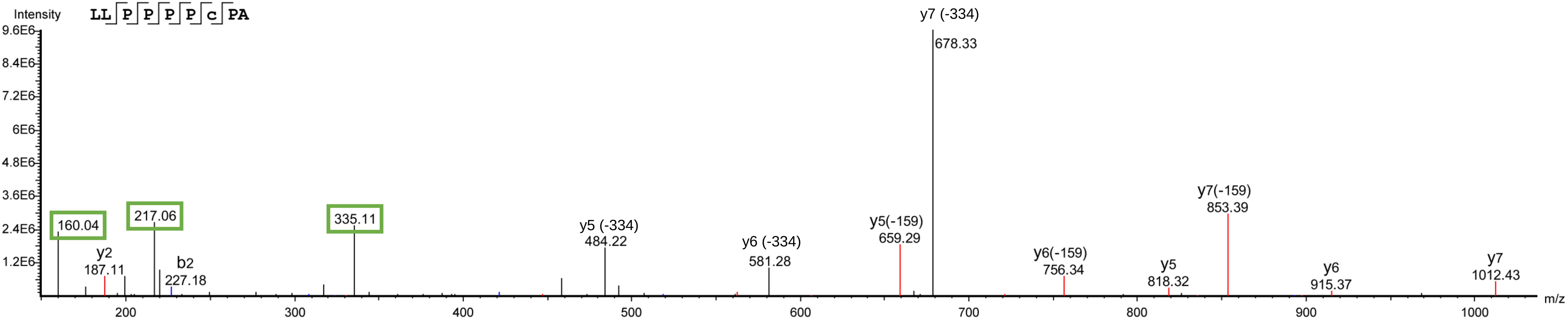

Ambiguous

Peptide	Length	Modification(s)	-10lgP	Scan	m/z	charge	RT(min)
NLAKCIVSV	9	BenzylpenicillinCC(C)@8;	15.53	25092	700.328	2	74.3966

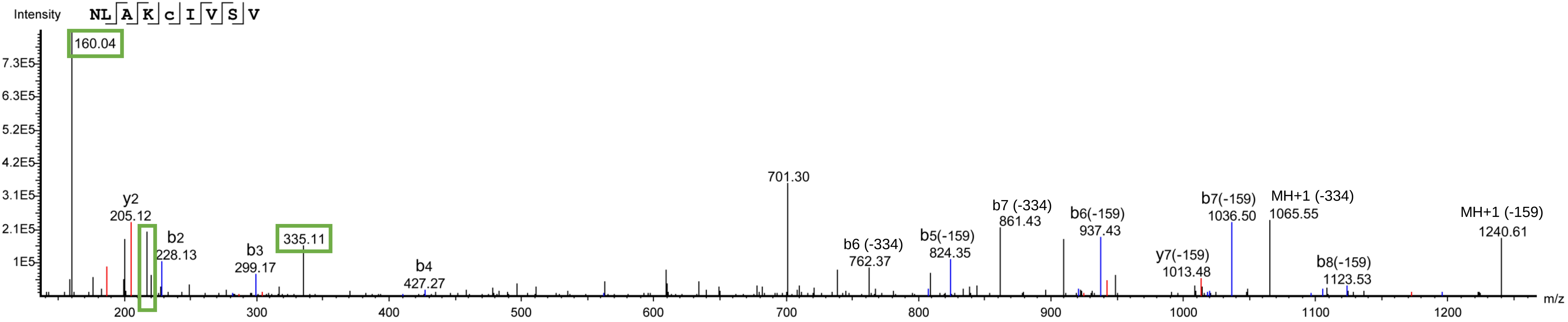

Ambiguous. Lack of y5 or b4 with partial or full adduct.

Peptide	Length	Modification(s)	-10lgP	Scan	m/z	charge	RT(min)
RLMQGDEICL	10	Oxidation(M)@3;BenzylpenicillinCC(C)@9;	16.55	24263	549.564	3	73.0013

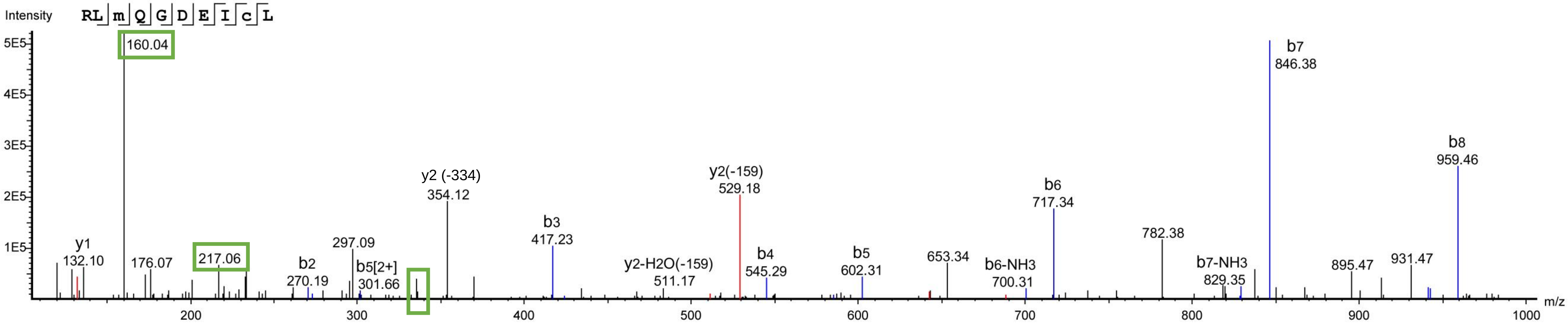

Support for assignment: Lack of b ions with partial or full adduct before C9. Presence of y2 ions with partial adduct or no adduct before R1.

Ambiguous

Peptide	Length	Modification(s)	-10lgP	Scan	m/z	charge	RT(min)
RMIKEKLCYV	10	BenzylpenicillinCC(C)@8;	15.5	10671	579.275	3	51.5814

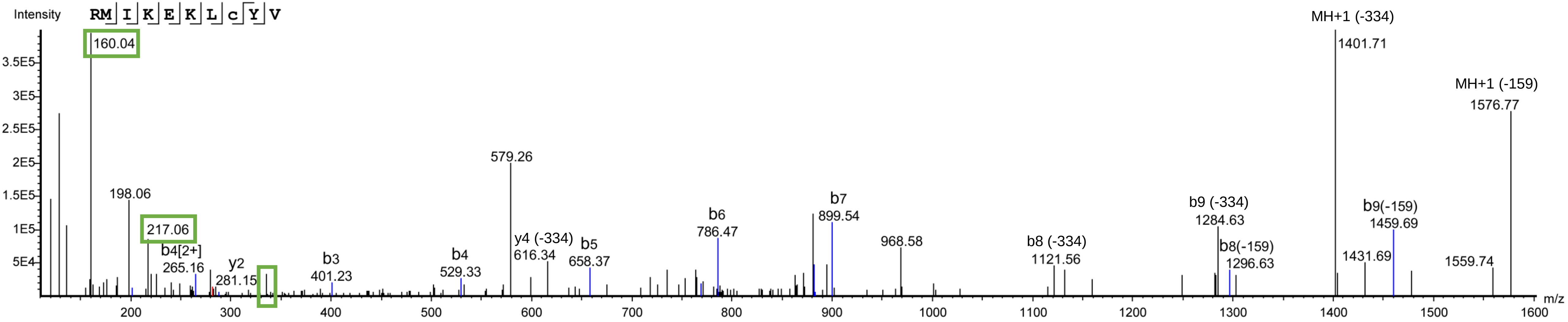

Ambiguous. Lack of b4 and b6 with either full or partial adduct. Presence of b8 and b9 with partial adduct. Lack of y3 with either full or partial adduct.

Low confidence

Peptide	Length	Modification(s)	-10lgP	Scan	m/z	charge	RT(min)
SLGERVHQL	9	BenzylpenicillinR(R)@5;	12.4	12405	686.849	2	42.9543

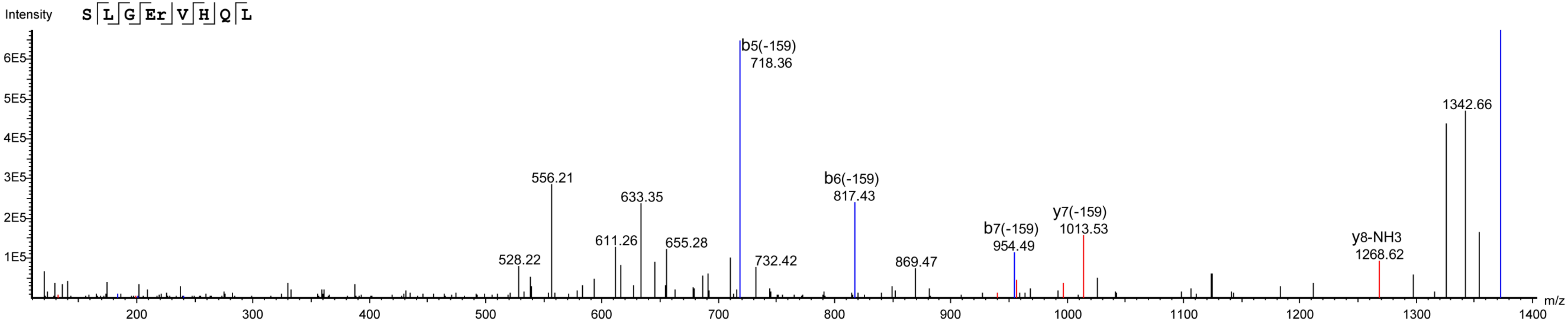

Low number of annotated fragments. m/z 160.04 < 10%.

Peptide	Length	Modification(s)	-10lgP	Scan	m/z	charge	RT(min)
SLHDALCVV	9	BenzylpenicillinCC(C)@7;	19.95	25018	705.3	2	75.2835

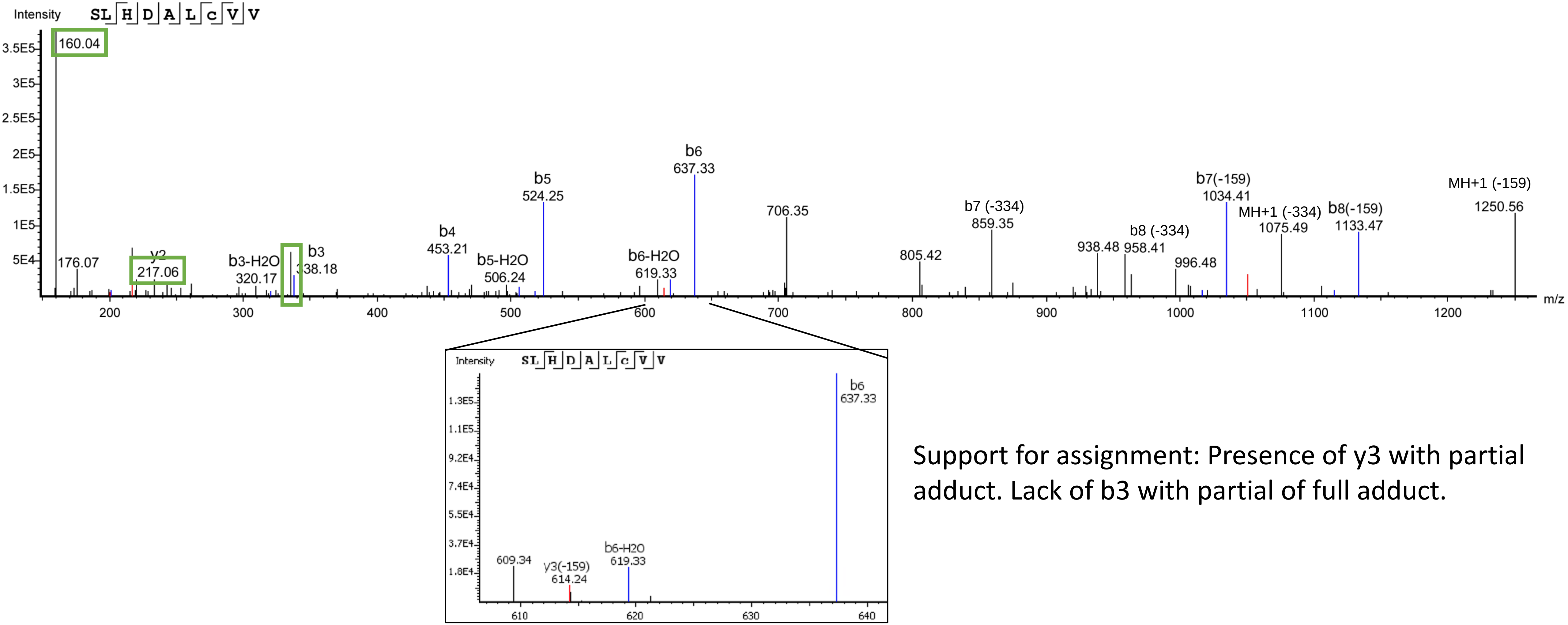

Support for assignment: Presence of y3 with partial adduct. Lack of b3 with partial of full adduct.

Ambiguous

Peptide	Length	Modification(s)	-10lgP	Scan	m/z	charge	RT(min)
SLMEESGICKV	11	BenzylpenicillinCC(C)@9;	26.88	24221	824.848	2	72.8881

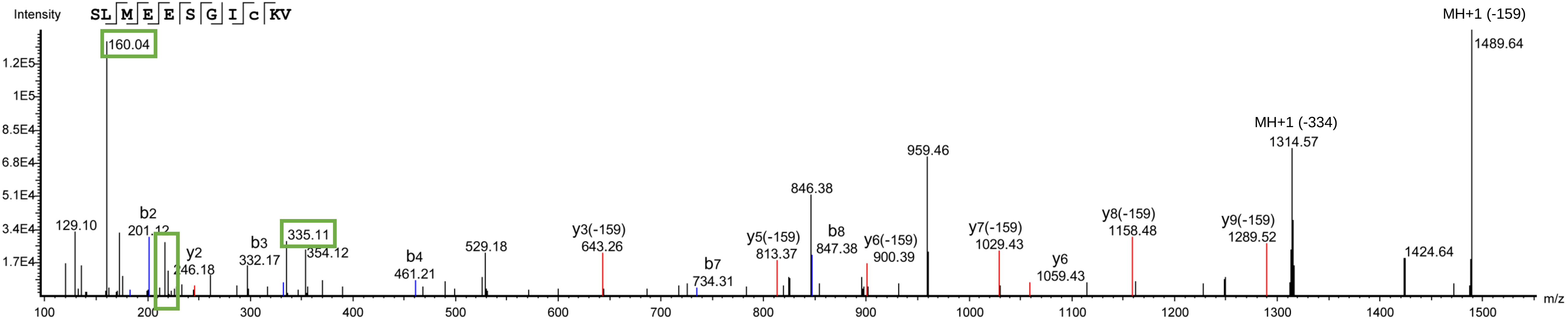

Ambiguous. Lack of b9 ion with partial or full adduct. But lack of y2 with partial or full adduct. Presence of y series ions with partial or full adduct.

Ambiguous

Peptide	Length	Modification(s)	-10lgP	Scan	m/z	charge	RT(min)
SLMEESGICKV	11	Oxidation(M)@3;BenzylpenicillinCC(C)@9;	17.64	24221	832.843	2	64.9707

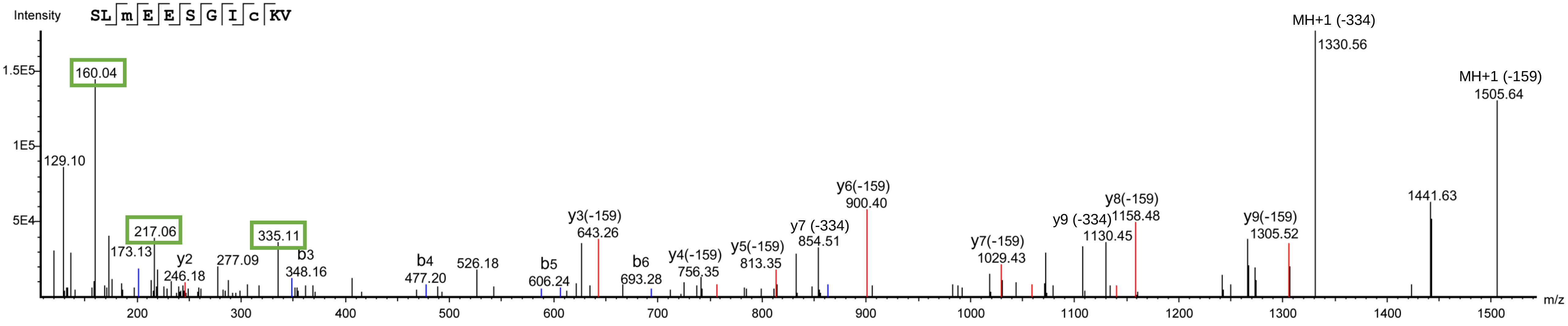

Ambiguous. Lack of y2 ions with partial or full adduct. Lack of b9 with partial or full adduct.

Peptide	Length	Modification(s)	-10lgP	Scan	m/z	charge	RT(min)
TLCPAIHKL	9	BenzylpenicillinCC(C)@3;	20.57	20069	483.562	3	61.784

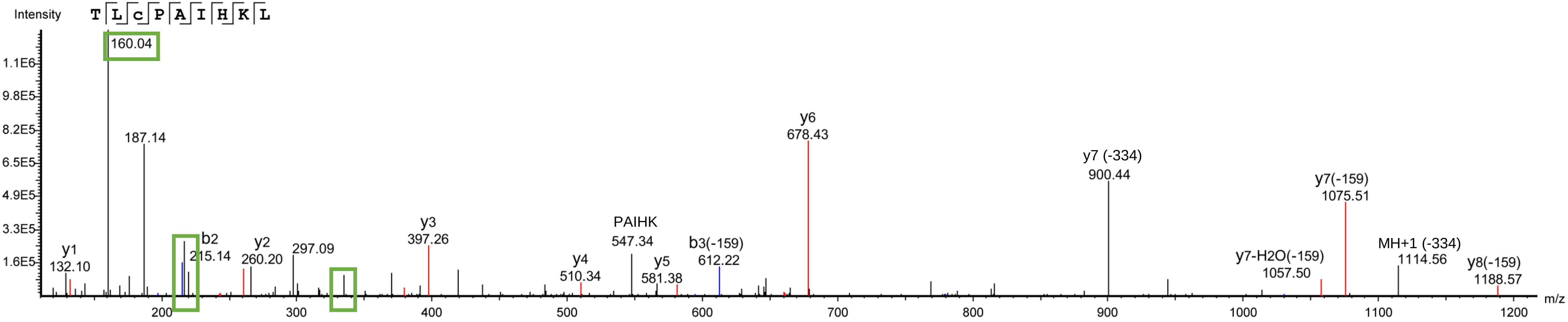

Support for assignment: Presence of b3 with partial adduct.
Lack of y2 with partial or full adduct.

Peptide	Length	Modification(s)	-10lgP	Scan	m/z	charge	RT(min)
TLLEKVEGC	9	BenzylpenicillinCC(C)@9;	22.58	22775	722.812	2	68.8646

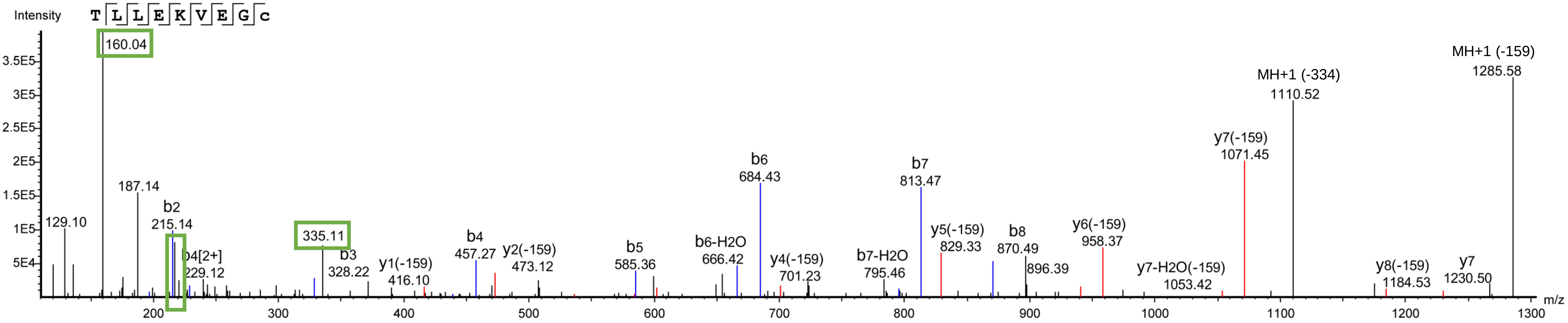

Support for assignment: Presence of y1 with partial adduct.
Lack of b ions containing partial or full adduct before C9.

Peptide	Length	Modification(s)	-10lgP	Scan	m/z	charge	RT(min)
VLAELKGVTC	10	BenzylpenicillinCC(C)@10;	31.73	22491	743.345	2	68.1283

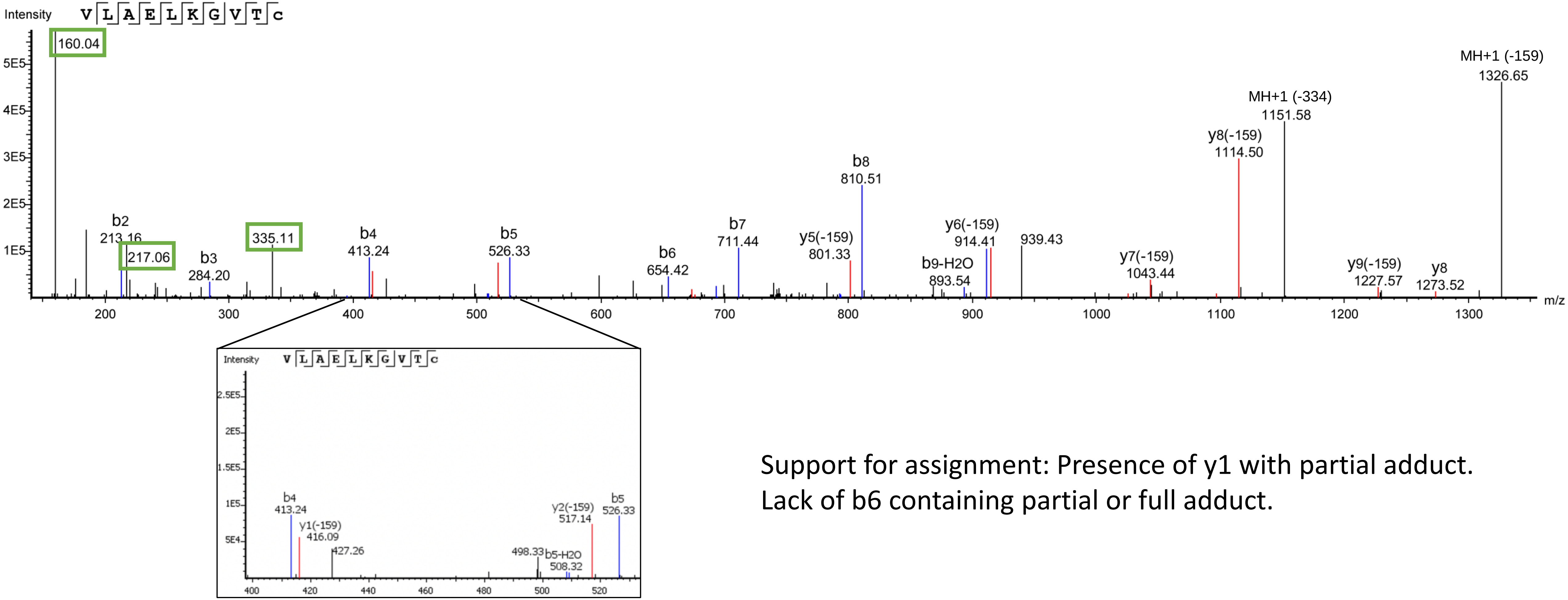

Support for assignment: Presence of y1 with partial adduct.
Lack of b6 containing partial or full adduct.

Peptide	Length	Modification(s)	-10lgP	Scan	m/z	charge	RT(min)
VLDELKNMKC	10	BenzylpenicillinCC(C)@10;	18.03	19255	549.5782	3	59.28

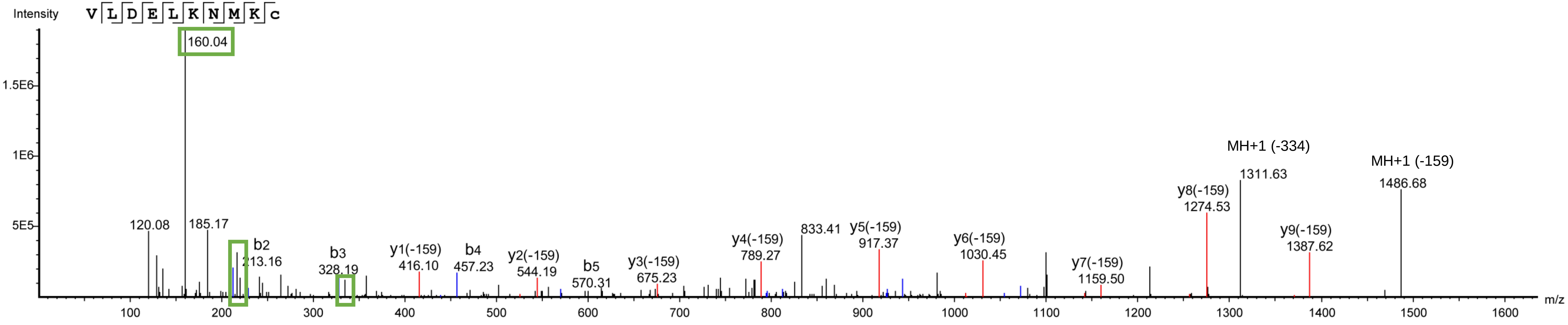

Support for assignment: Presence of y1 with partial adduct.
Lack of b9 containing partial or full adduct.

Incorrect

Peptide	Length	Modification(s)	-10lgP	Scan	m/z	charge	RT(min)
VLDELKNMKC	10	BenzylpenicillinK(K)@9;Cysteinyl(C)@10;	12.25	19115	549.245	3	59.3349

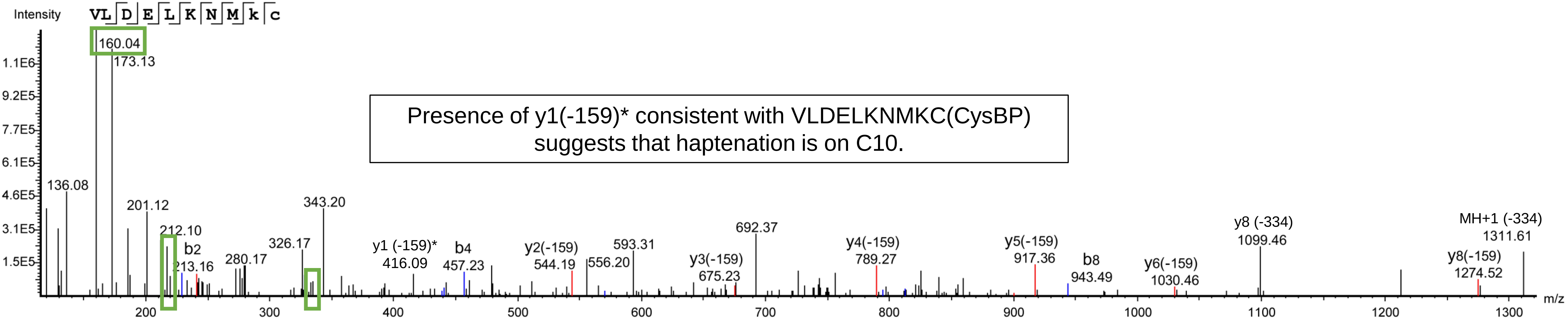

Incorrect modification assignment. Presence of y1 with partial adduct consistent with VLDELKNMKC(CysBP) .

VLDELKNMKC(CysBP), Pool 6

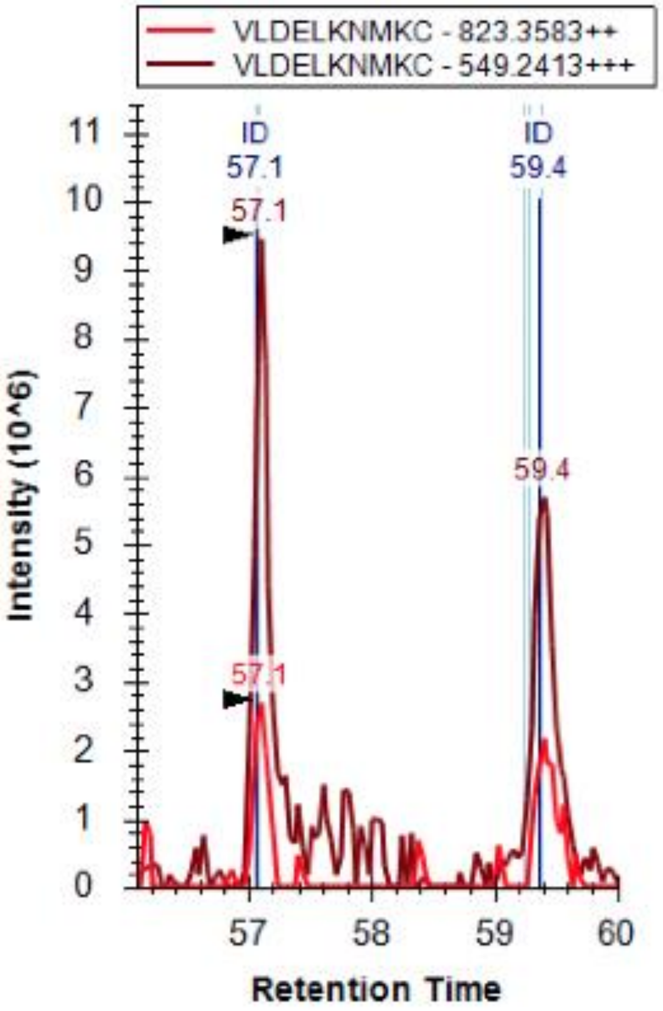

VLDELKNMK(BP)C(Cys), Pool 6

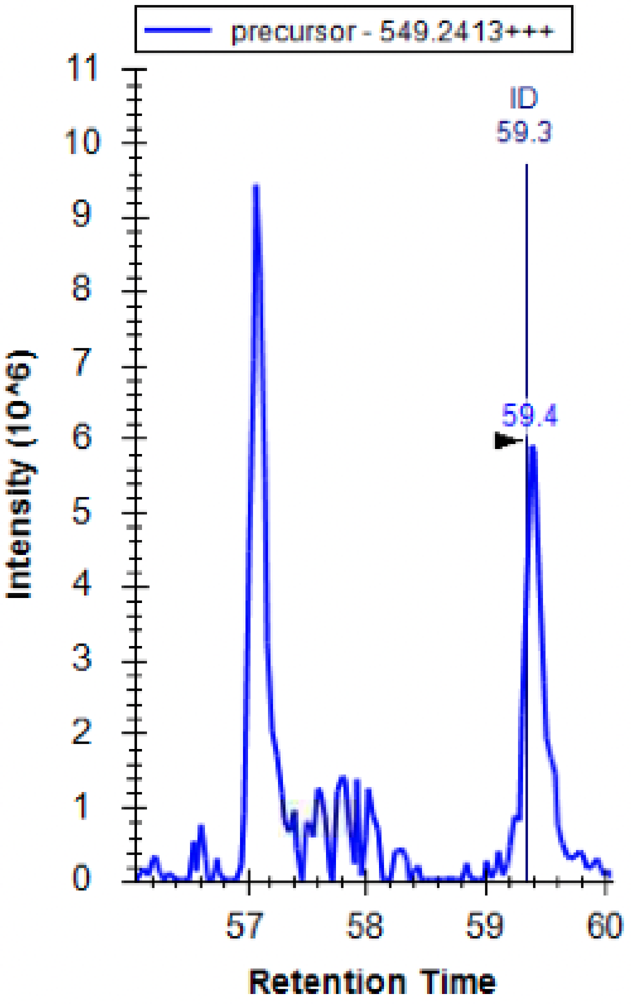

- Benzylpenicillin haptens forms diastereoisomers, causing peptides with identical backbones and haptenation site to elute at 2 different times[1].
- Only 2 RT peaks were observed, suggesting a single modification site is possible.
- We noted that the spectra assigned VLDELKNMK(BP)C(Cys) contained evidence of y1 with a partial BP adduct in slide 63, consistent with CysBP on the C terminal cysteine.
- Taken together, it is likely that the precursor at 57.1min and 59.4min with m/z 549.24 should be assigned VLDELKNMKC(CysBP).

[1] Meng, X., Jenkins, R. E., Berry, N. G., Maggs, J. L., Farrell, J., Lane, C. S., ... & Park, B. K. (2011). Direct evidence for the formation of diastereoisomeric benzylpenicilloyl haptens from benzylpenicillin and benzylpenicillenic acid in patients. Journal of Pharmacology and Experimental Therapeutics, 338(3), 841-849.

Peptide	Length	Modification(s)	-10lgP	Scan	m/z	charge	RT(min)
VLDELKNMKC	10	Oxidation(M)@8;BenzylpenicillinCC(C)@10;	14.39	15178	554.5733	3	49.16

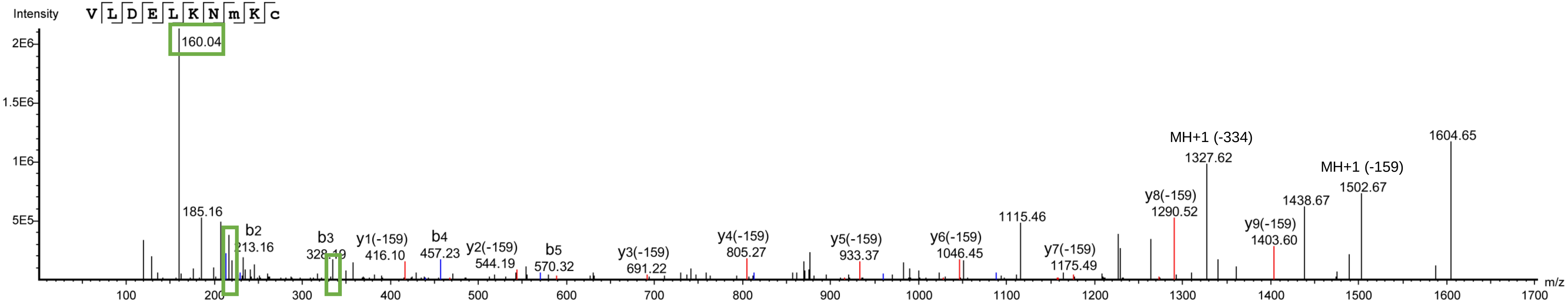

Correct assignment. Presence of y1 with partial adduct. Lack of b ions containing partial or full adduct before C10.

Incorrect

Peptide	Length	Modification(s)	-10lgP	Scan	m/z	charge	RT(min)
VLDELKNMKC	10	Oxidation(M)@8;BenzylpenicillinK(K)@9;Cysteinyl(C)@10;	12.39	15212	554.575	3	49.2507

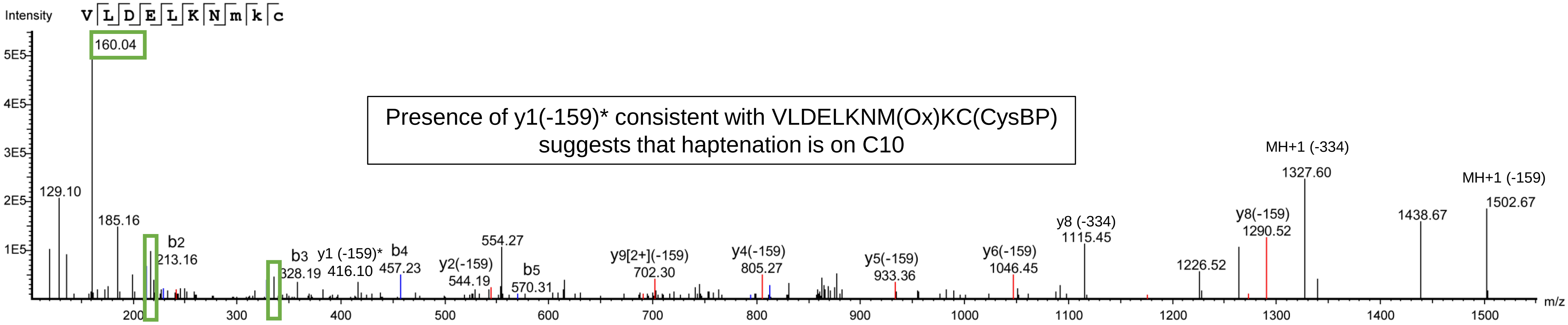

Incorrect modification assignment. Presence of y1 with partial adduct consistent with VLDELKNM(Ox)KC(CysBP) .

VLDELKNM(Ox)KC(CysBP), Pool 5

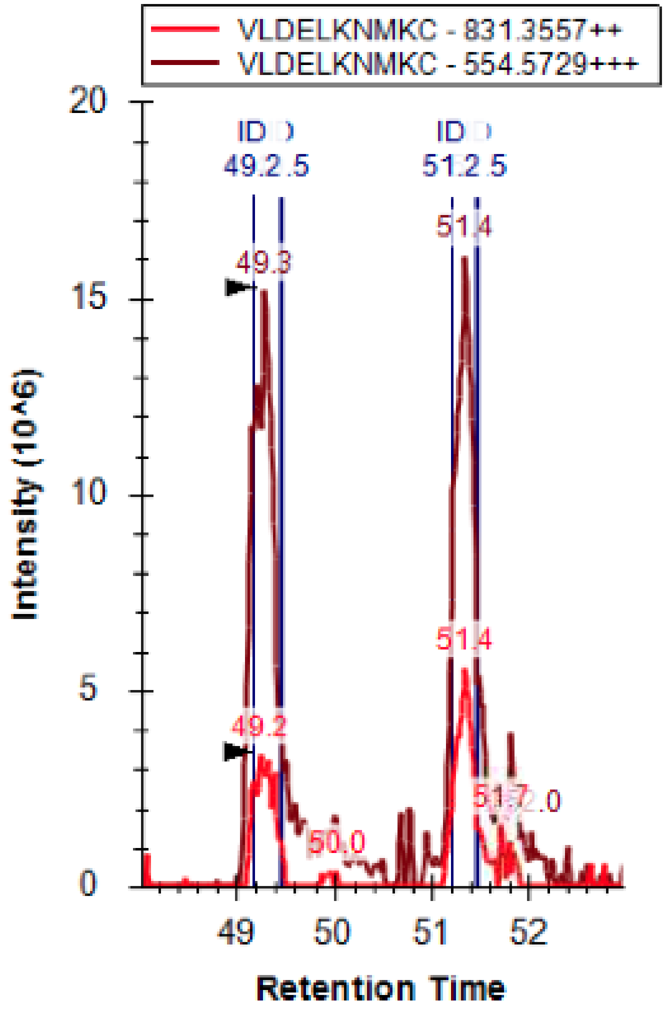

VLDELKNM(Ox)K(BP)C(Cys), Pool 5

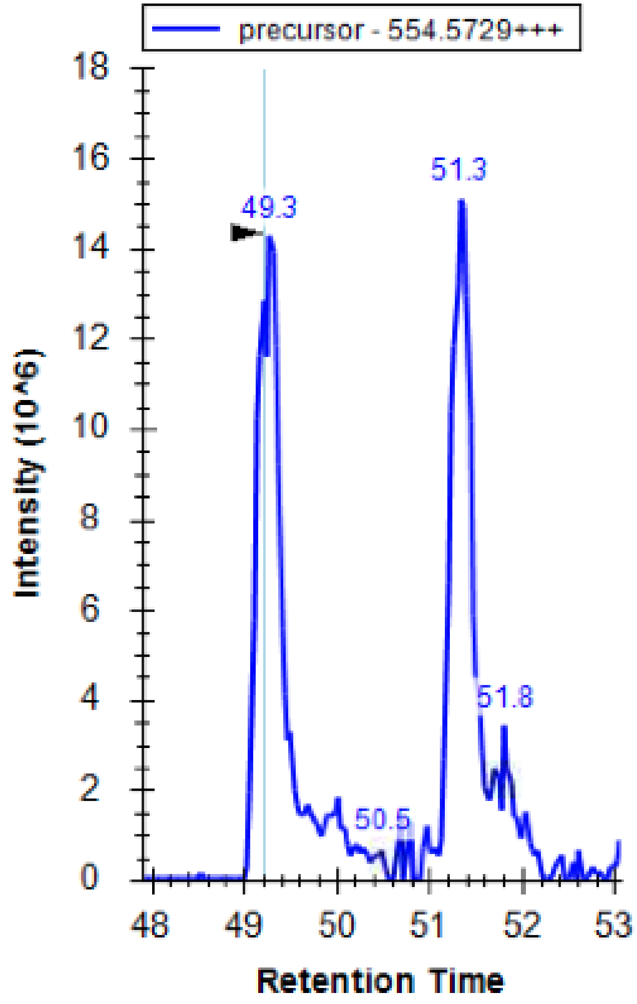

- Benzylpenicillin haptens forms diastereoisomers, causing peptides with identical backbones and haptenation site to elute at 2 different times[1].
- Only 2 RT peaks were observed, suggesting a single modification site is possible.
- We noted that the spectra assigned VLDELKNM(Ox)K(BP)C(Cys) contained evidence of y1 with a partial BP adduct in slide 66, consistent with CysBP on the C terminal cysteine.
- Taken together, it is likely that the precursor at 49.3min and 51.4min with m/z 554.58 should be assigned VLDELKNM(Ox)KC(CysBP).

[1] Meng, X., Jenkins, R. E., Berry, N. G., Maggs, J. L., Farrell, J., Lane, C. S., ... & Park, B. K. (2011). Direct evidence for the formation of diastereoisomeric benzylpenicilloyl haptens from benzylpenicillin and benzylpenicillenic acid in patients. Journal of Pharmacology and Experimental Therapeutics, 338(3), 841-849.

Peptide	Length	Modification(s)	-10lgP	Scan	m/z	charge	RT(min)
YCAEIAHNV	9	BenzylpenicillinCC(C)@2;	34.77	21123	736.788	2	64.8998

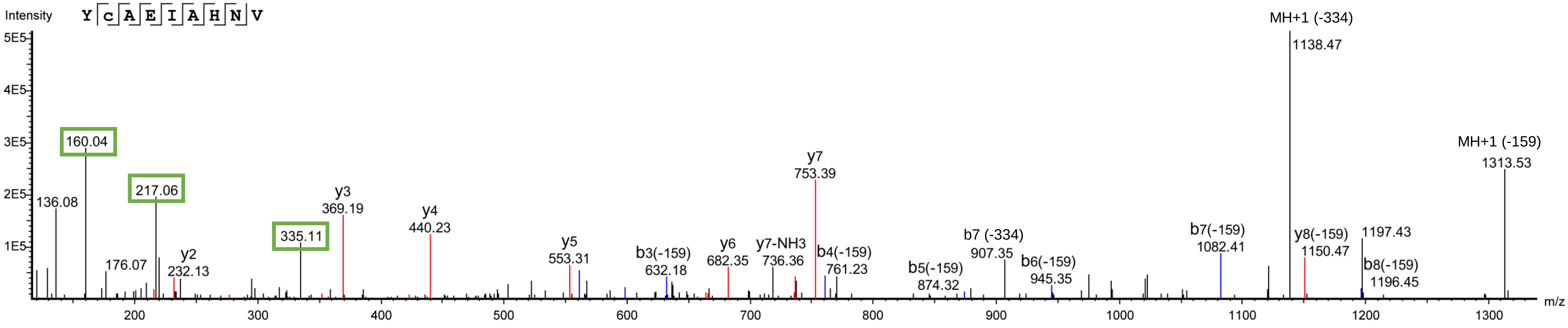

Support for assignment: Presence of b3, b4, b5, and b6 ions with partial adduct before H7. Lack of y ions containing partial or full adduct until C2.

Peptide	Length	Modification(s)	-10lgP	Scan	m/z	charge	RT(min)
YFDPANGKF	9	BenzylpenicillinK(K)@8;	24.74	25998	696.801	2	77.304

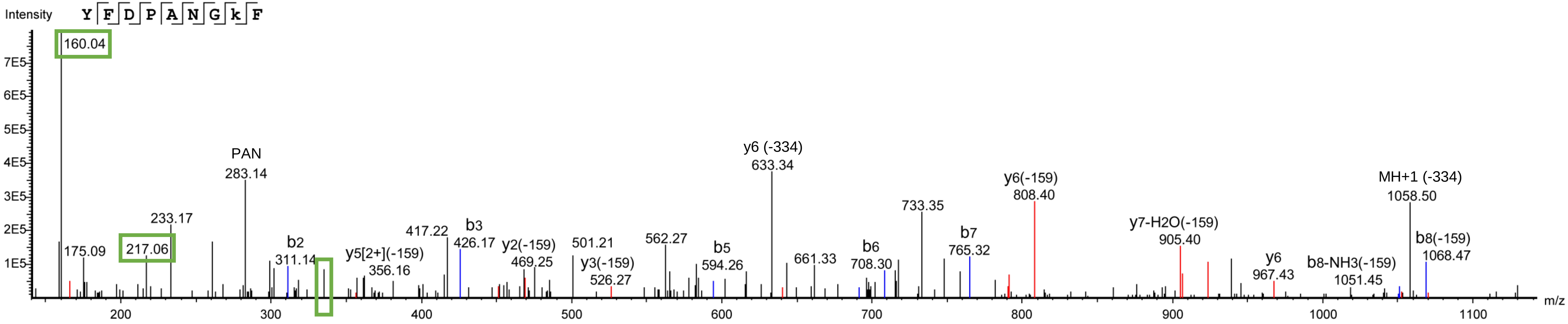

Peptide	Length	Modification(s)	-10lgP	Scan	m/z	charge	RT(min)
YLFKCPQSV	9	BenzylpenicillinCC(C)@5;	15.97	23610	769.332	2	70.3676

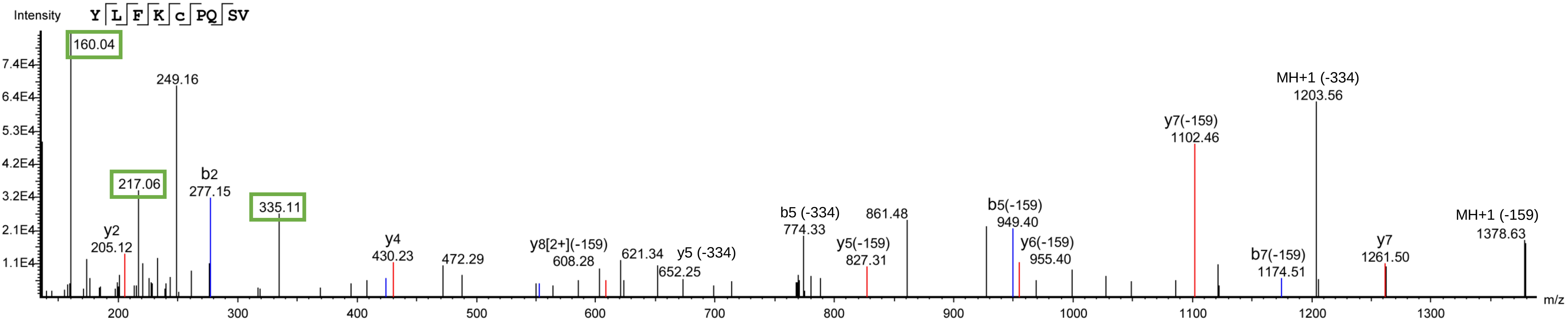

Support for assignment: Lack of b4 with either full or partial adduct. Presence of y5 with partial adduct.
