# Supplementary material for: HLA‐A*02:01 Presents Penicillin‐Modified Cysteinylated Peptides for T Cell Recognition
Source: Allergy. 2025 Sep 4;80(11):3165–77. doi: 10.1111/all.70025 (PMC12590334; doi:10.1111/all.70025)
Supplement: Supplementary file 2 — Appendix S2: Supporting Information. [file ALL-80-3165-s003.pdf]

S. Fig 1

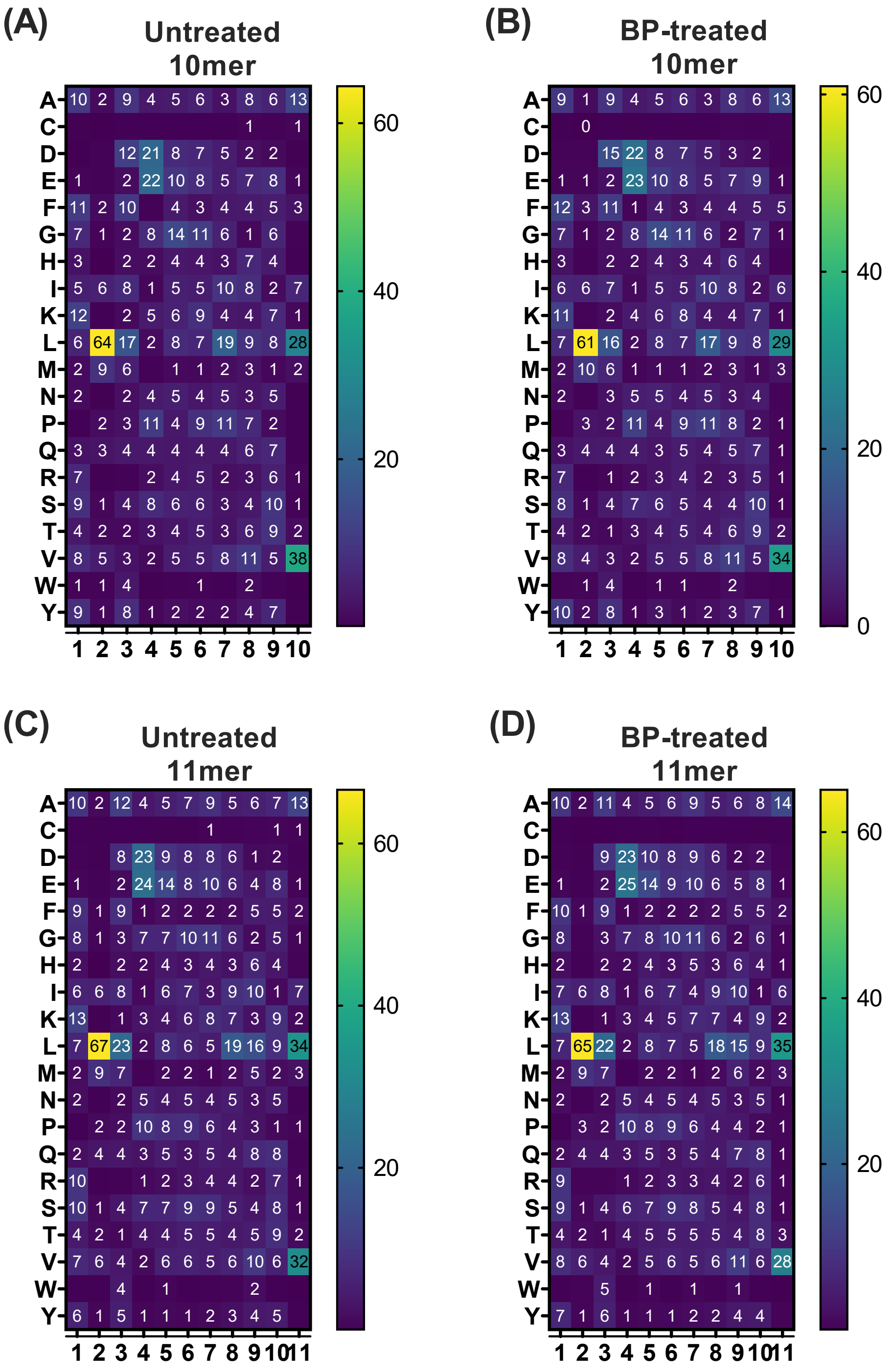

Supplementary figure 1: Amino acid frequency analysis of HLA-A*02:01 ligands isolated from C1R.A*02:01 with BB7.2. Peptides unique by sequence were included in this analysis. (A) 10mer amino acid frequency from the untreated sample. **(B)** 10mer amino acid frequency from the BP-treated sample. **(C)** 11mer amino acid frequency from the untreated sample. **(D)** 11mer amino acid frequency from the BP-treated sample.

S. Fig 2

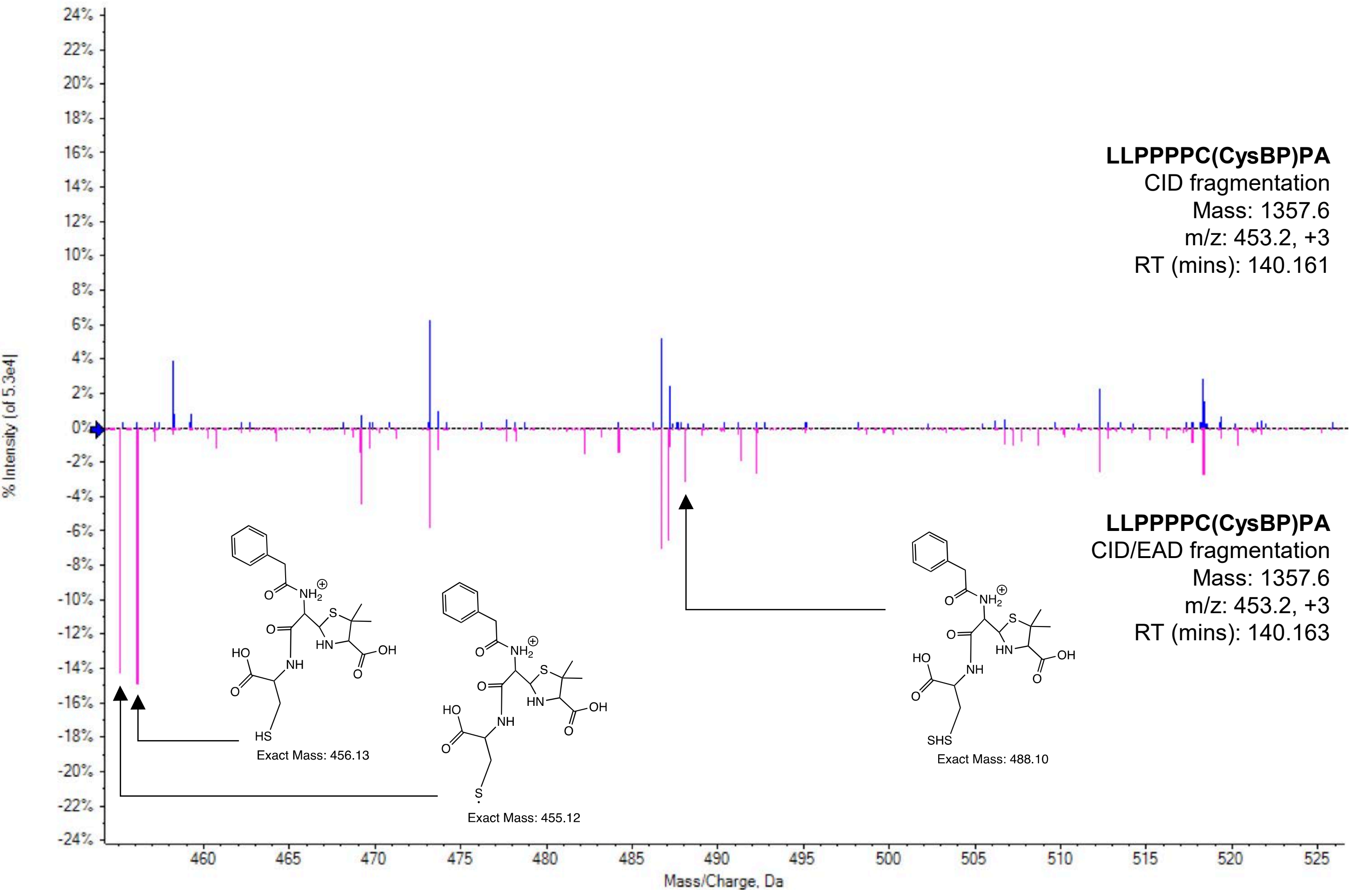

Supplementary figure 2: Targeted fragmentation of LLPPPPC(CysBP)PA using either collision-induced dissociation (CID), or electron activated dissociation with CID (EAD/CID). Mirror plot comparing peptide fragmentation using either CID or EAD/CID, demonstrating fragments consistent with intact CysBP when peptides were subject to EAD/CID fragmentation. Plot is zoomed to m/z 455 - 495, the region containing these ions – Fragment m/z 455.12 = CysBP - Hydrogen; Fragment m/z 456.13 = CysBP; Fragment m/z 488.10 = CysBP + Cysteine persulfide.

S. Fig 3

(A)

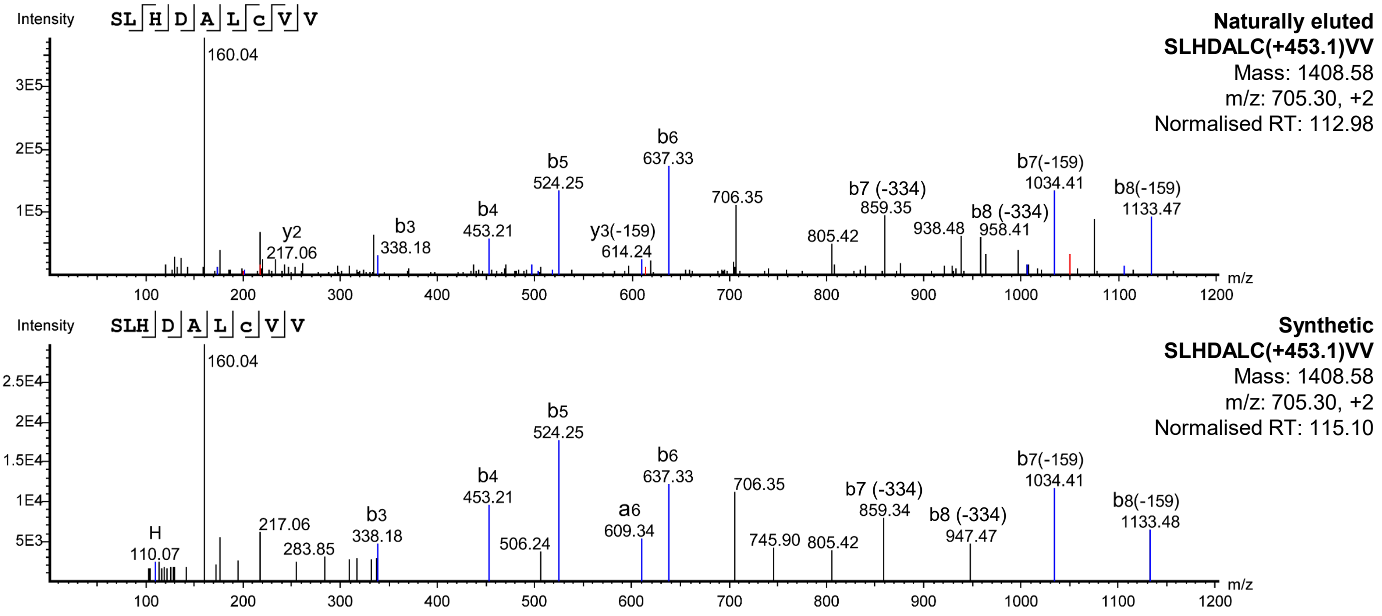

(B)

Ion	Predicted ions: Cysteinylated 119	Predicted ions: Cysteinylated Partial BP 294.06	Predicted ions: Cysteinylated Full BP 453.1	Ions in each spectra: Naturally eluted 119	Naturally eluted 294.06	Naturally eluted 453.1	Bottom, synthetic 119	Bottom, synthetic 294.06	Bottom, synthetic 453.1
b2		201.12			✓				
b3		338.18			✓			✓	
b4		453.21			✓			✓	
b5		524.25			✓			✓	
b6		637.33			✓			✓	
b7	859.34	1034.4	1193.4	✓	✓		✓	✓	
b8	958.41	1133.47	1292.51	✓	✓			✓	
y7	875.37	1050.43	1209.47	✓	✓				
y6	738.31	913.37	1072.41						
y3	439.16	614.22	773.26	✓	✓				
y2		217.1547			✓				
Drug		Thiazolidine ring - 160.04			✓			✓	
Drug		Penam core - 217.06			✓			✓	
Drug		Benzylpenicillin - 335.1			✓				

Supplementary figure 3: (A) Spectrum of naturally eluted SLHDALC(+453.10)VV (top), and synthetic SLHDALC(+453.10)VV (bottom). **(B)** Table listing the predicted m/z values of b and y ions from SLHDALC(+453.10)VV containing cysteinylated cysteine (+119.00), cysteinylated cysteine with partial adduct (+294.06), and cysteinylated cysteine with full adduct (+453.10). The table also includes the presence of these ions within the spectra of either the naturally eluted SLHDALC(+119.00)VV, naturally eluted SLHDALC(+453.10)VV, and synthetic SLHDALC(+453.10)VV.

S. Fig 4

(A)

(B)

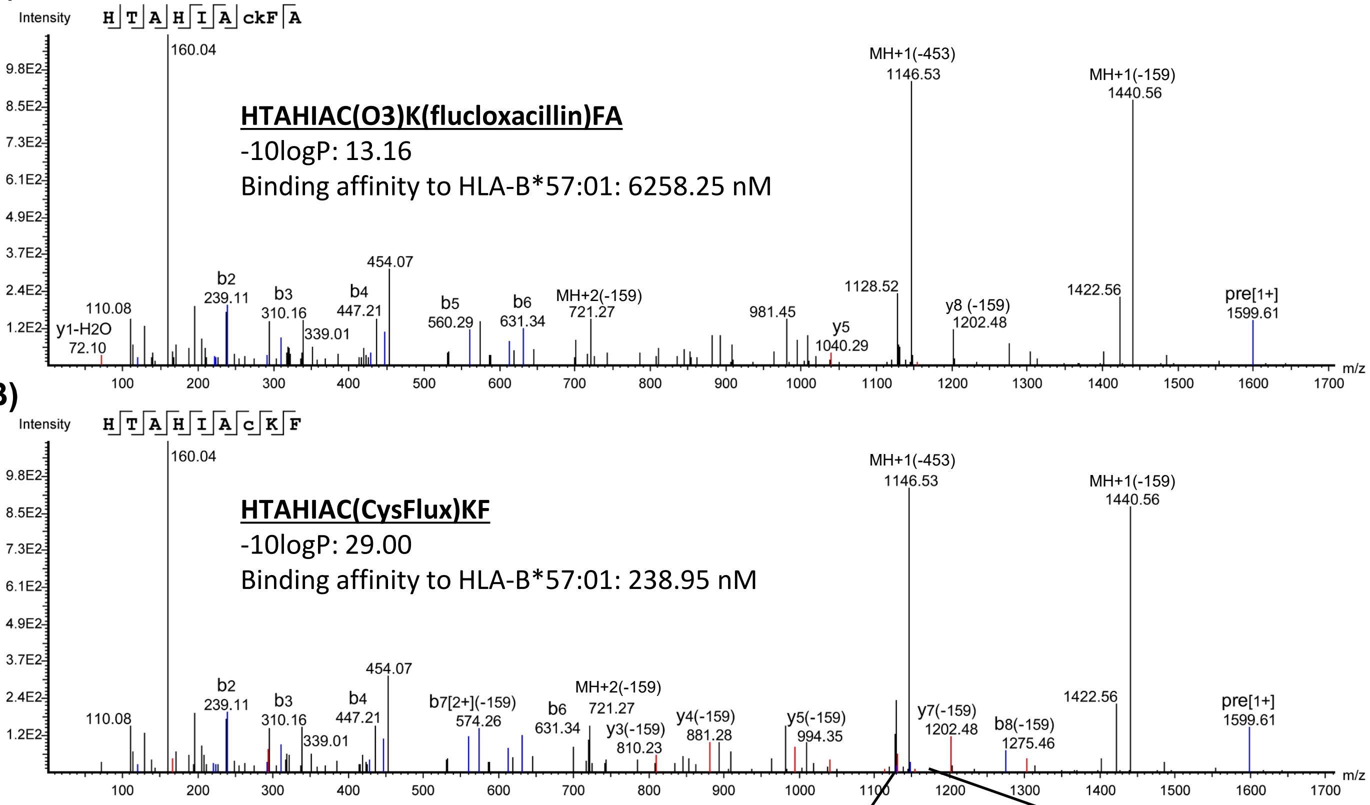

(C)

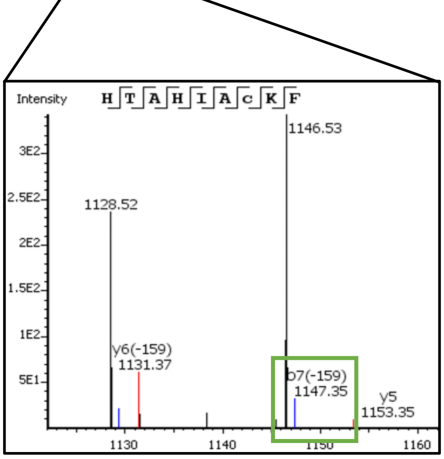

Spectrum information:

- Scan: 14093 (Within mgf file)
- Precursor: m/z 533.8625 (Observed), 2+
- 20161213_Pat_C1RB5701FluxLiv_1_pool5

Supplementary figure 4: Fragmentation of the precursor ion m/z 533.8625 reported by Waddington *et al.* 2020. (A) Spectra sequence assignment made in Waddington *et al.* and **(B)** spectra sequence assignment made in this study. The presence of b7 (-159, Thiazolidine ring) supports the hypothesis that haptenation is occurring on a cysteinylated cysteine in our study. **(C)** Information of the spectra used in this example.

S. Fig 5

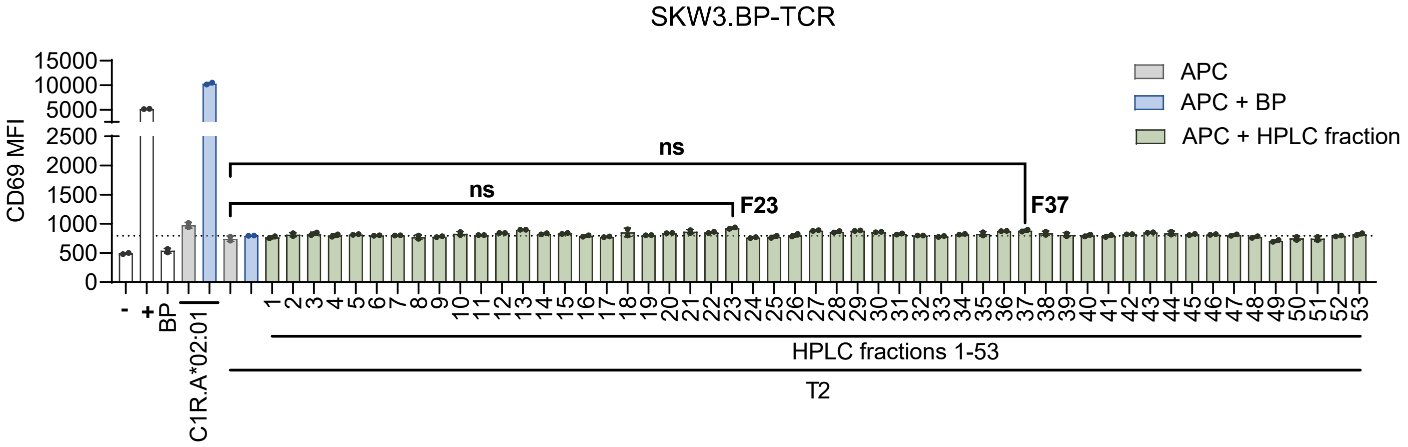

Supplementary figure 5: Activation assays measuring CD69 surface upregulation of SKW3.BP-TCR in response to T2 loaded with HLA-A*02:01 derived peptides from C1R.A*02:01 without BP treatment. This experiment was conducted in duplicate, mean +/- SEM (ns = not significant)**. -**: Media only, **+**: anti-CD3/CD28 beads.

S. Fig 6

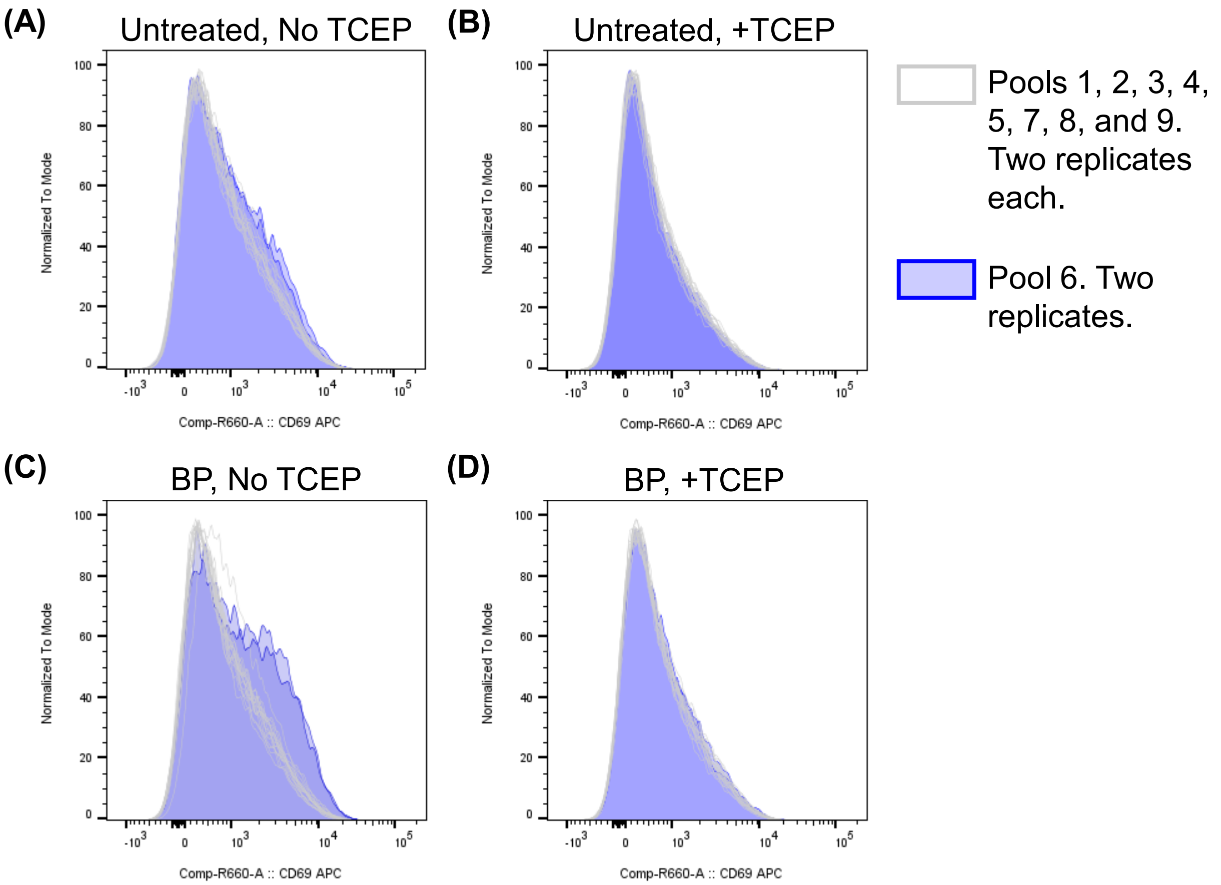

Supplementary figure 6: Histogram representation of SKW3.BP-TCR CD69 upregulation after stimulation with peptides isolated from C1R.A*02:01. Peptides from untreated and BP-treated C1R.A*02:01 were isolated, fractionated, and pooled to generate 9 pools of peptide from untreated cells, and 9 equivalent pools from BP-treated cells. Half of each pool was reduced with TCEP and the other half was not. Pools from **(A)** untreated C1R.A*02:01, **(B)** untreated C1R.A*02:01 + TCEP reduction, **(C)** BP-treated C1R.A*02:01, and **(D)** BP-treated C1R.A*02:01 + TCEP reduction, were loaded onto T2 cells prior to SKW3.BP-TCR stimulation. Pool 6, which is immunogenic when isolated from BP-treated cells without TCEP treatment, is coloured in blue. Experiment was conducted in technical duplicate.

S. Fig 7

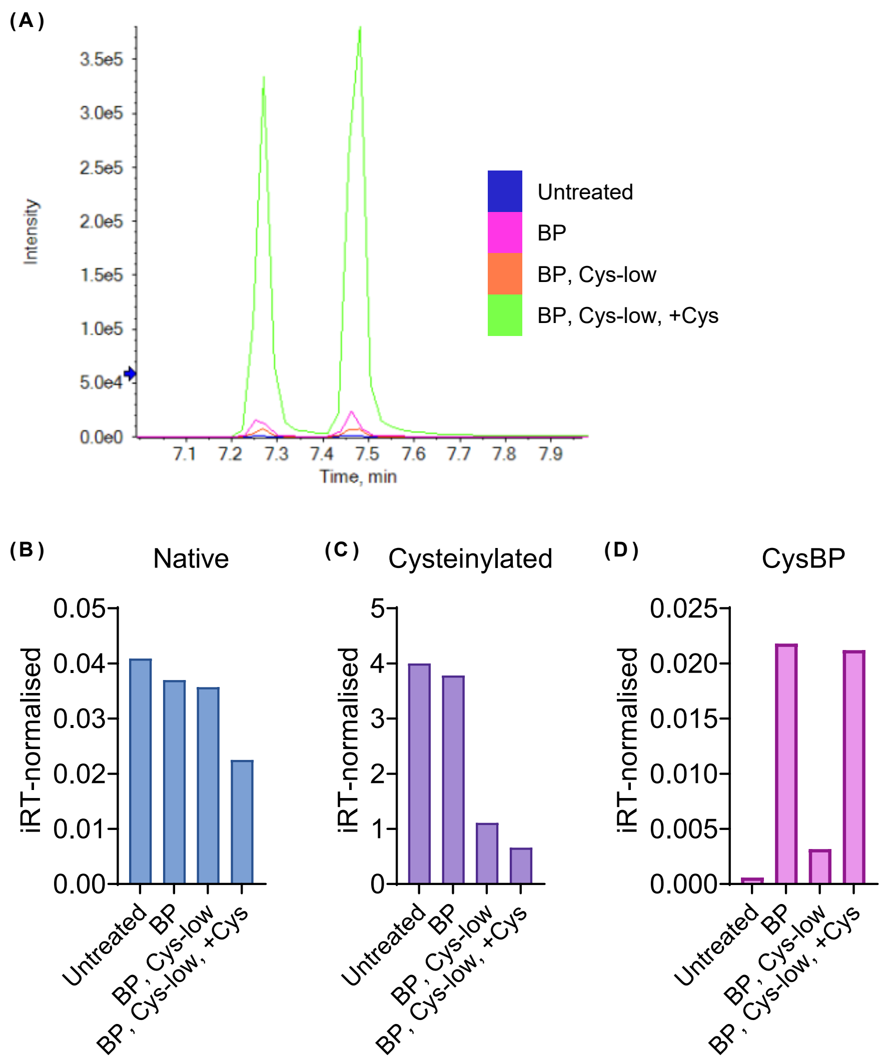

Supplementary figure 7: Formation of CysBP and CysBP adducts in Cys-rich and Cys-low media. (A) Extracted ion chromatogram analysis of cell culture supernatants for the presence of CysBP (m/z 456.13) in the 4 different conditions. Untreated = Regular RF10; BP = Regular RF10 with BP added; BP, Cys-low = cysteine-low RF10 with BP added; BP, Cys-low, +Cys = cysteine low RF10 with BP and cysteine added. **(B-D)** $1x10^8$ C1R.A*02:01 cells were cultured either in media containing cysteine (Untreated and BP), in cysteine-low media (BP, Cys-low), or cysteine-low media supplemented with cysteine (BP, Cys-low, +Cys), for 4 hours. pHLA complexes were isolated and eluted for quantitative LC-MS based measurement of LLPPPPCPA in its **(B)** native, **(C)** cysteinylated, or **(D)** CysBP-modified state across all conditions.
