# Supplementary material for: HLA‐A*02:01 Presents Penicillin‐Modified Cysteinylated Peptides for T Cell Recognition
Source: Allergy. 2025 Sep 4;80(11):3165–77. doi: 10.1111/all.70025 (PMC12590334; doi:10.1111/all.70025)
Supplement: Supplementary file 3 — Appendix S3: Supporting Information. [file ALL-80-3165-s002.docx]

### Patient information

Patient AH002 was recruited from the Allergy Clinic at The Alfred Hospital (Melbourne, Victoria, Australia) based on their clinical history of angioedema a day following penicillin exposure at the age of 18. ImmunoCAP testing in 2010 showed no antibodies towards amoxicilloyl and penicilloyl G but positive responses to ampicilloyl (0.381IU/mL) and penicilloyl V (0.47IU/mL), indicative of penicillin allergy. Blood samples for this study were collected when the individual was 58 years old in 2015. HLA genotyping (A*02:01/11:01, B*07:02/53:01, C*04:01/07:02, DRB1*02:01/13:02, DQB1*03:01/06:04, DPB1*02:01/03:01) was performed using next generation sequencing by the Victorian Transplantation and Immunogenetics Service (West Melbourne, Victoria, Australia). The study participant provided written consent, with ethics approvals granted by The Alfred Hospital (HREC 127/03) and Monash University (HREC 4717).

### β-lactam antibiotics

6-aminopenicillanic acid, benzylpenicillin (BP) sodium salt, ampicillin sodium salt, and piperacillin sodium salt were obtained from Sigma-Aldrich (MO, USA). Amoxicillin trihydrate was obtained from Sandoz Australia (NSW, Australia). Flucloxacillin was synthesised by Wockhardt, UK. BP was freshly solubilised (25 mM) in serum free RPMI 1640 (RPMI; Gibco, Life Technologies, NY, USA) at the time of assay. 6-aminopenicillanic acid, amoxicillin, flucloxacillin and piperacillin were freshly solubilised (4 mM) in RF10 (RPMI supplemented with 10% foetal calf serum [FCS] [Sigma-Aldrich], 5 % supplementum completum [2 mM MEM non-essential amino acids [Gibco], 100 mM HEPES [Gibco], 40 mM L-glutamine [Gibco], 1 mM β-mercaptoethanol [Sigma-Aldrich] in RPMI 1640) at the time of assay.

### Antigen-presenting cells and HLA expression

Cell lines were maintained in RF10. C1R.A*02:01 ^1^, C1R.A*11:01 ^2^, and C1R.B*07:02 ^3^ transfectants were generated and described elsewhere from the HLA class I-reduced B-lymphoblastoid cell line (B-LCL) C1R, which has low surface expression of HLA-B*35:03 and normal levels of HLA-C*04:01 ^4,5^. C1R.A*11:01 and C1R.B*07:02 co-expressed GFP. IHW 9010, 9038, and 9065 B-LCLs were sourced through the International Histocompatibility Working Group repository. Transporter associated with antigen processing (TAP) involvement in T cell activation was evaluated using the TAP-deficient cell line, T2 ^6^.

Maintenance of HLA-A*02:01 expression by C1R.A*02:01 during cell culture was facilitated by the selection antibiotic hygromycin B, at a concentration of 0.3 mg/mL (Sigma-Aldrich). HLA expression of HLA transfectants (compared to parental C1R) and B-LCLs was measured via flow cytometry by indirect staining with in-house produced primary antibody anti-pan HLA class I (W6/32) ^7^, anti-HLA-A2 (BB7.2) ^8^, anti-HLA-B7, HLA-B27 (ME-1) ^9^, or anti-HLA-C (DT9) ^10^, followed by secondary antibody goat anti-mouse IgG phycoerythrin (PE) (Southern Biotech, Birmingham, AL). A total of 30000 stained cells were acquired on a LSRII flow cytometer (Becton Dickinson [BD], San Jose, CA) located at the FlowCore facility (Monash University, Clayton, Victoria, Australia). Flow cytometry data were analysed using FlowJo (version 10; BD). HLA typing of antigen-presenting cells (APCs) is summarised in **Supplementary table 3.**

### Generation of Epstein-Barr virus-transformed autologous B-LCLs

To generate autologous B-LCLs from patient AH002, 1 mL of Epstein-Barr virus supernatant collected from the B95.8 cell line ^11^ was used to treat 5x10^6^ peripheral blood mononuclear cells (PBMCs) in 5 mL RF10 ^12^. Cells were cultured in a 96 well plate (150 μL/well) and incubated at 37°C, 5% CO_2_. To promote B cell expansion and immortalisation, 0.5 μg/mL and 0.45 μg/mL Cyclosporin A (C3662, Sigma-Aldrich) was added to cultures on days 2 and 9 respectively. Expanded B-LCLs were subsequently maintained in RF10.

### Isolation of peptides presented by HLA-A*02:01

Prior to drug treatment, cell cultures were transitioned into RF5 (same constituents as RF10 but with 5% v/v heat-inactivated FCS). For drug treatment, cell cultures were incubated with 2 mM BP for 48 hours (37°C, 5% CO_2_) prior to harvesting. Cells were pelleted by centrifugation and subsequently washed with phosphate buffered saline (PBS) twice before snap freezing in liquid nitrogen.

To isolate peptide-HLA complexes, C1R.A*02:01 cell pellets were lysed in a non-denaturing lysis buffer containing 0.5% IGEPAL-630 (Sigma-Aldrich), and HLA-A*02:01 were immunoaffinity purified by solid-phase bound anti-HLA-A2 antibody BB7.2 as described previously ^13,14^. Peptide-HLA complexes were dissociated with 10% acetic acid. For peptide separation from the heavy chain and β-2-microglobulin (β2m), eluates were fractionated offline by reversed-phase high-performance liquid chromatography (RP-HPLC) as described previously ^15^. Samples were separated at a flowrate of 2 mL/min into 500 µL fractions using the following condition with buffer A (0.1% Trifluoroacetic acid [TFA]) and increasing gradient of buffer B (0.1% TFA, 80% Acetonitrile [ACN]): 0-0.25 min 15% buffer B, 0.25-4.25 min 30% buffer B, 4.25-12.25 min 40% buffer B, 12.25-22.25 min 45% buffer B, 22.25-24.25 min 99% buffer B, 24.25-26.25 min 100% buffer B, 26.25-32.25 min 2% buffer B. The elution time of BP was determined by analysis of 5 μg BP using the same protocol. Protein, peptide, and BP elution timings were detected by 215nm UV absorption and 53 fractions prior to β2m elution were used in downstream assays.

For immunopurification 1, peptides were isolated from 4x10^9^ C1R.A*02:01 cells (either untreated or BP-treated), fractions were vacuum concentrated and formed into 9 pools prior to the β2m peak, and 1 pool after, which was passed through a 5 kDa filter. Pools were vacuum concentrated, and reconstituted in 0.1% FA. 50% of the material was used for MS analysis and 200 fmol of indexed retention time alignment (iRT) peptides ^16^ was added to each pool prior to analysis. The remaining 50% of pools 1-9 (fractions before the β2m peak) were used for SKW3.BP-TCR stimulation. Fractions were reconstituted in 20 µL 0.1% FA. Pools were spiked with 20 pmol NLV peptide, divided in two, and incubated 30 min at 37 °C with 10 mM TCEP or equivalent RPMI, prior to neutralisation to a final concentration of 5mM HEPES in 20 µL for SKW3 stimulation.

For immunopurification 2, peptides were isolated from 4x10^9^ C1R.A*02:01 cells (either untreated or BP-treated). All of the fractions were vacuum concentrated and made up in 0.1% FA. The first 50% of materials from each fraction were used for SKW3.BP-TCR stimulation to identify which fraction contained the immunogenic ligand. The other 50% of the immunogenic fractions were then analysed by mass spectrometry.

### Mass spectrometry analysis of HLA-bound peptides and sequence assignment

Mass spectrometry acquisition was performed on either a Q-Exactive Plus Hybrid Quadrupole Orbitrap mass spectrometer or Orbitrap Fusion Tribrid mass spectrometer coupled to a Dionex UltiMate 3000 RSLCnano UHPLC system (Thermo Fisher Scientific, USA). Samples were first loaded onto a PepMap Acclaim 100 C18 trap column (100 μm x 2 cm, nanoViper, C18, 5 µm, 100Å [Thermo Fisher Scientific]) in 0.1% formic acid (FA), 2% ACN and separated on a PepMap Acclaim RSLC HPLC Column (75 μm x 50 cm, nanoViper, C18, 2 µm, 100Å [Thermo Fisher Scientific]) at a flow rate of 250 μL/min using a gradient of increasing buffer B (0.1% FA, 80% ACN).

For both MS instruments, data were collected in positive ion mode with the following parameters: MS1 – Resolution: 70000, Scan range: m/z 300-1800. MS2 – Resolution: 17500, Fixed first mass: m/z 100. A maximum of 12 MS2 spectra were collected per cycle with a dynamic exclusion of 15 s.

MS/MS data were searched with PEAKS Xpro 10.6 (Bioinformatics Solutions Inc.) against the reviewed human proteome (UniProtKB/Swissprot, downloaded on 10/11/2018). All files went through default data refinement defined by PEAKS Xpro prior to a *de novo*-assisted database search. Instrument selected was Orbitrap (Orbi-Orbi). A parent mass error tolerance of 20 ppm and fragment mass error tolerance of 0.02 Da were allowed. For all searches, enzyme specificity was turned off. A maximum of 3 variable modifications were allowed to be assigned per peptide. The 5 variable modifications considered were oxidation of methionine (+15.99), deamidation of asparagine/glutamine (+0.98), cysteinylation of cysteine (+119.00), BP modification on cysteinylated cysteine (CysBP) (+453.10), and BP modifications on lysine/arginine/histidine/cysteine (+334.10). All analyses were conducted with a peptide false discovery rate (FDR) cut-off of 5%, which was estimated using a decoy fusion method ^17^. To validate peptide-spectrum matches assigned as BP-modified, data were exported in an *.mzxml* format and parsed through a custom produced tool coined PenicillinFinder (https://github.com/PurcellLab/PenicillinFinder). PenicillinFinder was written using the C programming language to identify defined diagnostic fragment ions of penicillin within the MS/MS spectra. The *.csv* output of PenicillinFinder containing peptide sequence, modifications, m/z, mass, retention time, presence of diagnostic ions, and protein of origin, was used for downstream analyses. Only spectra containing the m/z 160.04 ion, and at least one other BP-indicative fragment ion (m/z 217.06 or 335.11) were considered as *bona fide* drug-modified peptide spectra. Spectra containing diagnostic ions but i) assigned to incorrect modification sites within the peptide sequence (determined by manual spectral analyses), ii) assigned more than one BP modification, iii) ambiguous modification location, or iv) assigned peptides with poor quality MS/MS spectra (*i.e.* weak diagnostic ions [<10% of most intense ion], diagnostic ions annotated as b or y ions) were not included in assessments of haptenation patterns. Peptide overlap, peptide length distribution, and peptide motif analyses of the total immunopeptidome were conducted using only non-redundant sequences. All BP-modified peptide analyses were conducted with peptides unique by sequence and modifications. Bar charts, heat maps, point charts, and mirror plots were generated using Prism 9.0, GraphPad (San Diego, CA). HLA binding predictions were performed using NetMHCpan-4.1b ^18^. All mass spectrometry data have been deposited to the ProteomeXchange via the PRIDE partner repository ^19^ with the identifier PXD057177 and PXD065200.

### Bioinformatic re-analysis of HLA-B*57:01 flucloxacillin dataset*.*

Waddington *et al.* analysed the HLA-B*57:01 immunopeptidome after cellular treatment with flucloxacillin and have deposited the data on the ProteomeXchange Consortium (<https://www.ebi.ac.uk/pride/archive/projects/PXD020137>), which were re-analysed with PEAKS Xpro 10.6, via database search against the reviewed human proteome. All files went through default data refinement defined by PEAKS Xpro prior to *de novo*-assisted database search. Instrument selected was Triple TOF. A parent mass error tolerance at 50 ppm and fragment mass error tolerance at 0.1 Da were allowed. For all searches, enzyme specificity was turned off. A maximum of 3 variable modifications were allowed per peptide. Variable modifications considered were oxidation of methionine (+15.99), deamidation of asparagine/glutamine (+0.98), cysteine oxidation to cysteic acid (+47.98), cysteinylation of cysteine (+119.00), flucloxacillin modification on cysteinylated cysteine (CysFlux) (+572.06), and flucloxacillin modifications on lysine/arginine/cysteine (+453.06). Peptides were considered flucloxacillin-modified if peptide spectra contained the diagnostic ion m/z 160.04 ^20^.

### In vitro generation of peptides with benzylpenicillin modifications on cysteinylated cysteines

Peptides of interest were synthesised by Mimotopes (Mulgrave, Victoria, Australia) in their native forms. To generate cysteinylated peptides containing BP adducts, peptides were first incubated with L-cysteine (Sigma Aldrich) at a molar ratio of 1:4 (Peptide:L-cysteine) for 3 hours at 37°C in PBS, followed by BP at a molar ratio of 1:2 (Peptide/L-cysteine:BP) for 24 hour at 37°C. Prior to MS analysis, peptides were desalted using OMIX C18 pipette tips (Agilent, CA, USA). Peptide MS/MS spectra were acquired using the same parameters used for the natural HLA peptide ligands. Synthetic and naturally eluted peptide spectral comparisons using all ions were performed using Universal Spectrum Explorer ^21^.

### Peripheral blood mononuclear cell isolation and *in vitro* expansion of drug-specific T cells

Patient-derived PBMCs were isolated from peripheral whole blood using Ficoll-Paque (GE Healthcare, Uppsala, Sweden) density centrifugation and cryopreserved in liquid nitrogen (-196°C) until required. For expansion of drug-specific T cells, PBMCs were thawed at 37°C, washed and resuspended in RH10 (Same constituents as RF10 but with 10% heat-inactivated human blood group AB serum [Sigma-Aldrich]). Recovered PBMCs were stimulated at a density of 5x10^6^ cells in 2 mL RH10 with 0.5 mM BP on day 0. T cell cultures were supplemented with 50 U/mL of recombinant human IL-2 (Peprotech, NJ, USA) on days 4 to 14 as required to promote optimal cell outgrowth. On day 14, the T cell line was further restimulated with autologous PBMCs (irradiated at 3000 Rads) and BP at a final concentration of 0.5 mM in RH10, and allowed to expand for another 14 days. On day 28, cells were either cryopreserved or used immediately for T cell restimulation assays (referred to as d28 T cells).

### Drug-pulsed APCs and T cell activation

Drug-pulsed APCs were generated by incubating 1x10^6^ cells in RH10 with 0.5 mM drugs for 24 hours at 37°C, 5% CO_2_. Cells were washed twice to remove unbound drug and resuspended in RH10 for d28 T cell stimulation. To evaluate T cell activation, d28 T cells (2x10^5^) were restimulated with 1x10^5^ of either 9038 alone, 9038 in the presence of drug (0.5 mM) and drug-pulsed 9038 (0.5 mM). Controls including Dynabeads® Human T-Activator CD3/CD28 beads (positive control; Thermo Fisher Scientific), media (negative control), and drug alone (T-T killing test, 0.5 mM), were included. For intracellular cytokine staining (ICS) for the production of pro-inflammatory cytokines, IFNγ and TNF ^22^, a stimulation was first performed for six hours at 37°C, 5% CO_2_ with Brefeldin A (10 µg/mL; Sigma-Aldrich) being added for the last 4 hours of the co-incubation. To detect T cell subsets, cells were surface stained with a mixture of CD4 PE (clone RPA-T4), and CD8 PerCP-Cy5.5 (clone SK1) antibodies and LIVE/DEAD® fixable Aqua stain (Life Technologies). Cells were then fixed in 1% paraformaldehyde (ProSciTech, Australia) in PBS, then permeabilised and stained intracellularly with IFNγ PE-Cy7 (clone B27) and TNFα V450 (clone MAb11) antibodies in 0.3% Saponin (Sigma-Aldrich). A maximum of 50,000 cells were acquired on the LSRII flow cytometer (BD) at the FlowCore facility (Monash University). Data were analysed using FlowJo (version 10; BD) **(Figure S1)**.


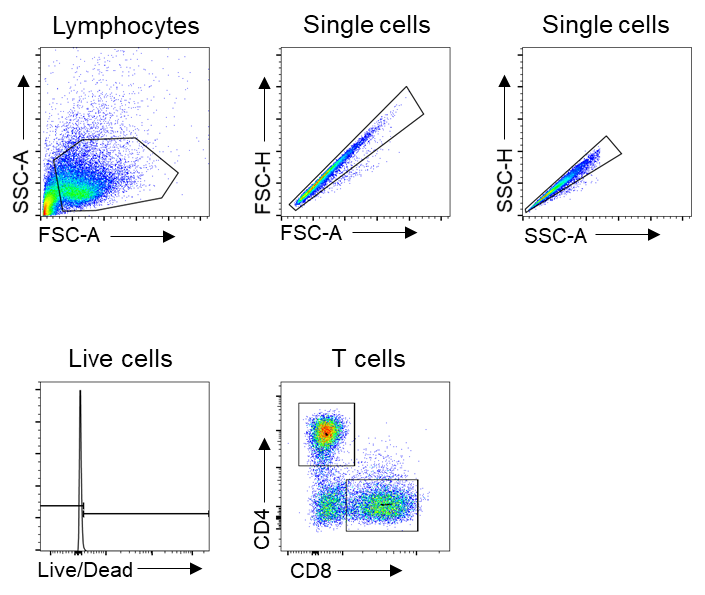


**Figure S1:** Gating strategy to evaluate the specificity of d28 T cells expanded from hypersensitive patient AH002. Data were acquired on the BD LSR II flow cytometer (BD Biosciences) at the Flowcore facility (Monash University, Clayton). Data were analysed using Flowjo v10 (FlowJo LLC, BD Biosciences).

### αβTCR repertoire profiling of benzylpenicillin-specific CD8^+^ T cells

The αβTCR signature of d28 drug specific T cells was assessed by single-cell sorting of T cells exhibiting drug-induced cytokine production as assessed by a IFNγ Secretion Assay Detection Kit (Allophycocyanin; Miltenyi Biotec, USA) according to the manufacturer’s instructions. Here, d28 T cells (1x10^7^) were restimulated with drug-pulsed 9038 at a responder to stimulator ratio of 2 : 1 in RH5 for 4 hours (37°C, 5% CO_2_). To study the αβTCR signatures of activated T cells, IFNγ allophycocyanin catch reagent antibody (Miltenyi Biotec) was added according to the manufacturer’s instructions and cells were co-stained with CD8 FITC (HIT8a; Thermo Fisher Scientific). Stained sample was single cell sorted into 96-well PCR plates (Axygen, USA) on a BD Influx flow cytometer (FlowCore, Monash University) for the following populations: CD8^+^IFNγ^-^, and CD8^+^IFNγ^+^. Plates were stored immediately at -80°C until required.

Reverse transcription-polymerase chain reaction (RT-PCR), followed by multiplex nested PCR and sequencing of TCRα and β chains were performed as previously described ^3,22,23^. All TCR sequences were aligned on the ImMunoGeneTics (IMGT)/V-QUEST web-based tool against the IMGT database containing known TCR sequences with nomenclature based on Lefranc *et al.* ^24^, and analysed with TCR_Explore ^25^. The CDR3 regions of TCRs reported here start from position 3, an equivalent of amino acid position 107 of the variable segments, which ends at either position 10 of TRAJ, or position 6 of TRBJ. High confidence sequences with ≥85% identity against TCR genes were included for analyses; detailed results can be found in **Supplementary table 6**.

### Generation of SKW3.BP-TCR reporter cell line

To characterise the αβTCR of interest (BP-TCR), we generated a cDNA construct encoding the full length TCRα and TCRβ chains; TRAV3, CDR3α – CAVRDNRNYGQNFVF, TRAJ26, TRBV20-1, CDR3β – CSARTDREGQPQHF, TRBJ1-5. The TCR was designed with a self-cleaving 2A linker peptide that induces a ribosomal skip between the TCR α and β chain. This construct was cloned into the pMSCV-IRES-GFP (pMIG) retroviral vector as described previously ^26,27^. The vector was transduced into the TCR-null cell line SKW3.hCD8αβ.GFP^+^ using HEK293T packaging cells, 4 μg of pPAM-E ^28^, 2 μg of pVSV-G (Clontech Laboratories, USA), and 20 μL Lipofectamine 3000 Reagent (Thermo Fisher Scientific) ^3^. Importantly, SKW3.hCD8αβ.GFP^+^ cells contain all other CD3 chains and the required signalling components. The generated SKW3 cell line expressing the TCR of interest will be referred to as SKW3.BP-TCR. The original SKW3 cell line, has the HLA type as follows: HLA-A*11:01, 30:01; B*35:01, 44:02; C*04:01, 05:01; DRB1*01:03, 04:01; DQB1*03:01; DPB1*04:01, 04:02 ^22^. As a non-specific control, the published SKW3.LTR5.NLV, specific for the CMV epitope NLV (NLVGMVATV) restricted to HLA-A*02:01 was included in activation assays ^3^. All SKW3 cell lines were maintained in RF10. Successful surface expression of TCRs on SKW3 cells was determined by CD3 upregulation
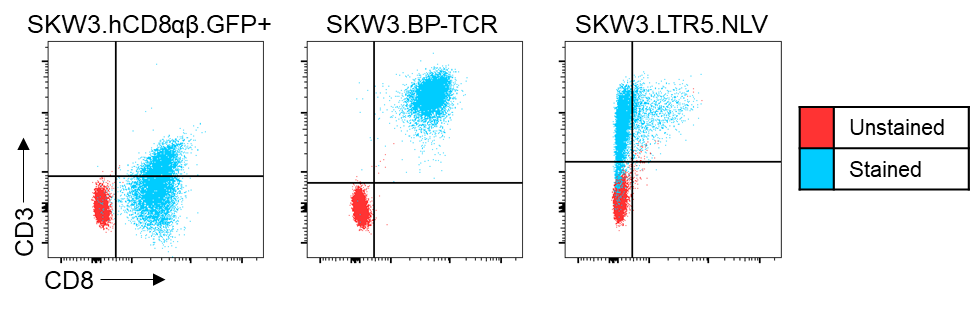
when compared to the parental SKW3.hCD8αβ.GFP^+^ **(Figure S2)**.

**Figure S2:** SKW3 cell lines were periodically tested for TCR cell surface expression based on CD3 expression. Data were acquired on the BD LSR II flow cytometer (BD Biosciences) at the Flowcore facility (Monash University, Clayton). Data were analysed using Flowjo v10 (FlowJo LLC, BD Biosciences).

### Functional T cell assays with SKW3 cells

Activation of SKW3.TCR cell lines was assessed by cell surface CD69 upregulation after stimulation with APCs for 17-20 hours at 37°C, 5% CO_2_ at a 1:1 ratio (1x10^5^ cells each) in the presence or absence of antigen (0.5 mM drug or 1 μM peptide unless otherwise stated). Drug-pulsed APCs were generated as described above for T cell stimulation assays. Monensin-treated APCs were generated by incubating cells (density of 1x10^6^ cells/mL) with 5 μM of monensin (M2573, Sigma-Aldrich) for 1 hour at 37°C, 5% CO_2_ in the dark. Cells were washed twice before co-incubation with SKW3 transfectants. To generate antibody-treated cells for blocking assays, untreated APCs and drug-pulsed APCs (0.1 mM drug) were pre-incubated with 10 μg/mL of purified BB7.2 (anti-HLA-A2) antibody for 1 hour at 37°C, 5% CO_2_. Cells were washed prior to co-incubation with SKW3 transfectants.

For stimulation with fractionated peptides isolated from HLA-A*02:01, T2 cells were pre-incubated at 27°C overnight. 10^5^ T2 cells were then loaded with 2 μL of reconstituted HLA-A*02:01-isolated peptide fractions for 1 hour at 27°C before co-incubation with SKW3 transfectants at 37°C, 5% CO_2_.

For stimulation with pooled HLA-A*02:01 peptides with and without TCEP treatment, neutralised peptide pools were loaded onto 5 x 10^5^ T2 cells for 1 hour at 27°C before co-incubation with SKW3 transfectants at a 1:1 ratio at 37°C, 5% CO_2._

After SKW3.BP-TCR stimulation, cells were pelleted and resuspended in a surface stain mixture including CD3 PE-Cy7 (clone SK7), CD8 PerCP-Cy5.5 (clone SK1) and CD69 allophycocyanin (clone L78) antibodies, and LIVE/DEAD® fixable Aqua stain in PBS and incubated for 30 min at 4°C. Cells were washed in PBS, fixed in 1% paraformaldehyde in PBS, and resuspended in PBS for data acquisition. A maximum of 50,000 cells were acquired on the BD LSR II flow cytometer (BD Biosciences) at the FlowCore facility (Monash University). Data were analysed using FlowJo v10 (FlowJo LLC, BD Biosciences).

### Quantitative mass spectrometry analysis of LLPPPPCPA within the immunopeptidome

The abundance of LLPPPPCPA in its native, cysteinylated, and CysBP-modified forms were quantitatively analysed using a full scan multiple reaction monitoring (MRMhr) approach. C1R.A*02:01 cells were either grown in RF10, or in cysteine-low RF10 (cystine free RPMI-1640 media [R7513, Sigma-Aldrich] supplemented with 10% dialysed FCS [A3382001, Thermo Fisher], and 5% supplementum completum). To determine the role of extracellular cysteine in forming CysBP-modified LLPPPPCPA, L-cysteine [Sigma-Aldrich] was added back into the cysteine-low RF10 to a final concentration of 0.21 mM. HLA peptides were isolated as described above and acquired using a ZenoTOF 7600 LC-MS system (SCIEX) coupled to an M Class UHPLC (Waters). Peptides were directly injected onto a 15 cm Aurora Elite SX column (IonOpticks). Samples were separated at a flowrate of 150 nL/min for a total run time of 175 min using buffer A (0.1% FA) and an increasing gradient of buffer B (0.1% FA, 100% ACN). An active 90 min gradient with increasing levels of buffer B (1% to 35%) was applied to separate the analytes. TOF-MS was acquired for 150 ms, and then MRMhr TOF-MS/MS spectra were acquired according to Table S1. Peptide peak areas were normalised to the MS1 peak area of iRT I (GTFIIDPGGVIR).

| **Table S1:** MRMhr targets for LLPPPPCPA quantification. | | | | | | | | |
| --- | --- | --- | --- | --- | --- | --- | --- | --- |
| **Peptide** | **precursor (m/z)** | **TOF start** | **TOF stop** | **Accumulation time** | **DP** | **CE** | **Fragmentation mode** | **Kinetic Energy (EAD)** |
| LLPPPPCPA | 904.49 | 90 | 1800 | 0.05 | 80 | 48 | CID | 0 |
| LLPPPPCPA | 452.751 | 90 | 1800 | 0.05 | 80 | 19 | CID | 0 |
| LLPPPPC[Cysteinylated]PA | 512.25 | 90 | 1800 | 0.05 | 80 | 22 | CID | 0 |
| LLPPPPC[Cysteinylated]PA | 512.25 | 90 | 1800 | 0.07 | 80 | 22 | EAD/CID | 5 |
| LLPPPPC[CysBP]PA | 679.3 | 90 | 1800 | 0.05 | 80 | 30 | CID | 0 |
| LLPPPPC[CysBP]PA | 453.2 | 90 | 1800 | 0.05 | 80 | 17 | CID | 0 |
| LLPPPPC[CysBP]PA | 679.3 | 90 | 1800 | 0.07 | 80 | 30 | EAD/CID | 5 |
| LLPPPPC[CysBP]PA | 453.2 | 90 | 1800 | 0.07 | 80 | 17 | EAD/CID | 5 |
| TOF: Time of flight  DP: Declustering potential  CE: Collision energy  CID: Collision induced dissociation  EAD: Electron activated dissociation | | | | | | | | |

### Analysis of the cell culture supernatant for CysBP formation

Supernatants of C1R.A*02:01 were retained after cell harvesting. To isolate metabolites within the supernatants from proteins, ice cold methanol was added to cell culture supernatants at a ratio of 1:4 and vortexed after. Samples were incubated at -20°C for 1 hour and subjected to centrifugation for 15 min at 4°C to collect crashed out proteins at the bottom of the tubes. The supernatants were transferred into fresh tubes, lyophilised, desalted using C18 OMIX tips (Agilent), and reconstituted in 0.1% FA. Samples were acquired using a TripleTOF® 6600 (SCIEX) coupled to an Eksigent Ekspert nanoLC 400 (SCIEX). Samples were directly injected into a Luna Omega 3 μM C18 100A, 50 x 0.3 mm, column at a flowrate of 10 μL/min for 15 min using the following condition with buffer A (0.1% FA) and an increasing gradient of buffer B (0.1% FA, 80% ACN): 0-2 min 3% buffer B, 2-9 min 95% buffer B, 9-11 min 95% buffer B, 11-12 min 3% buffer B, 12-15 min 3% buffer B. The mass spectrometer was operated in an information-dependent acquisition mode, acquiring up to 10 MS/MS spectra per cycle with an accumulation time of 250 ms and 100 ms for MS1 and MS2 respectively. Ions of 100–900 m/z were included for MS1 scans and at least 100 m/z for MS2 scans.

### Drug treatment and mass spectrometry analysis of C1R.A*02:01

C1R.A*02:01 cells in RF10 were treated with 2mM of BP for either 4 or 48 hours prior to harvesting. Cell pellets were mechanically lysed in a non-denaturing lysis buffer containing 0.5% IGEPAL-630 (Sigma-Aldrich) before end-over-end rotation for 45 min at 4°C. The supernatant of lysed cells was separated from cellular debris by centrifugation and protein concentration of samples measured by infrared spectrometry using a Direct Detect™ analyser (Merck-Millipore).

To determine if CysBP could form intracellularly, 50 μg of proteins were denatured with 8 M urea, prior to treatment with 10 mM TCEP or being left untreated. All samples were alkylated with 40 mM of iodoacetamide and digested with either trypsin or chymotrypsin overnight on a shaking incubator (60 RPM, 37°C). Samples were acidified to a final concentration of 1% FA. Prior to MS analysis, samples were desalted using C18 OMIX tips (Agilent) and reconstituted in 0.1% FA/2% ACN.

For MS analysis, 200 ng of tryptic peptides per condition were analysed on a ZenoTOF 7600 LC-MS system (SCIEX) coupled to an M Class UHPLC (Waters). Peptides were directly injected onto a 15 cm Aurora Elite SX column (IonOpticks). Samples were separated at a flowrate of 150 nL/min for a total run time of 175 min using buffer A (0.1% FA) and an increasing gradient of buffer B (0.1% FA, 100% ACN). An active 90 min gradient with increasing levels of buffer B (1% to 35%) was applied to separate the analytes.

MS/MS data were searched with PEAKS Xpro 10.6 (Bioinformatics Solutions Inc.) against the reviewed human proteome (UniProtKB/Swissprot, downloaded on 10/11/2018). All files went through default data refinement defined by PEAKS Xpro prior to a *de novo*-assisted database search. Instrument selected was Triple TOF. A parent mass error tolerance at 20 ppm and fragment mass error tolerance at 0.02 Da were allowed. Trypsin and chymotrypsin were selected as the enzyme for the respective samples. A maximum of 3 variable modifications were allowed per peptide. Variable modifications considered were oxidation of Met (+15.99), deamidation of Asn/Gln (+0.98), carbamidomethylation of Cys (+57.02), cysteinylation of Cys (+119.00), CysBP (+453.10), and BP modifications on Lys/Arg/His/Cys (+334.10). All analyses were conducted at a peptide FDR of 1% ^17^. Only assigned BP-modified peptide spectra containing the m/z 160.04 ion, and at least one other BP-indicative ion (m/z 217.06 or 335.11) were considered *bona fide*.

### Statistical analysis of functional analyses

All experiments were performed in technical triplicate and reported as mean ± standard error of mean unless stated otherwise. Statistical significance was determined by Student’s t-test, with a p-value of <0.05 being significant (Prism version 9.0, GraphPad, USA).

References

1. Schittenhelm RB, Sian TCLK, Wilmann PG, Dudek NL, Purcell AW. Revisiting the arthritogenic peptide theory: quantitative not qualitative changes in the peptide repertoire of HLA–B27 allotypes. *Arthritis & rheumatology*. 2015;67(3):702-713.

2. Habel JR, Nguyen AT, Rowntree LC, et al. HLA-A* 11: 01-restricted CD8+ T cell immunity against influenza A and influenza B viruses in Indigenous and non-Indigenous people. *PLoS pathogens*. 2022;18(3):e1010337.

3. Nguyen TH, Rowntree LC, Pellicci DG, et al. Recognition of distinct cross-reactive virus-specific CD8+ T cells reveals a unique TCR signature in a clinical setting. *The Journal of Immunology*. 2014;192(11):5039-5049.

4. Storkus WJ, Howell DN, Salter RD, Dawson JR, Cresswell P. NK susceptibility varies inversely with target cell class I HLA antigen expression. *J Immunol*. 1987/03/15/ 1987;138(6):1657–1659.

5. Zemmour J, A. M. L, D. J. S, Parham P. The HLA-A,B "negative" mutant cell line C1R expresses a novel HLA-B35 allele, which also has a point mutation in the translation initiation codon. *J Immunol*. 1992/03/01/ 1992;148(6):1941–1948.

6. Salter RD, Howell DN, Cresswell P. Genes regulating HLA class I antigen expression in TB lymphoblast hybrids. *Immunogenetics*. 1985;21(3):235-246.

7. Barnstable CJ, Bodmer WF, Brown G, et al. Production of monoclonal antibodies to group A erythrocytes, HLA and other human cell surface antigens-new tools for genetic analysis. *Cell*. 1978/05// 1978;14(1):9–20. doi:10.1016/0092-8674(78)90296-9

8. Parham P, Brodsky FM. Partial purification and some properties of BB7. 2 a cytotoxic monoclonal antibody with specificity for HLA-A2 and a variant of HLA-A28. *Human immunology*. 1981;3(4):277-299.

9. Ellis SA, Taylor C, McMichael A. Recognition of HLA-B27 and related antigen by a monoclonal antibody. *Hum Immunol*. 1982;5(1):49-59. doi:10.1016/0198-8859(82)90030-1

10. Braud VM, Allan DS, Wilson D, McMichael AJ. TAP- and tapasin-dependent HLA-E surface expression correlates with the binding of an MHC class I leader peptide. *Curr Biol*. 1998/01/01/ 1998;8(1):1–10. doi:10.1016/s0960-9822(98)70014-4

11. Miller G, Lipman M. Release of infectious Epstein-Barr virus by transformed marmoset leukocytes. *Proceedings of the National Academy of Sciences*. 1973;70(1):190-194.

12. Pratoomwun J, Thomson P, Jaruthamsophon K, et al. Characterization of T-Cell Responses to SMX and SMX-NO in Co-Trimoxazole Hypersensitivity Patients Expressing HLA-B* 13: 01. *Frontiers in immunology*. 2021:1397.

13. Pandey K, Ramarathinam SH, Purcell AW. Isolation of HLA bound peptides by immunoaffinity capture and identification by mass spectrometry. *Current Protocols*. 2021;1(3):e92.

14. Purcell AW, Ramarathinam SH, Ternette N. Mass spectrometry–based identification of MHC-bound peptides for immunopeptidomics. *Nature protocols*. 2019;14(6):1687-1707.

15. Thomson PJ, Illing PT, Farrell J, et al. Modification of the cyclopropyl moiety of abacavir provides insight into the structure activity relationship between HLA‐B* 57: 01 binding and T‐cell activation. *Allergy*. 2020;75(3):636-647.

16. Escher C, Reiter L, MacLean B, et al. Using i RT, a normalized retention time for more targeted measurement of peptides. *Proteomics*. 2012;12(8):1111-1121.

17. Zhang J, Xin L, Shan B, et al. PEAKS DB: de novo sequencing assisted database search for sensitive and accurate peptide identification. *Molecular & cellular proteomics*. 2012;11(4)

18. Reynisson B, Alvarez B, Paul S, Peters B, Nielsen M. NetMHCpan-4.1 and NetMHCIIpan-4.0: improved predictions of MHC antigen presentation by concurrent motif deconvolution and integration of MS MHC eluted ligand data. *Nucleic acids research*. 2020;48(W1):W449-W454.

19. Perez-Riverol Y, Bandla C, Kundu DJ, et al. The PRIDE database at 20 years: 2025 update. *Nucleic Acids Research*. 2025;53(D1):D543-D553.

20. Waddington JC, Meng X, Illing PT, et al. Identification of flucloxacillin-haptenated HLA-B* 57: 01 ligands: evidence of antigen processing and presentation. *Toxicological Sciences*. 2020;177(2):454-465.

21. Schmidt T, Samaras P, Dorfer V, et al. Universal spectrum explorer: a standalone (web-) application for cross-resource spectrum comparison. *Journal of proteome research*. 2021;20(6):3388-3394.

22. Mifsud NA, Illing PT, Lai JW, et al. Carbamazepine induces focused T cell responses in resolved Stevens-Johnson syndrome and toxic epidermal necrolysis cases but does not perturb the immunopeptidome for T cell recognition. *Frontiers in Immunology*. 2021;12:653710.

23. Wang GC, Dash P, McCullers JA, Doherty PC, Thomas PG. T cell receptor αβ diversity inversely correlates with pathogen-specific antibody levels in human cytomegalovirus infection. *Science translational medicine*. 2012;4(128):128ra42-128ra42.

24. Lefranc M-P. Nomenclature of the Human T Cell Receptor Genes. *Current Protocols in Immunology*. 2000/12/01/ 2000;40(1):A.1O.1–A.1O.23. doi:10.1002/0471142735.ima01os40

25. Mullan KA, Zhang JB, Jones CM, et al. TCR_Explore: a novel webtool for T cell receptor repertoire analysis. *bioRxiv*. 2022:2022.11.03.514642. doi:10.1101/2022.11.03.514642

26. Szymczak AL, Workman CJ, Wang Y, et al. Correction of multi-gene deficiency in vivo using a single 'self-cleaving' 2A peptide-based retroviral vector. *Nat Biotechnol*. 2004/05// 2004;22(5):589--594. doi:10.1038/nbt957

27. Rowntree LC, van den Heuvel H, Sun J, et al. Preferential HLA-B27 Allorecognition Displayed by Multiple Cross-Reactive Antiviral CD8+ T Cell Receptors. *Front Immunol*. 2020/02/19/ 2020;11doi:10.3389/fimmu.2020.00248

28. Holst J, Szymczak-Workman AL, Vignali KM, Burton AR, Workman CJ, Vignali DAA. Generation of T-cell receptor retrogenic mice. *Nat Protoc*. 2006/// 2006;1(1):406--417. doi:10.1038/nprot.2006.61
